# Biomimetic separator design through naphthalene/glass fiber integration to achieve dendrite-growth-suppressed Zn anodes for high-performance aqueous zinc-ion batteries

**DOI:** 10.1039/d6ra07297f

**Published:** 2026-09-11

**Authors:** Guanqian Huang, Jinxiong Liang, Zhanpeng Yang, Yingfei Wu, Haijun Peng

**Affiliations:** a School of Intelligent Manufacturing, Guangdong University of Science and Technology Dongguan Guangdong 523083 China 2429771484@qq.com Penghj1989@163.com

## Abstract

Aqueous Zn-ion batteries (ZIBs) hold immense potential for sustainable, large-scale grid energy storage. However, the practical application of ZIBs is hindered by the instability of Zn anodes caused by uncontrollable dendrite growth and parasitic reactions with aqueous electrolytes. Herein, we propose a strategy to regulate Zn^2+^ flux *via* biomimetic separator integration. Biomimetic naphthalene/glass fiber separators (NtGF) were successfully constructed through one-step vacuum filtration. The interaction between Zn^2+^ and Nt molecules facilitates the preferential capture of Zn^2+^ by Nt molecules, which effectively boosts Zn^2+^ ionic conductivity, homogenizes the electric-field and Zn^2+^ concentration-field distributions, and thereby inhibits Zn dendrite formation while suppressing side reactions. Remarkably, symmetric Zn/NtGF/Zn cells demonstrate exceptional stability over 1480 h under current densities ranging from 0.5 mA cm^−2^ to 10 mA cm^−2^. Furthermore, the fabricated Zn/NtGF/NH_4_V_4_O_10_ full battery delivers a specific capacity of 266.5 mAh g^−1^ and retains 91.3% of its initial capacity after 1000 cycles at a high current density of 5 A g^−1^. This work underscores the critical role of biomimetic modification in designing advanced aqueous ZIBs.

## Abstract 2

Biomimetic NtGF separators capture Zn^2+^*via* π-electrons, homogenizing Zn^2+^ flux to suppress dendrites and side reactions. Zn//Zn cells cycle over 1480 h at 0.5–10 mA cm^−2^. Zn//NH_4_V_4_O_10_ battery retains 91.3% of its initial capacity after 1000 cycles.

## Graphical abstract

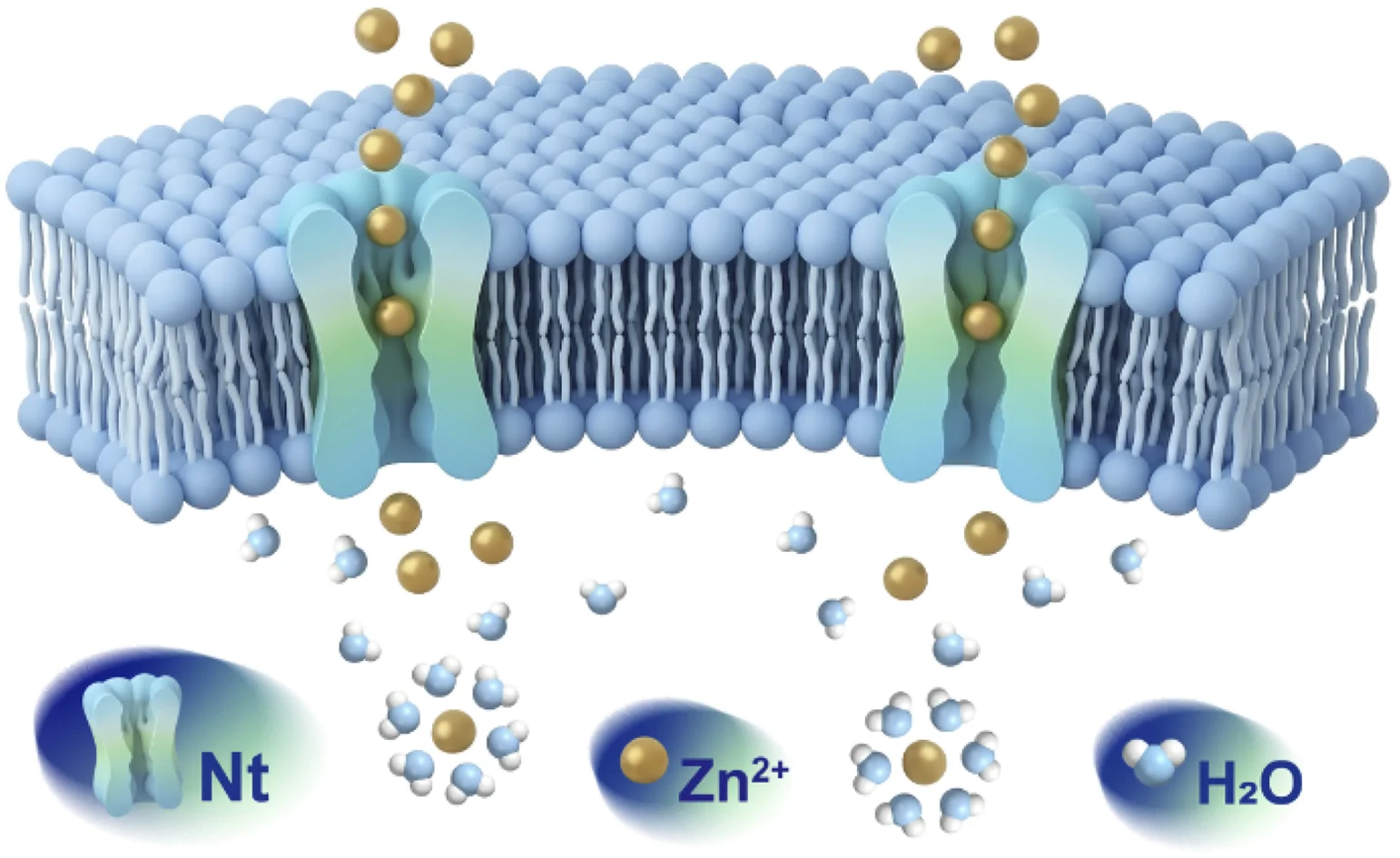

## 1. Introduction

Aqueous zinc-ion batteries (AZBs) have emerged as promising candidates for large-scale grid energy storage, driven by the inherent high theoretical capacity (820 mAh g^−1^),^1–3^ low redox potential (−0.76 V *vs.* SHE, standard hydrogen electrode),^4–6^ cost-effectiveness and crustal abundance of Zn metal anodes.^7,8^ However, the practical deployment of ZIBs is critically hindered by the thermodynamic instability at the Zn anode/electrolyte interface, leading to parasitic reactions and uncontrollable dendrite growth, which severely compromise the coulombic efficiency (CE).^9,10^ Specifically, microscopic inhomogeneities result in localized electric-field intensifications and non-uniform Zn^2+^ concentration gradients during Zn plating/stripping.^6,8,9^ This exacerbates the local current density at the Zn anode and triggers uncontrolled 2D diffusion, leading to dendrite growth on the Zn anode/electrolyte interface.^10–12^ Consequently, developing strategies to regulate the directional transport of Zn^2+^ ions is crucial for achieving homogeneous deposition and high-performance aqueous ZIBs.^13–15^

Various strategies have been proposed to address these challenges, including electrolyte engineering,^16^ the construction of Zn deposition hosts^17^ and the design of multifactorial separators.^18^ Among them, separator modification offers an effective solution for manipulating Zn deposition kinetics by homogenizing the Zn^2+^ flux to achieve uniform Zn plating on the Zn anode.^19–22^ However, integrating small organic molecules into separators as conductive channels for regulating metal-ion transport between metal anodes and the electrolyte remains relatively unexplored.^22^ Early studies have proposed that the cation-π interaction between transition-metal cations and benzene molecules is basically an electrostatic interaction since positively charged cations interact with the negatively charged π-electron clouds of π systems.^23,24^ Naphthalene (Nt), a polycyclic aromatic hydrocarbon, features a denser π-electron cloud than that of monocyclic benzene.^24^ These characteristics originate from the intrinsic molecular structure of Nt, suggesting its potential for optimizing Zn deposition.

Designing Zn^2+^-conductive separators represents a promising strategy to promote robust Zn^2+^ transport and enable high-performance aqueous ZIBs.^25,26^ Nature serves as the ultimate source of inspiration for developing an efficient Zn^2+^ conduction strategy based on conventional glass fiber (GF) separators.^20^ Herein, we report the rational design of a biomimetic naphthalene/glass fiber separator (NtGF) by integrating Nt molecules onto GF separators. The dense π-electron cloud of Nt facilitates its interaction with Zn^2+^. Experimental results, supported by computational simulations, confirm that NtGF separators facilitate rapid Zn^2+^ transport and uniform Zn plating/stripping kinetics. By addressing the critical challenges of Zn^2+^ transport in aqueous ZIBs, this modification enables improved Zn deposition kinetics, thereby contributing to the development of high-performance aqueous ZIBs. Furthermore, the functionalized NtGF separator homogenizes the interfacial electric field and accelerates Zn^2+^ desolvation to improve Zn plating uniformity *via* the interaction between Nt molecules and Zn^2+^.^24^ Eventually, symmetric Zn//Zn cells demonstrate outstanding long-term stability over 1480 h. The assembled Zn/NtGF/NH_4_V_4_O_10_ full batteries deliver a high specific capacity of 266.5 mAh g^−1^ and maintain 91.3% capacity retention after 1000 cycles at 5 A g^−1^, showcasing the potential of biomimetic separator design for advanced aqueous ZIBs.

## 2. Experimental section

All reagents were of analytical grade and were used without further purification.

### 2.1 Synthesis of the NtGF separator *via* vacuum filtration

The calculated amount of Nt precursor was dissolved in dimethoxy ethane (DME) to prepare 0.005, 0.01 and 0.015 mol L^−1^ Nt/DME solutions, respectively. Then, 10 mL of each Nt/DME solution was deposited onto conventional glass fiber (GF) substrates by vacuum filtration. The resulting NtGF separators were then vacuum dried in the antechamber of a glovebox for 30 min at room temperature to remove residual DME and obtain the NtGF separator.

### 2.2 Preparation of NH_4_V_4_O_10_ cathode active materials

NH_4_V_4_O_10_ was synthesized according to the method previously published by our group.^27^ In brief, 1.170 g of NH_4_VO_3_ (Macklin, AR grade) was dissolved in 35 mL of deionized water with agitation at 80 °C until it was completely dissolved, forming a pale-yellow solution. Subsequently, 1.891 g of C_2_H_2_O4·2H_2_O (Macklin, AR grade) was added to the pale-yellow solution with continuous stirring for 30 min. Then, the mixed solution was transferred into a Teflon-lined autoclave and heated at 140 °C for 48 h. Subsequently, the dark-blue precipitate was obtained and washed by centrifugation with deionized water and absolute ethanol three times each. Finally, the prepared product was dried at 80 °C in a vacuum oven for 12 h to obtain the NH_4_V_4_O_10_ cathode material.

### 2.3 Electrochemical measurements

All electrochemical measurements were performed in standard CR2016 coin cells. Each cell was assembled with a Zn foil anode (10 mm diameter and 50 µm thickness), an NH_4_V_4_O_10_-based cathode, and either a pristine GF separator or an NtGF separator (19 mm diameter and 1 mm thickness), with 100 µL of 2 M aqueous ZnSO_4_ electrolyte injected. The cathode slurry was composed of NH_4_V_4_O_10_ active material, conductive carbon black and polyvinylidene fluoride (PVDF) at a mass ratio of 7 : 2 : 1, which was cast onto a stainless-steel mesh current collector. The areal loading of NH_4_V_4_O_10_ was fixed at 1.2 mg per electrode. The circular cathode electrode possessed a diameter of 10 mm and an active area of 0.7854 cm^2^, delivering a total cathode capacity of 0.1872 mAh and an areal capacity of 0.2384 mAh cm^−2^. The 50 µm-thick Zn foil anode (10 mm diameter) provided a total theoretical capacity of 2.1598 mAh, corresponding to an N/P capacity ratio of ∼11.54. The electrolyte-to-capacity (E/C) ratio was calculated to be 534.19 µL mAh^−1^ according to the applied electrolyte volume of 100 µL per cell.

With an average discharge voltage plateau of ∼1.1 V, the gravimetric energy density based on NH_4_V_4_O_10_ active material reached 171.6 Wh kg^−1^, and the areal energy density was 0.2622 mWh cm^−2^. Galvanostatic charge/discharge cycling tests were carried out on a LAND CT2001 battery test system (China) at different current densities to evaluate the rate performance and cycling stability. The electrochemical voltage window for the Zn/NtGF/NH_4_V_4_O_10_ full cells was set to 0.4–1.4 V (*vs.* Zn/Zn^2+^).

The baseline capacity was calculated from the average discharge capacity of the first five cycles after activation. The cycling tests were terminated based on the following predefined failure criteria: (i) the discharge capacity decayed to less than 80% of the baseline capacity; (ii) the coulombic efficiency was continuously lower than 95% for five successive cycles; and (iii) a severe increase in voltage polarization or a sudden voltage drop was observed, suggestive of an internal micro-short circuit caused by Zn dendrites. For Zn‖Zn symmetric cells, the cycling test was terminated when the overpotential sharply increased beyond 200 mV or continuous irregular voltage oscillations were observed, which indicated severe electrode passivation, Zn pulverization or dendrite penetration through the separator.

Cyclic voltammetry (CV), electrochemical impedance spectroscopy (EIS), Tafel polarization and chronoamperometry (CA) analyses were carried out on an electrochemical workstation (CHI660E, China). The frequency range for EIS was 10^−2^ to 10^5^ Hz. ZView software was used to fit the EIS results. The Tafel polarization measurements employed a three-electrode system, with the Zn anode as the working electrode and Zn foils as the counter and reference electrodes. The GF or NtGF separator was sandwiched between the working and counter electrodes. The CA analysis was conducted under an applied bias voltage of 150 mV.

The ionic conductivity was evaluated using stainless-steel symmetric cells and calculated according to the following equation:1
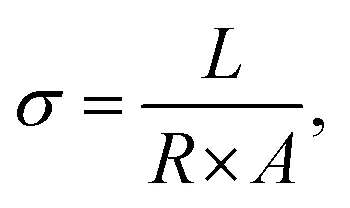
where *R* is the bulk resistance of the electrolyte, obtained from the intercept with the real-axis in the Nyquist plot; *L* is the thickness of the separator; and *A* is the electrode surface area. The Zn^2+^ transference number (*t*_Zn^2+^_) was measured in Zn//Zn symmetric cells using a chronoamperometric method with a constant overpotential of 10 mV and calculated using the Bruce–Vincent equation:2
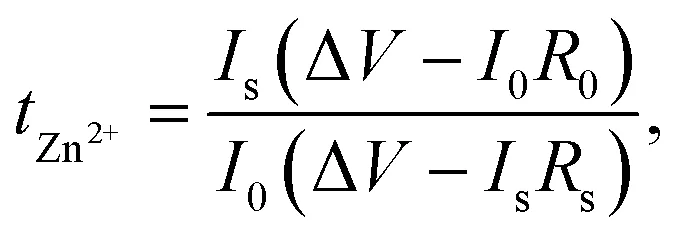
where *I*_0_ and *I*_s_ are the initial and steady-state currents, respectively, and *R*_0_ and *R*_s_ are the corresponding interfacial resistances before and after polarization, respectively, as determined by EIS.

The activation energy (*E*_a_) for Zn plating was determined from temperature-dependent impedance measurements in Zn//Zn symmetric cells. The temperature dependence of the charge-transfer resistance (*R*_ct_) was fitted using to the Arrhenius equation:3
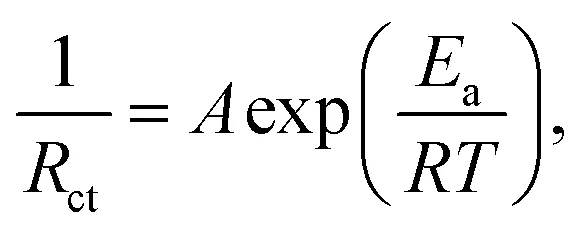
where *A* is the pre-exponential factor, *R* is the gas constant (8.314 J mol^−1^ K^−1^), and *T* is the absolute temperature.

## 3. Results and discussion

Fig. 1A schematically illustrates the mechanism governing Zn^2+^ transport across the biomimetic NtGF separator (Fig. S1). The π-conjugated ring of Nt, endowed with delocalized electrons, facilitates ultrafast Zn^2+^ migration within the NtGF separator, which is in accordance with our design strategy for selective Zn^2+^ conduction and biomimetic functionality. Fig. 1B shows the X-ray diffraction (XRD) patterns, revealing characteristic crystalline peaks of Nt located at 2*θ* = 11.96° and 24.20°, corresponding to the (001) and (002) crystallographic planes, respectively.^23^ These diffraction signatures indicate that Nt moieties are successfully assembled on the GF microstructure. The Nt exhibits a loading of 0.21 wt% (Fig. S2) with a favorable affinity for coordinating with Zn^2+^ during plating/stripping cycles. The coordination effectively regulates Zn^2+^ conduction *via* its interaction with Zn.^28,29^

**Fig. 1:**
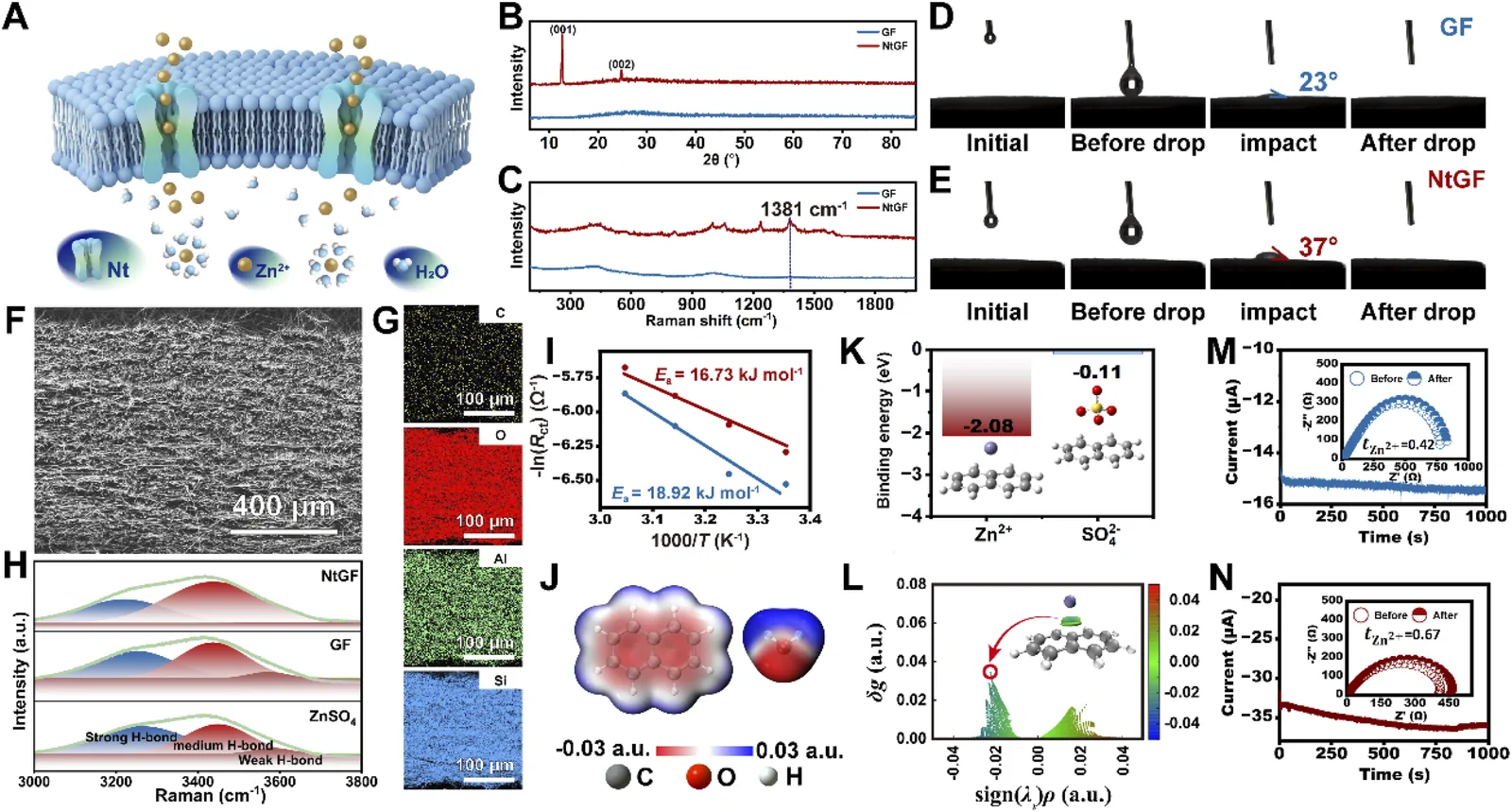
(A) Schematic of the biomimetic NtGF separator illustrating the regulatory mechanism governing Zn^2+^ ion transport in aqueous electrolytes. (B and C) XRD patterns (B) and Raman spectra (C) of GF and NtGF separators, showing their crystalline structures and chemical compositions. (D and E) Time-dependent contact angles of the GF and NtGF separators in contact with an aqueous 2 M ZnSO_4_ electrolyte, respectively. (F and G) Cross-sectional SEM image and EDS mapping images of the NtGF separator, respectively. (H) Raman spectra showing the evolution of the hydrogen-bond network. (I) Arrhenius plots and activation energies for Zn^2+^ desolvation in the presence of the GF and NtGF separators. (J) Electrostatic potential (ESP) maps of Nt and H_2_O. (K) Calculated binding energies of Zn^2+^, SO_4_^2−^ with the NtGF separator. (L) Plots of RDG *versus* sign (*λ*_2_) *ρ*. (M and N) Potentiostatic polarization curves and EIS spectra (inset) of Zn//Zn symmetric batteries using the GF and NtGF separators, respectively.

Raman spectroscopy plots further validates the successful integration of Nt onto GF substrates through the characteristic peak at 1381 cm^−1^ (Fig. 1C), which corresponds to Nt vibrations.^24^ Time-dependent wettability analysis demonstrates that pristine GF displays a contact angle of 23° with 2 M ZnSO_4_ electrolyte immediately upon impact (Fig. 1D and E), while the NtGF separator exhibits a contact angle of 37° (Fig. 1E). The NtGF separator exhibits a substantially higher electrolyte uptake than the pristine GF separator (Fig. S3 and S4). Despite the adsorption induced by the interaction between Nt molecules and Zn^2+^, as well as the lower porosity and specific surface area of the Nt separator (Fig. S5–S7),^30,31^ this counterintuitive enhancement can be attributed to the superior zincophilicity of the Nt moiety, which promotes electrolyte affinity beyond the textural properties of the separator, thereby mitigating the direct interaction between the NtGF separator interface and detrimental free water molecules in the ZnSO_4_ electrolyte. Along with the enhanced mechanical properties of the NtGF separator, as evidenced by tensile and puncture tests (Fig. S8 and S9), the physical barrier against Zn dendrite penetration is expected to be reinforced.

The surface and cross-sectional scanning electron microscopy (SEM) images, coupled with energy-dispersive X-ray spectroscopy (EDS) mapping, exhibit a uniform distribution of C, O, Al and Si elements throughout the NtGF separator (Fig. 1F, G, S10 and S11). Transmission electron microscopy (TEM) images reveal a uniform Nt coating with a thickness of around 2 nm (Fig. S12 and S13). Raman spectroscopy provides experimental evidence for the regulatory effect of the separator on the hydrogen-bond network of water (Fig. 1H). Compared to the electrolyte-soaked GF separator, the NtGF separator exhibited a decreased proportion of both strong and weak hydrogen bonds, accompanied by an increased proportion of medium strength hydrogen bonds. This indicates that the NtGF separator competes with H_2_O molecules for hydrogen bonding, thereby disrupting the bulk hydrogen-bond network of the electrolyte. Consequently, this simultaneously enhances Zn^2+^ desolvation kinetics and suppresses water-induced parasitic reactions.^16^ Electrochemical impedance spectroscopy (EIS) further demonstrates that the NtGF separator promotes Zn^2+^ desolvation, yielding a reduced charge-transfer activation energy of 16.73 kJ mol^−1^, compared with that of the GF separator (18.92 kJ mol^−1^) (Fig. 1I and S15–S23). Molecular electrostatic potential (ESP) calculations were performed on isolated naphthalene (Nt) and H_2_O systems (Fig. 1J). In the ESP color scheme, red regions represent electron-rich zones with negative electrostatic potential (−0.03 a.u.), while blue regions denote electron-deficient domains with positive electrostatic potential (0.03 a.u.). For the aromatic Nt molecule, high electron density is concentrated on the carbon skeleton within its π-conjugated ring, which facilitates Zn^2+^ transport.^32^ Density functional theory (DFT) computations were further conducted to investigate the interactions of Nt with Zn^2+^ and SO_4_^2−^ (Fig. 1K). The binding energy of Nt with Zn^2+^ was calculated to be −2.08 eV, significantly more negative than that with SO_4_^2−^ (−0.11 eV), indicating that Zn^2+^ is preferentially captured by the Nt molecule through its high electron density and highly electronegative extended π-conjugated ring. This strong interaction is responsible for the enhanced Zn^2+^ transport kinetics.^32^ The DFT calculation results further validate the ability of the separator to mediate Zn^2+^ desolvation kinetics. The reduced density gradient (RDG) scatter plot reveals that the prominent green peaks located at sign (*λ*_2_)*ρ* values of (−0.02, 0.035) are attributable to the extensive green isosurface regions (Fig. S14), which constitute the dominant van der Waals component of the Zn^2+^–π interaction. A distinct upward cyan/teal spike with an intensity of 0.035 represents the strength of the attractive interactions (Fig. 1L). This dominant attractive interaction is characterized by a delocalized Zn^2+^–π interaction across the Nt molecule, reflecting the attractive interaction between the Zn ion and the ring centroid (inset of Fig. 1L).^30^ Moreover, the NtGF separator exhibits a Zn^2+^ transference number (*t*_Zn^2+^_) of 0.67 (Fig. S26 and S27), which is higher than that of the GF separator (0.42) (Fig. S24 and S25). This higher *t*_Zn^2+^_ value facilitates Zn^2+^ transport, thereby efficiently suppressing the growth of Zn dendrites (Fig. 1M and N).

Ionic conductivity measurements for the NtGF separator with the optimal Nt concentration of 0.01 M yielded a value of 7.21 mS cm^−1^ (Fig. S28), compared with 1.78 mS cm^−1^ for the bare GF separator (Fig. 2A and B). This enhancement is ascribed to the delocalized π-electrons of the aromatic rings, which facilitate rapid Zn^2+^ migration. Such improved transport kinetics may consequently influence the interfacial electrochemical activity and corrosion behavior. In general, high electrochemical activity at dendrite tips typically leads to decreased corrosion resistance. Zn electrodes coupled with GF and NtGF separators served as the working electrode and counter/reference electrodes, respectively, for Tafel polarization analysis.^33,34^ Tafel curves demonstrate that the NtGF separator yields a significantly lower corrosion current density of 0.05 mA cm^−2^, compared with 2.63 mA cm^−2^ for the GF separator (Fig. 2C), indicating superior corrosion resistance of Zn electrodes with the modified interface.^35,36^ Furthermore, the Zn growth dynamics were investigated by chronoamperometry (CA) (Fig. 2D). With the GF separator, the current density continuously increased up to 400 s, indicative of rampant and uncontrolled 2D lateral Zn^2+^ diffusion. Conversely, the NtGF separator exhibited relatively constrained lateral growth within 20 s, followed by prolonged and stable 3D vertical Zn plating with a lower steady-state current response. These results suggest that the application of an NtGF separator effectively inhibits dendrite nucleation on the Zn surface and mitigates tip effects, thereby promoting uniform Zn deposition. The regulatory effect was further confirmed *via* SEM morphology analysis of cycled Zn electrodes (Fig. S29). After plating at 2 mA cm^−2^ with an areal capacity of 2 mAh cm^−2^, the Zn electrode coupled with the NtGF separator showed a smooth and flat morphology, whereas the electrode using the GF separator exhibited mossy growth with abundant parasitic deposits.

**Fig. 2:**
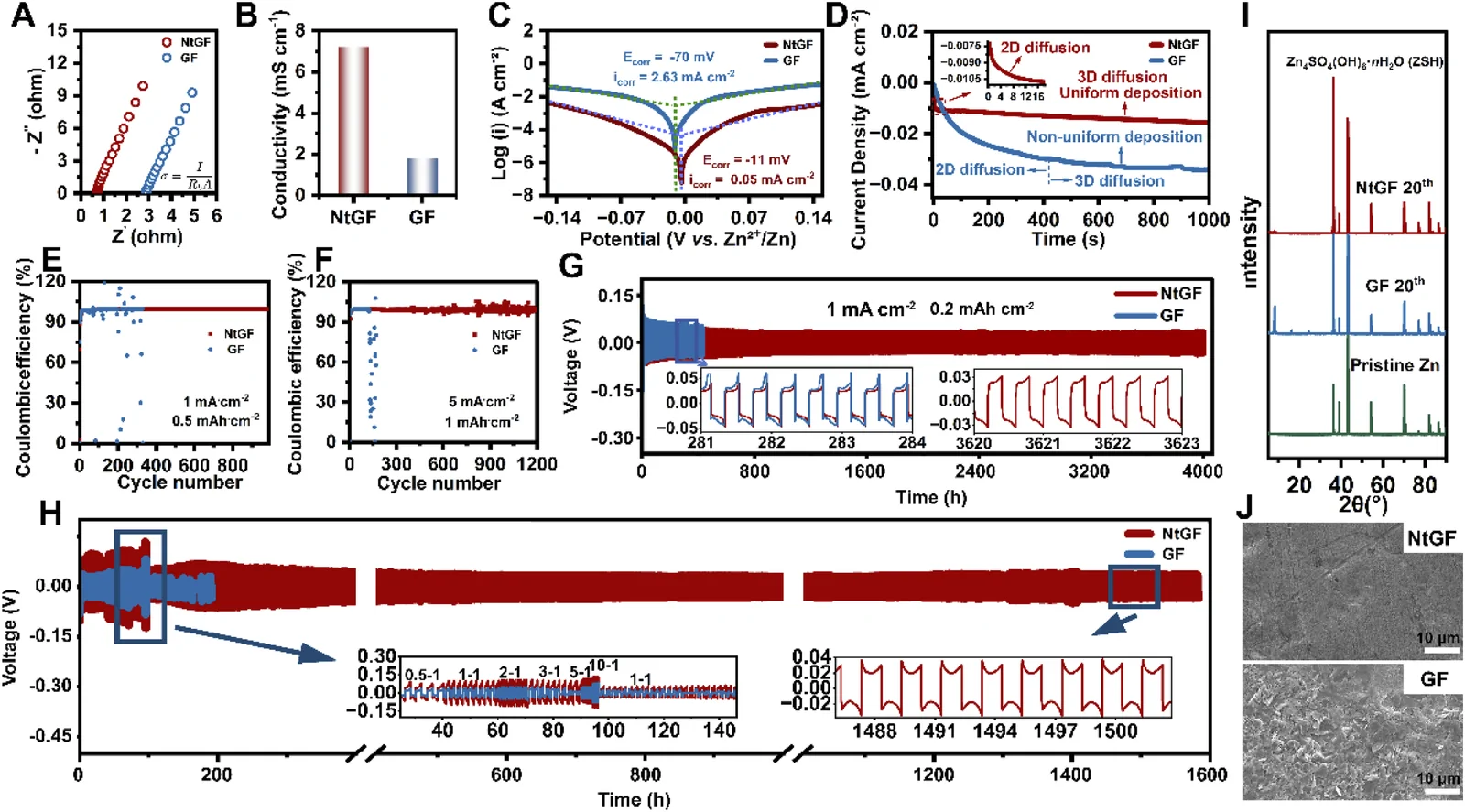
(A and B) Ionic conductivities of GF and NtGF separators. (C) Tafel plots for the GF and NtGF separators at a scan rate of 5 mV s^−1^. (D) Chronoamperogram (CA) curves of Zn//Zn symmetric cells with the GF and NtGF separators, measured at an overpotential of −150 mV. Coulombic efficiency (CE) of the Zn//Cu cells at a current density of 1 mA cm^−2^ and an areal capacity of 0.5 mAh cm^−2^ (E), and at 5 mA cm^−2^ and an areal capacity of 1 mAh cm^−2^ (F). (G) Long-term cycling stability of Zn//Zn symmetric cells using the GF and NtGF separators at a current density of 1 mA cm-2. (H) Rate performance of Zn//Zn symmetric cells using the GF and NtGF separators at current densities ranging from 0.5 to 10 mA cm^−2^. XRD patterns (I) and SEM images (J) of Zn anodes after 20 cycles with the GF and NtGF separators.

Zn//Cu asymmetric cells and Zn//Zn symmetric cells were fabricated to evaluate reversible Zn plating/stripping using either pristine GF or NtGF separators. The uniform distribution of Nt moieties on the separator surface provides abundant zincophilic active sites while effectively regulating Zn^2+^ flux (Fig. 1B and S11A).^37^ The NtGF-based Zn//Cu asymmetric cell demonstrated highly stable Zn plating/stripping at 0.5 mA cm^−2^ and 0.25 mAh cm^−2^ (Fig. S30). Notably, it also delivered a steady coulombic efficiency of 99.9% for up to 990 cycles at 1 mA cm^−2^ with an areal capacity of 0.5 mAh cm^−2^, markedly surpassing the performance of the GF separator (Fig. 2E, S31A and B). Even at a higher current density of 5 mA cm^−2^ (Fig. 2F, S31C and D), the Zn//Cu cells with the NtGF separator maintained a coulombic efficiency of 99.2% and sustained cycling for 1200 cycles, demonstrating enhanced Zn deposition stability compared with that of the GF separator. These metrics significantly surpass those of recently reported advanced metal-anode stabilization strategies (Table S1),^38^ highlighting the distinct potential of the NtGF architecture for application to Zn metal electrodes.

Furthermore, tests of symmetric Zn//Zn cells confirmed exceptional long-term stability (Fig. 2G and H). The symmetric cells equipped with NtGF separators maintained nearly flat voltage plateaus for over 4000 h at 1 mA cm^−2^ (Fig. 2G). During the rate performance evaluation, the Zn//Zn symmetric cell maintained stable and reversible cycling under different current densities (Fig. S32). Specifically, the Zn//Zn symmetric cell delivered stable cycling for over 1480 h at a current density of 1 mA cm^−2^ after rate capability tests ranging from 0.5 mA cm^−2^ to 10 mA cm^−2^. In contrast, GF-based controls displayed moderate voltage polarization after only 80 h and suffered from internal short-circuiting within tens of hours (inset of Fig. 2H). Even at elevated current densities of 5 mA cm^−2^ and 10 mA cm^−2^, the cells with the NtGF separator maintained stable voltage profiles, whereas GF-based controls failed prematurely (inset of Fig. 2H). To evaluate the structural stability of Nt during Zn plating/stripping, Fourier-transform infrared (FTIR) spectroscopy analysis was performed on the recovered separators. Fig. S33 reveals that the characteristic absorption bands of Nt, notably the aromatic C–H stretching vibration at 3048 cm^−1^,^39^ were well-preserved after 250 cycles. Additionally, UV-vis spectroscopy of ZnSO_4_ electrolytes soaked with the NtGF separators and collected after different numbers of Zn plating/stripping cycles (Fig. S34) showed no detectable absorption peaks for Nt at 220 nm. This confirms that Nt does not dissolve or leach into the electrolyte over the course of cycling. The long-term cycling performance and extended cycle life greatly surpass those of reported Zn metal electrodes stabilized by various separator-modification strategies (Table S1). After 20 plating cycles, XRD analysis showed the formation of Zn_4_SO_4_(OH)_6_·*n*H_2_O (ZSH) byproducts on the GF-based Zn electrode, which were absent in the presence of the NtGF separator, indicating effective suppression of parasitic reactions (Fig. 2I). The GF-based electrode exhibited moss-like dendrites, whereas the NtGF-based electrode displayed a smooth and dendrite-free surface (Fig. 2J). These results highlight the essential role of the NtGF separator in homogenizing Zn^2+^ flux and regulating Zn deposition.

Experimental observation of Zn nucleation at the 10th and 50th cycles (Fig. 3A, B and S35) revealed that the NtGF separator ensures uniform Zn deposition and suppresses dendrite growth, while the GF separator suffered from severe dendrite growth and byproduct formation. This confirms the role of the NtGF separator in regulating Zn^2+^ transport and deposition kinetics (Fig. 3C), in line with the ESP/DFT analyses (Fig. 1I and J). To gain deeper insight into interfacial Zn^2+^ regulation, finite element method (FEM) simulations using COMSOL Multiphysics were employed to model the local electric fields (Fig. 3D and E) and Zn^2+^ concentration fields (Fig. 3F and G) at the Zn electrode/electrolyte interface.^38,40^ The simulation domain was defined with in-plane dimensions of 50 × 50 µm^2^. Fig. 3D to G show the simulated vertical electric-field distributions and Zn^2+^ diffusion fluxes for both the bare GF and NtGF architectures. For the pristine GF separator, strong local polarization induced higher local current densities near interfacial imperfections, promoting uneven ion accumulation (Fig. 3D, S36 and S38A). Conversely, in the presence of Nt molecules in the NtGF separator, electrostatic interactions between Nt moieties and Zn^2+^ effectively redistributed the vertical ion flux towards lateral pathways. The π-rich system provides abundant and homogeneous nucleation sites, enabling Zn to preferentially nucleate uniformly rather than forming isolated protrusions. This effect homogenizes the interfacial electric field, thereby eliminating localized electric-field hotspots and suppressing charge accumulation at dendritic tips, which commonly initiates dendrite nucleation in aqueous electrolytes (Fig. 3E, S37 and S37B).^33,35^

**Fig. 3:**
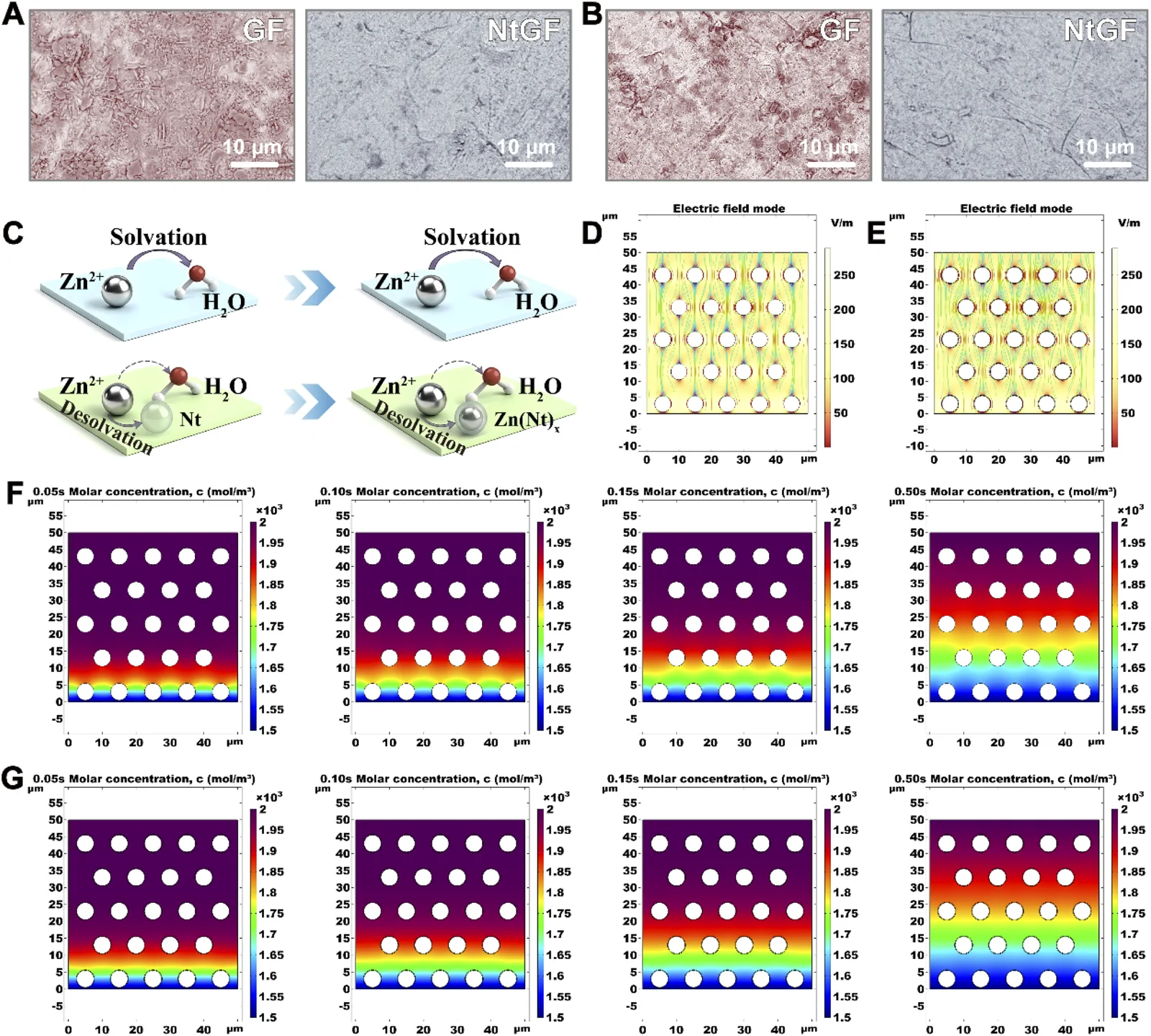
(A and B) SEM images of Zn plating on Zn electrodes from Zn//Zn symmetric cells at a current density of 1 mA cm^−2^ and an areal capacity of 1 mAh cm^−2^ after 10th (A) and 50th (B) plating/stripping cycles. (C) Schematic of the Nt-related mechanisms of the NtGF separator for regulating Zn^2+^ transport during Zn plating/stripping. (D and E) Simulated electric fields for the GF separator (D) and NtGF separator (E). (F and G) Simulated concentration fields after a constant diffusion time of 0.5 s for the GF separator (F) and NtGF separator (G).

Regarding the simulated Zn^2+^ concentration field, finite element method analysis indicates that Zn^2+^ diffusion, driven by the electric field, occurs from 0.05 s to 0.1 s in the GF separator (Fig. 3F and S39A). During this period, Zn^2+^ rapidly accumulates at surface protrusions, with high local concentrations, while adjacent regions remain relatively depleted. With the diffusion time further increased from 0.15 s to 0.5 s, the Zn^2+^ accumulation intensifies significantly near the protrusions (Fig. 3F). Conversely, in the NtGF separator functionalized with electron-rich Nt moieties, Zn^2+^ migration is homogenized from 0.05 s to 0.1 s and progressively aligns along the diffusion *Y*-axis, rapidly achieving a steady state at 0.5 s (Fig. 3G and S39B).^41^ Consequently, the uniformly distributed Nt molecules effectively guide the redistribution of Zn^2+^, establishing a homogeneous concentration environment conducive to stable Zn deposition kinetics.

The novel NtGF separator, endowed with intrinsic zincophilic properties, exhibits exceptional efficacy in suppressing Zn dendrite growth and parasitic byproduct formation through the synergistic promotion of Zn^2+^ desolvation kinetics and enhanced ionic conductivity. Fig. 4 presents the comprehensive electrochemical evaluation of the Zn/NtGF/NH_4_V_4_O_10_ full batteries to verify their practical applicability. Fig. 4A demonstrates the superior rate capability of the Zn/NtGF/NH_4_V_4_O_10_ full cells, delivering high specific capacities ranging from 481.5 mAh g^−1^ at 0.2 A g^−1^ to 219.3 mAh g^−1^ at a high current density of 10 A g^−1^. In stark contrast, the Zn/GF/NH_4_V_4_O_10_ full batteries exhibited significantly diminished performance, retaining only 347.4 mAh g^−1^ at 0.2 A g^−1^ and decreasing to 148.7 mAh g^−1^ at 10 A g^−1^. The marked improvement is primarily attributed to the rapid Zn^2+^ transport facilitated by the Nt-derived π-conjugated carbon rings enriched with π-electron density within the NtGF separator architecture, which optimize charge-transfer pathways.

**Fig. 4:**
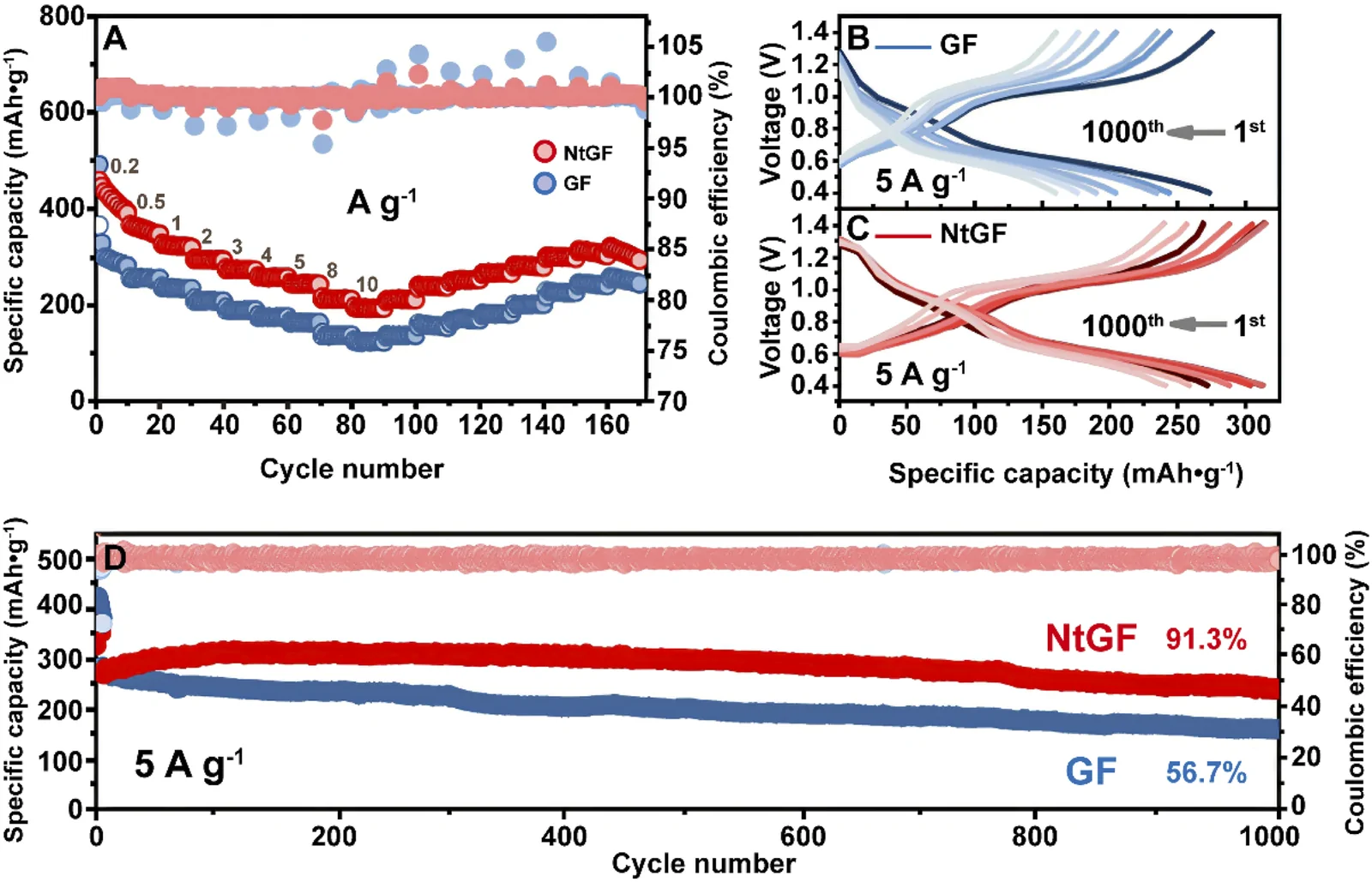
Electrochemical performance of the Zn/GF/NH_4_V_4_O_10_ and Zn/NtGF/NH_4_V_4_O_10_ batteries. (A) Rate performance of the Zn/GF/NH_4_V_4_O_10_ and Zn/NtGF/NH_4_V_4_O_10_ batteries at current densities ranging from 0.2 A g^−1^ to 10 A g^−1^. (B and C) Selected charge–discharge curves of the Zn/GF/NH_4_V_4_O_10_ and Zn/NtGF/NH_4_V_4_O_10_ batteries. (D) Long-term cycling stability of Zn/GF/NH_4_V_4_O_10_ and Zn/NtGF/NH_4_V_4_O_10_ batteries.

Long-term electrochemical stability was investigated to evaluate the performance of the NtGF separator in the Zn/NtGF/NH_4_V_4_O_10_ full batteries (Fig. S40 and 4B–D). The full batteries were initially activated at a low current density of 0.2 A g^−1^ for 5 cycles (Fig. 4D). The NtGF separator-based full cell achieved an initial discharge capacity of 291.9 mAh g^−1^ at 5 A g^−1^ and maintained a discharge capacity of 266.5 mAh g^−1^ after 1000 cycles with a retention rate of 91.3% and an average CE of 99.9%, significantly outperforming previously reported systems (Table S2). In comparison, the GF separator-based full cell displayed poor durability. Although initially reaching a maximum specific capacity of 288.8 mAh g^−1^ during the first cycle, the full cell retained only a specific capacity of 162.8 mAh g^−1^, with only 56.7% retention after just 1000 cycles under identical testing conditions, and exhibited inconsistent performance throughout cycling. The exceptional electrochemical metrics of the Zn/NtGF/NH_4_V_4_O_10_ full batteries confirm the robust interfacial Zn^2+^ modulation and transport enabled by the NtGF separator, thereby enhancing Zn anode stability and highlighting its potential for prolonged operation in aqueous ZIBs.

## 4. Conclusion

In this work, a biomimetic naphthalene/glass fiber separator (NtGF) was engineered to regulate Zn^2+^ flux and accelerate Zn^2+^ transport during plating/stripping processes for high-performance aqueous zinc-ion batteries (ZIBs). The NtGF biomimetic separator exhibits inherent zincophilicity, which is attributed to π-electron-cloud-induced Zn^2+^ adsorption. The engineered architecture fosters a synergistic effect that homogenizes both electric-field distributions and Zn^2+^ concentration gradients, effectively suppressing dendrite growth and inhibiting parasitic byproduct formation. Consequently, Zn//Zn symmetric cells with the NtGF separator maintained flat voltage plateaus for over 1480 h. The aqueous Zn/NtGF/NH_4_V_4_O_10_ full batteries retained 91.3% of their initial capacity at a current density of 5 A g^−1^. Ultimately, the biomimetic strategy leverages a molecularly engineered separator architecture, demonstrating significant potential for long-life aqueous ZIBs and sustainable grid-scale energy storage applications.

## Author contributions

Guanqian Huang: writing – original draft, methodology, formal analysis, data curation, conceptualization. Jinxiong Liang: computerization, methodology, formal analysis, data curation. Zhanpeng Yang: investigation, methodology, visualization. Yingfei Wu: conceptualization, formal analysis, data curation. Haijun Peng: writing – original draft, resources, project administration, methodology.

## Conflicts of interest

There are no conflicts to declare.

## Supplementary Material

RA-016-D6RA07297F-s001

## Data Availability

The datasets generated and/or analyzed during the current study are available from the corresponding author upon reasonable request. Supplementary information (SI): additional experimental details, materials characterization data, and supplementary electrochemical results. See DOI: https://doi.org/10.1039/d6ra07297f.
