# Supplementary material for: Biomimetic separator design through naphthalene/glass fiber integration to achieve dendrite-growth-suppressed Zn anodes for high-performance aqueous zinc-ion batteries
Source: RSC Adv. 2026 Sep 11;16(44):54742–50. doi: 10.1039/d6ra07297f (PMC13568915; doi:10.1039/d6ra07297f)
Supplement: Supplementary file 1 [file RA-016-D6RA07297F-s001.pdf]

Supplementary Information (SI) for RSC Advances.
This journal is © The Royal Society of Chemistry 2026

*Supporting Information for*

# Biomimetic separator design through naphthalene/glass fiber integration to achieve dendrite growth suppressed Zn anode for high performance aqueous zinc-ion batteries

Guanqian Huang[a,1], Jinxiong Liang [a,1*], Zhanpeng Yang [a], Yingfei Wu[a], Haijun Peng[*1]

[a]School of Intelligent Manufacturing, Guangdong University of Science and Technology, Dongguan, 523083, Guangdong, China. E-mail: 2429771484@qq.com; Penghj1989@163.com.

**Materials characterization:** Raman spectroscopy was performed using a microscopic confocal Raman spectrometer (inVia, Renishaw Ltd.), with spectra acquired across the spectral window of 100-4000 $cm^{-1}$. Fourier transform infrared (FTIR) spectra were collected on an FTIR spectrometer (Thermo, Nicolet iS5). Specific surface area and porosity information were analyzed on the Micromeritics, ASAP2020PLUS instrument. Instantaneous contact angles of the electrolyte were measured by a surface tension tester (KRUSS, DSA100). Thermogravimetric analysis (TGA) was performed using a HITACHI STA200 simultaneous thermal analyzer. UV-vis absorption spectra were recorded on a Shimadzu UV-3600i Plus spectrophotometer. Transmission electron microscopy (TEM) images were acquired on a Thermo Scientific Talos F200i transmission electron microscope. The morphological characteristics of both pristine Zn anodes and plated samples were observed utilizing scanning electron microscope (SEM, MIRA4 TESCAN, 20 kV) and optical microscope (LW750LJT). Energy-dispersive X-ray spectroscopy (EDS) was performed using a Bruker XFlash 6 detector. Crystalline phase identification was conducted *via* X-ray diffraction (XRD) using a Rigaku Mini Flex 600 diffractometer equipped with Cu Kα radiation (λ = 1.5418 Å). Tensile tests were performed using an electronic universal testing machine (MTS, model E45.105, accuracy class 0.5). The electrolyte uptake was calculated according to the following equation:

$$uptake = \frac{W_a - W_b}{W_b} \times 100\% \tag{S1}$$

Where $W_b$ and $W_a$ represent the weights before and after electrolyte immersion,

respectively. The separator porosity was tested using n-butanol as solvent, according to the following equation:

$$porosity = \frac{W_a - W_b}{\rho V} \times 100\% \quad \text{(S2)}$$

Where $W_b$ and $W_a$ represent the weights before and after soaking in the solvent, respectively. $\rho$ and $V$ represent the density of the solvent and the apparent volume of the separator.

**Theoretical Calculation**: DFT calculations were carried out using the Gaussian 16 software package.[1] The geometry optimizations were performed at the B3LYP-D3(BJ)/def2-TZVP level of theory basis set for C, H, O, and S atoms, with the SMD implicit solvation model using water as the solvent and the SDD basis set for the Zn atom.[2-7] The independent gradient model based on Hirshfeld partition (IGMH)] analysis was performed with the Multiwfn program.[8,9] The Visual Molecular Dynamics (VMD) program was used to plot the color-filled isosurface graphs, with the IGMH isosurface plotted at an isovalue of 0.01 a.u. The corresponding scatter map of sign($\lambda_2$)$\rho$ versus $\delta g$ was plotted using Gnuplot.[10] Single-point energy calculations were conducted at the B3LYP level employing the 6-311G(d,p) basis set for C, H, O, and S atoms, while the SDD basis set was retained for Zn. The binding energy ($\Delta E_{BE}$) was calculated as $\Delta E_{BE} = E_{products} - E_{reactants}$, where $E_{products}$ represents the total energy of the cocrystal system and $E_{reactants}$ denotes the sum of the energies of the individual components.

***COMSOL Multiphysical Field Simulation***: COMSOL Multiphysics 5.4 software was employed to conduct multi-physical field simulation of the electrodeposition process on zinc surface in GF and NtGF separators. The electroplating mode, diffusion and migration of the entire electrolyte and ion transport process were based on the Nernst

Planck formula. The detailed parameters are presented in Table S3 and S4. Based on the simulation results, the distribution of electric field, current, and $Zn^{2+}$ flux in GF and NtGF separators electrolytes during the electrodeposition process can be investigated.

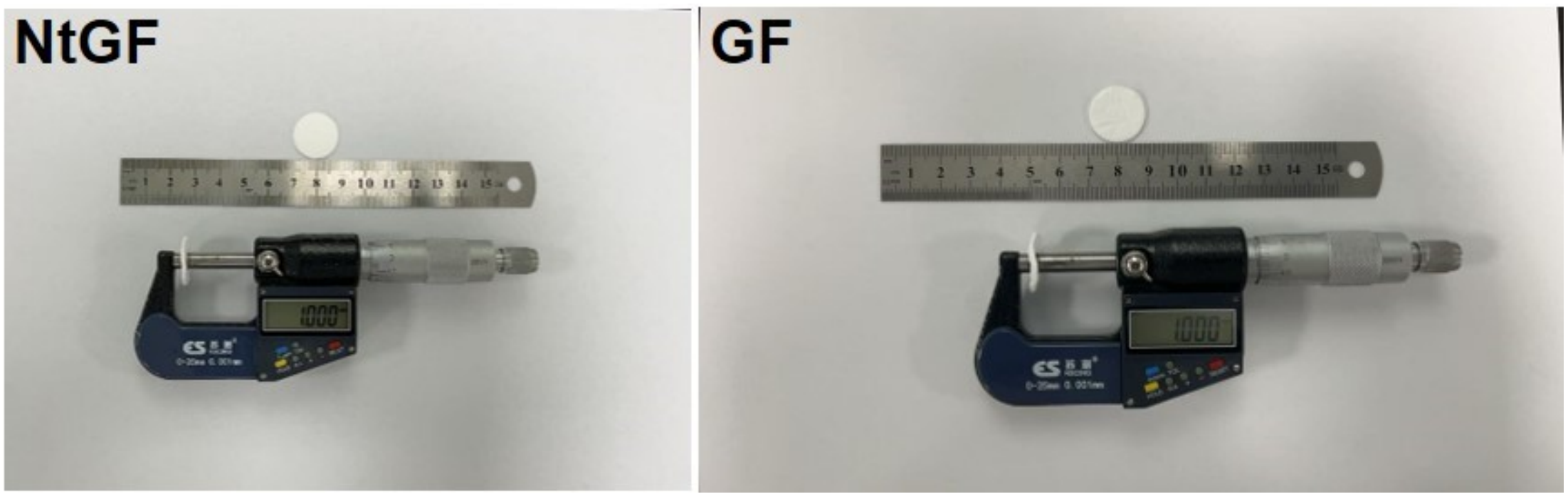


Figure S1. Optical photograph of NtGF separator (Left) and GF separator (Right).

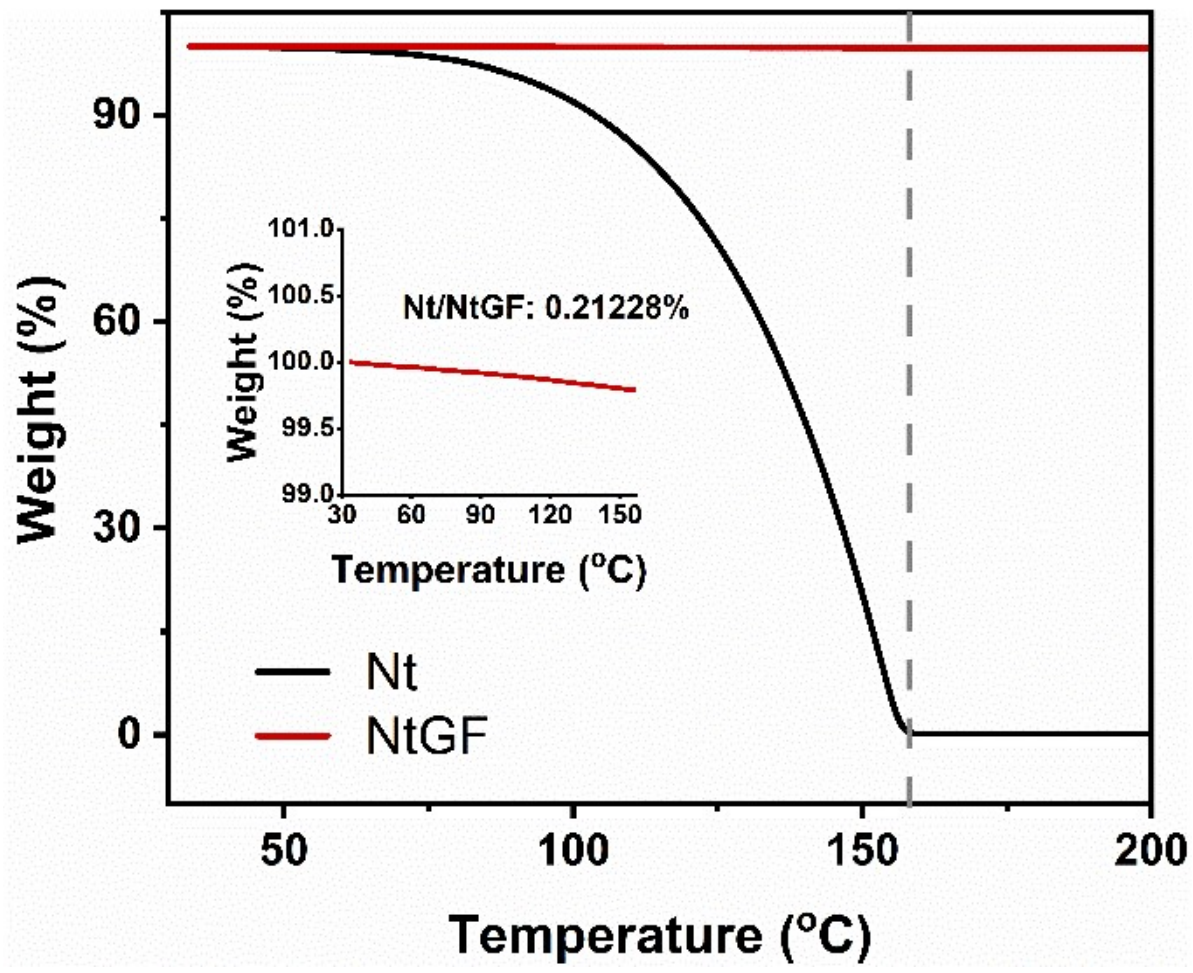


Figure S2. TGA curves of Nt and NtGF separator under $N_2$ atmosphere at a heating rate of 10 °C $min^{-1}$.

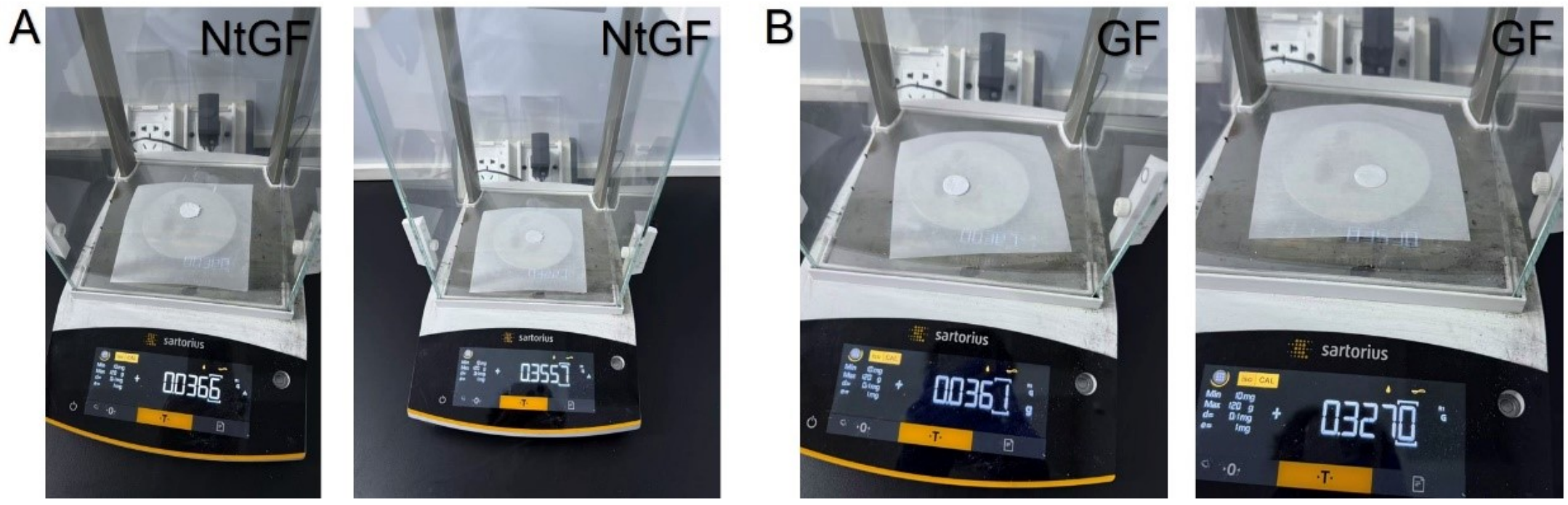


Figure S3. The electrolyte uptake test of (A) NtGF separator and (B) GF separator.

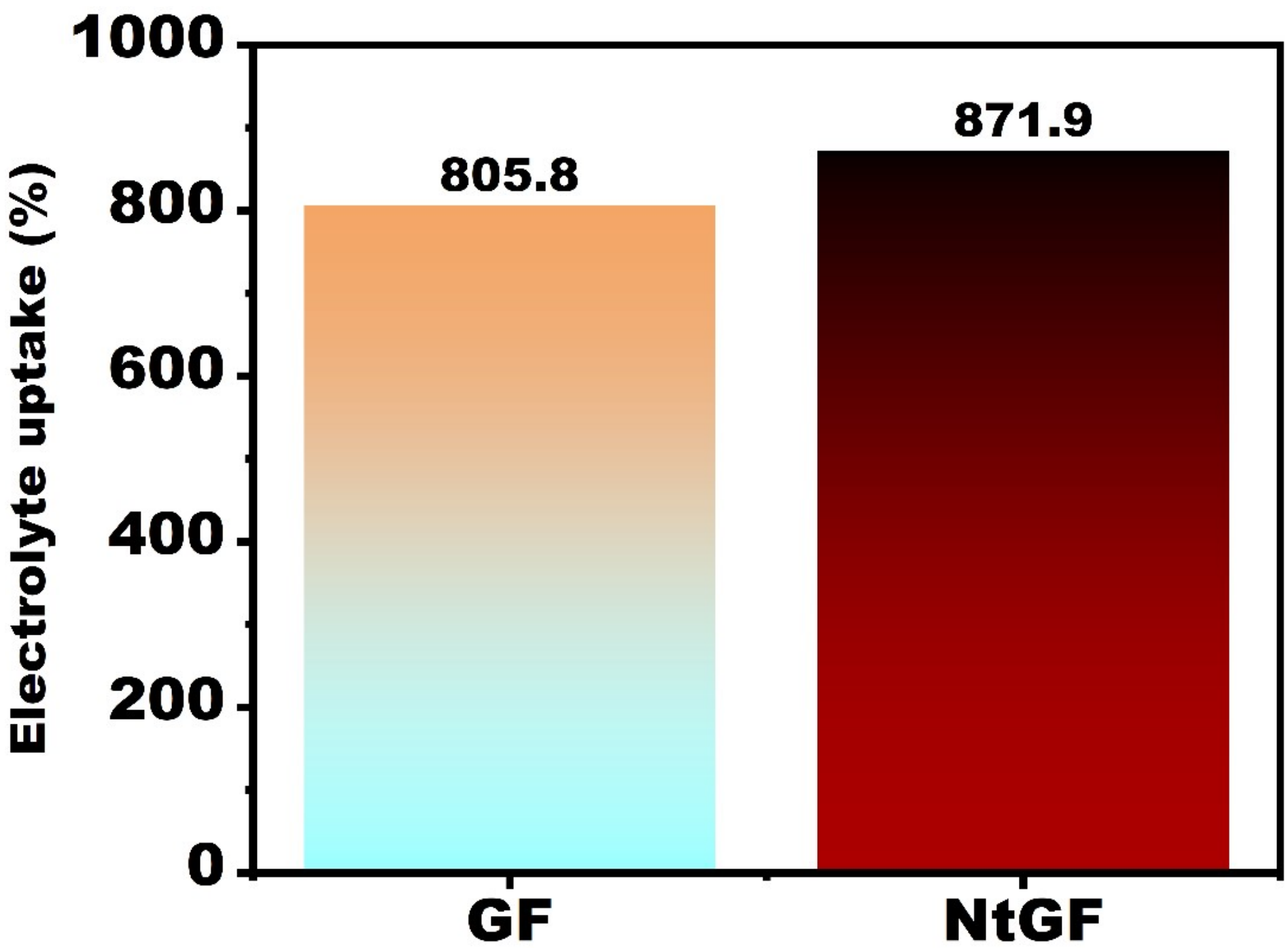


Figure S4. The electrolyte uptake of (A) NtGF separator and (B) GF separator.

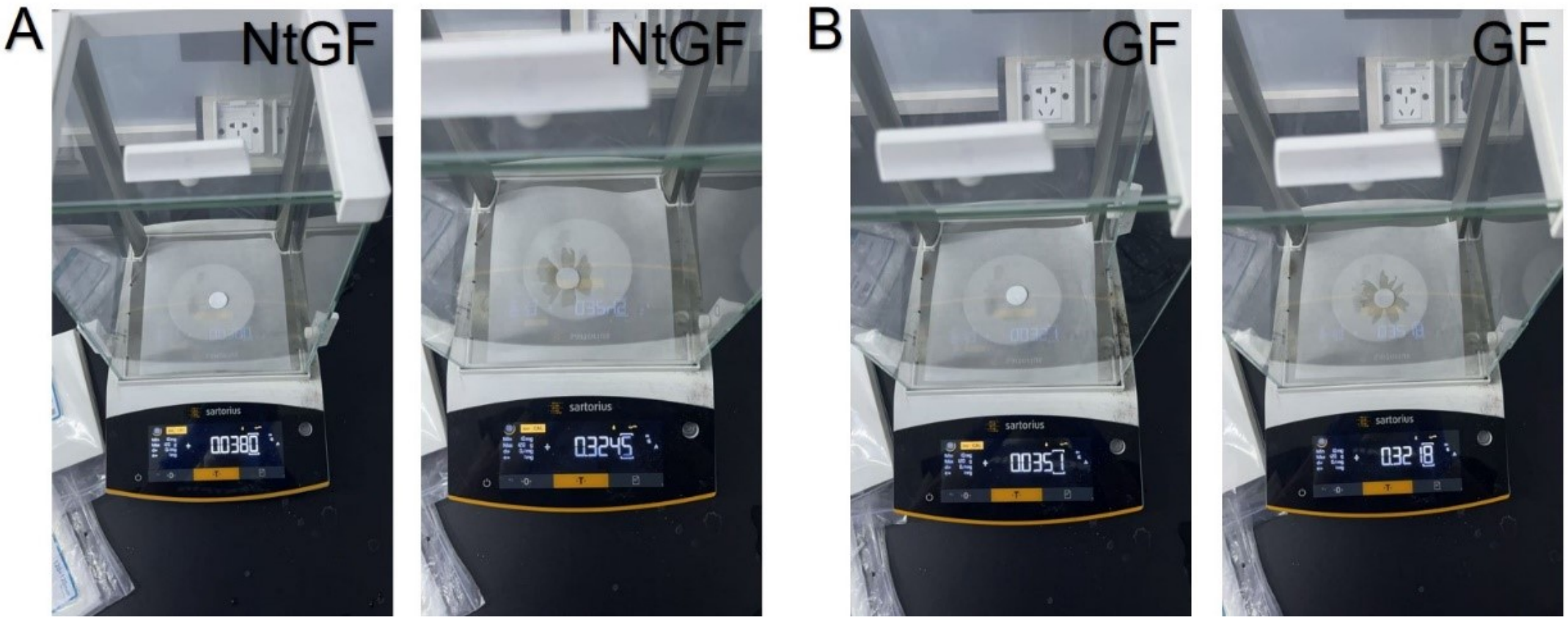


Figure S5. The porosity test of (A) NtGF separator and (B) GF separator.

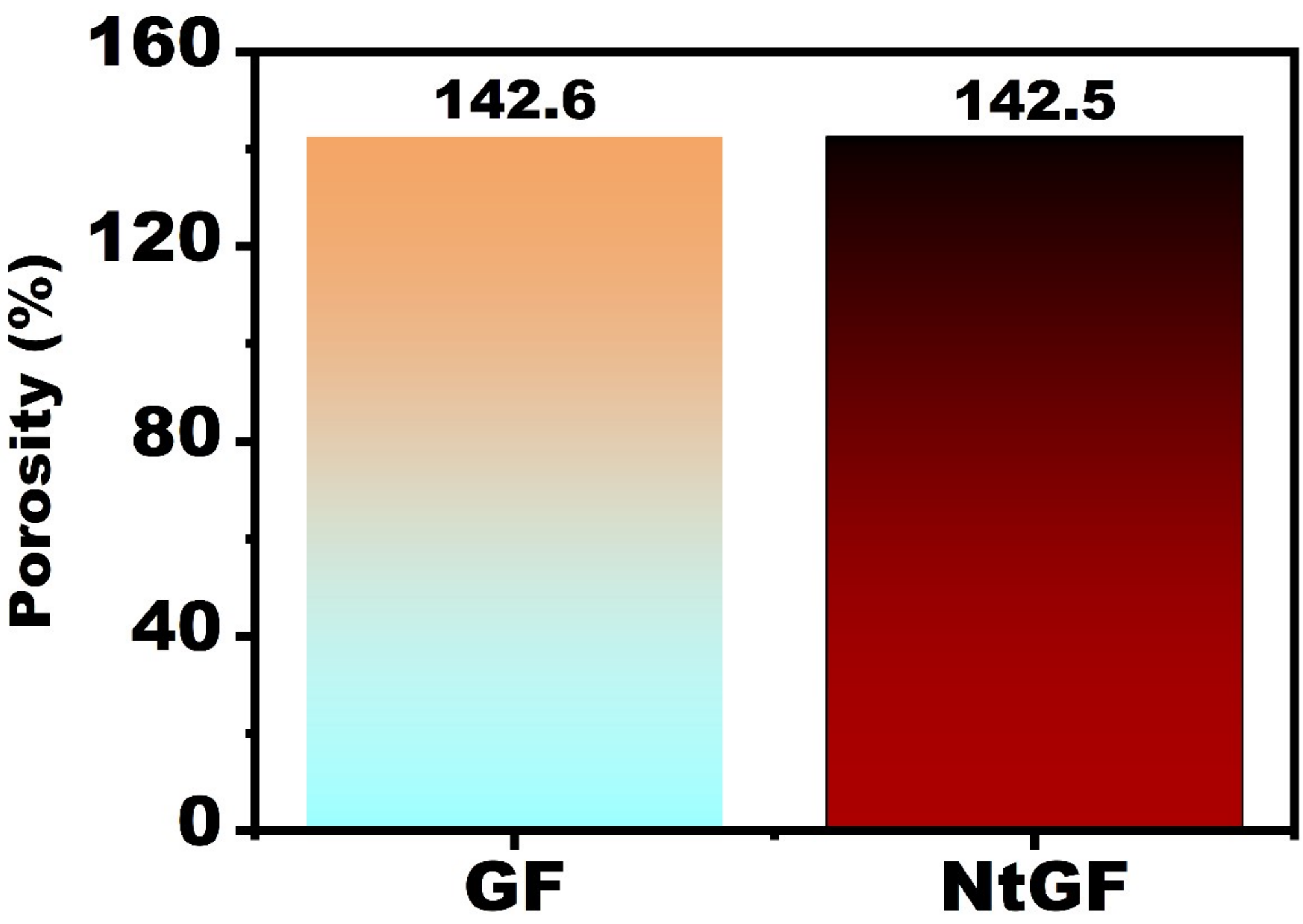


Figure S6. The porosity of (A) NtGF separator and (B) GF separator.

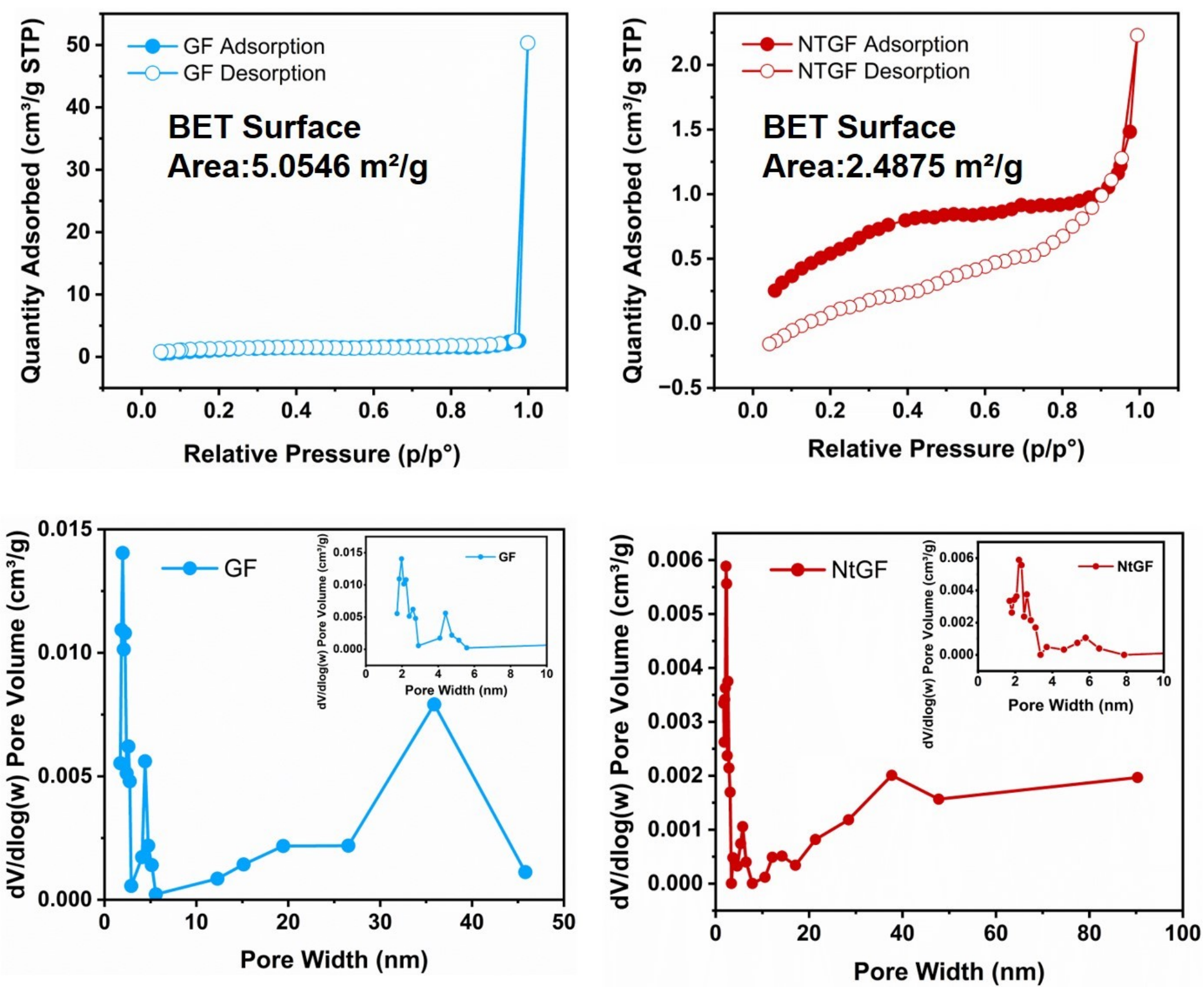


Figure S7. BET specific surface area and pore size distribution analysis of the GF and NtGF separators.

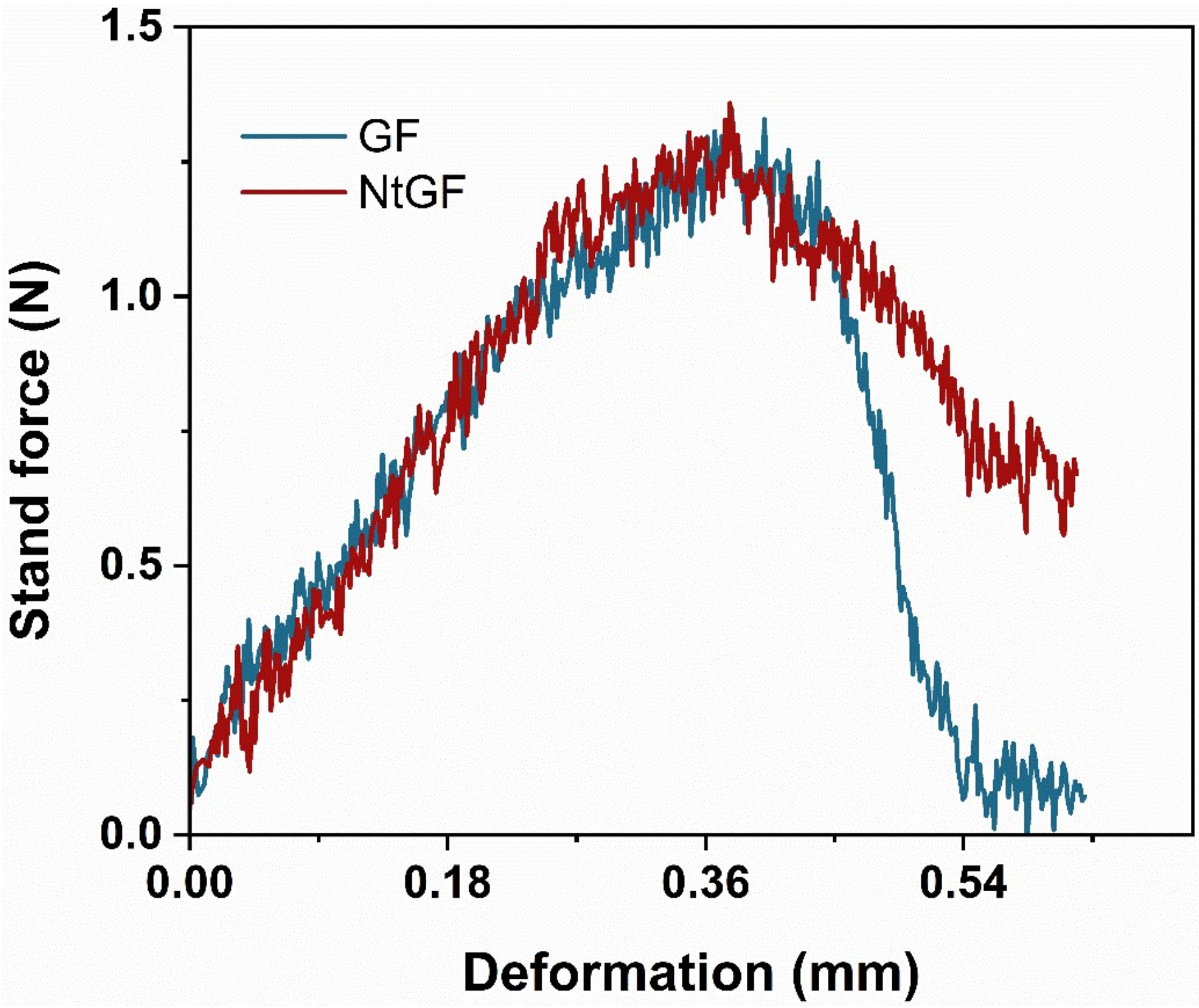


Figure S8. puncture resistance tests of the GF and NtGF separators.

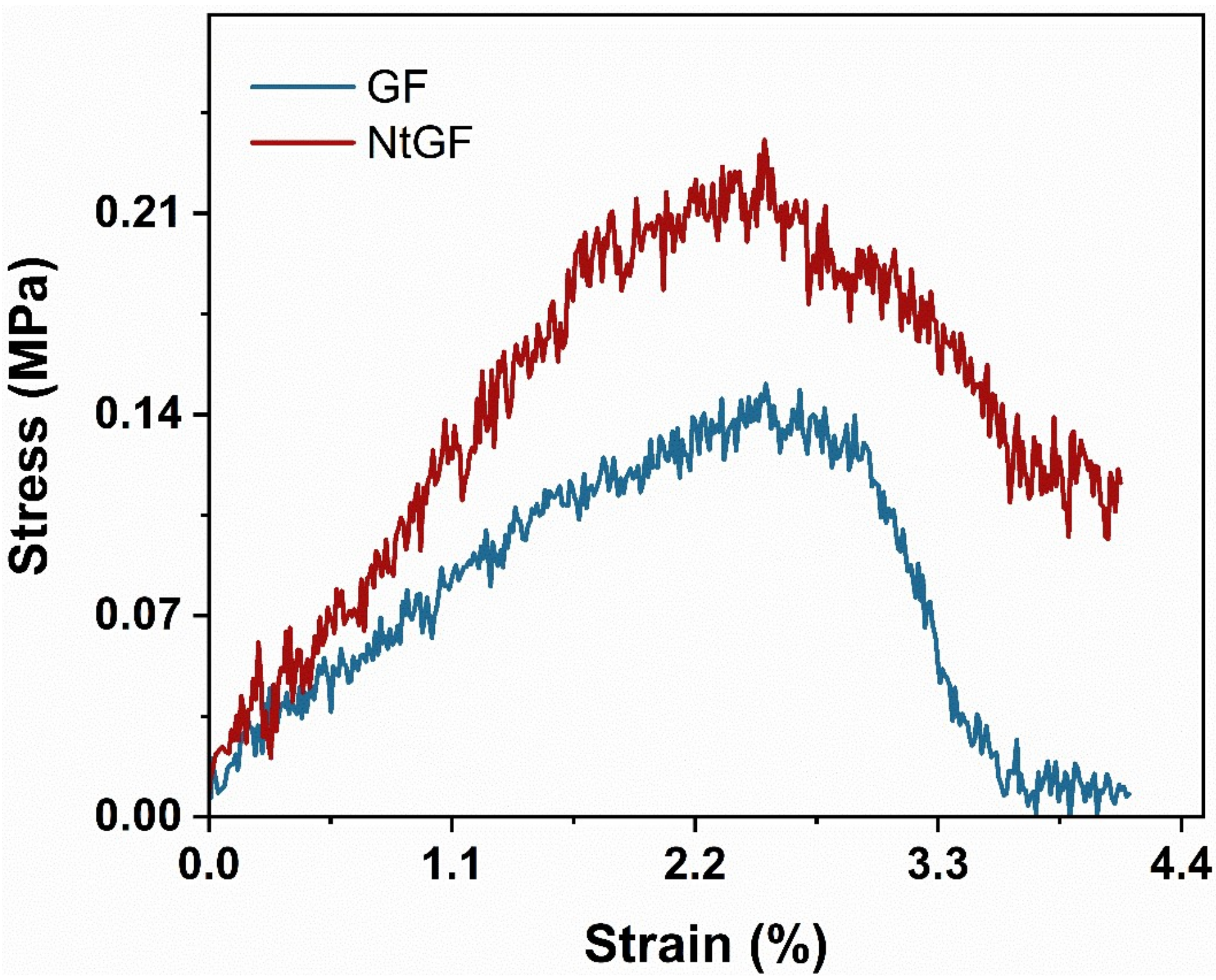


Figure S9. Tensile strength tests of the GF and NtGF separators.

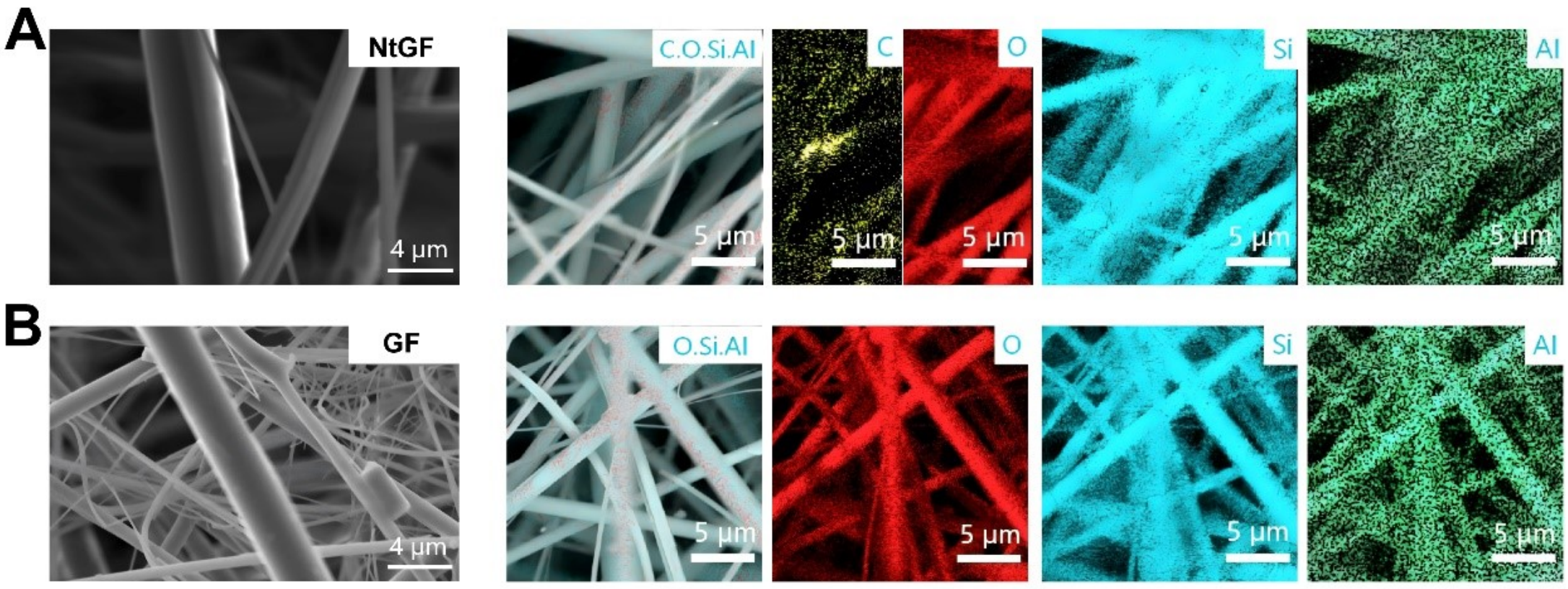


Figure S10. Surface SEM images and EDS mapping of (A) NtGF and (B) GF separators.

The uniform carbon signal (Figure S10A) indicates an even dispersion of Nt molecules on the GF microstructure. Comparing EDS mapping for pristine GF (Figure S10B), the NtGF mapping results reveals a consistent adherence of planar Nt units onto the surface of GF nanofibers. The multifunctional composite architecture is favorable for homogenizing $Zn^{2+}$ flux distribution during the Zn plating/stripping processes.

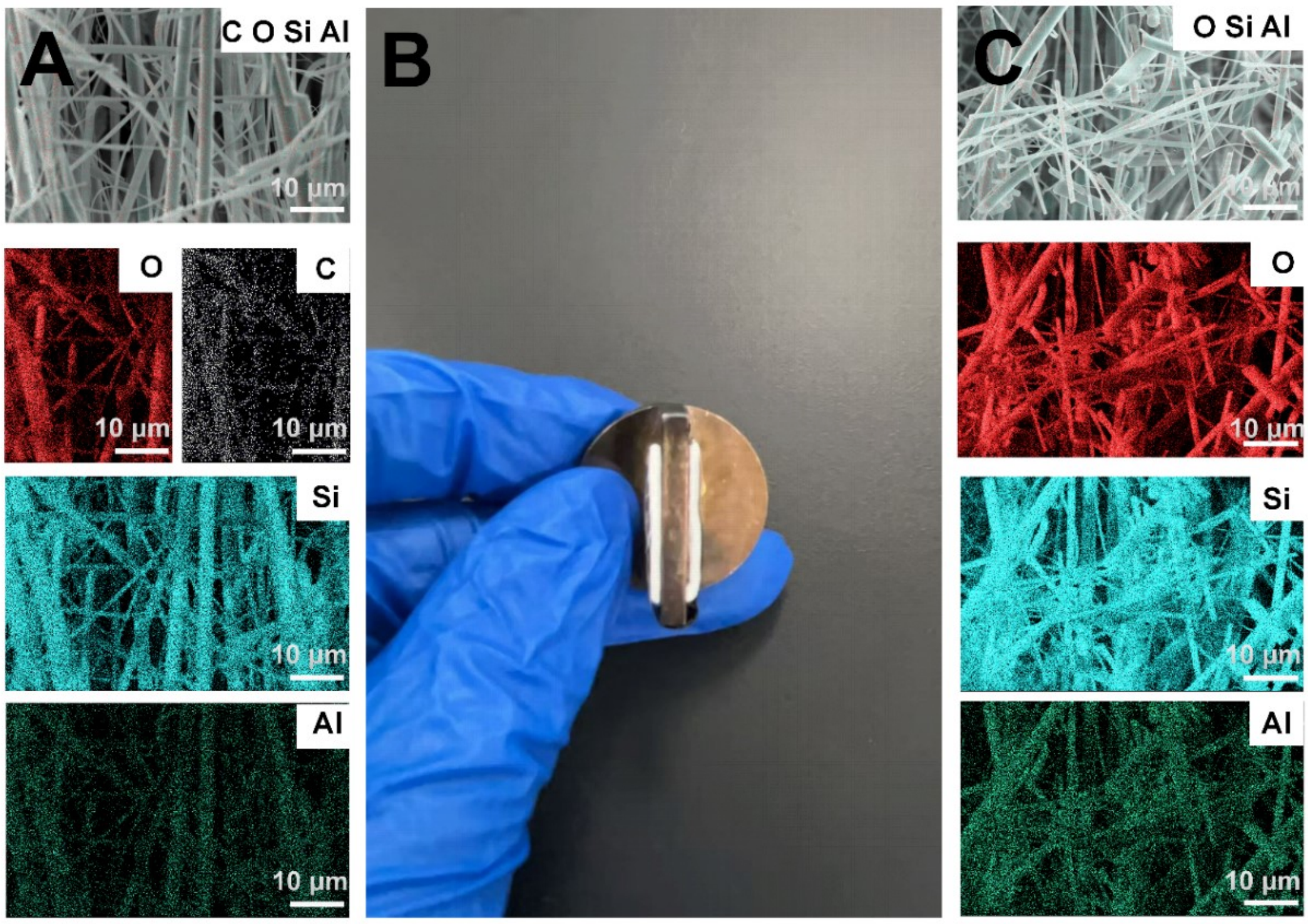


Figure S11. Cross-sectional SEM images of (A) NtGF and (C) GF separators. (B) The optical images of cross-sectional NtGF and GF separators (The separators were cut with scissors in air).

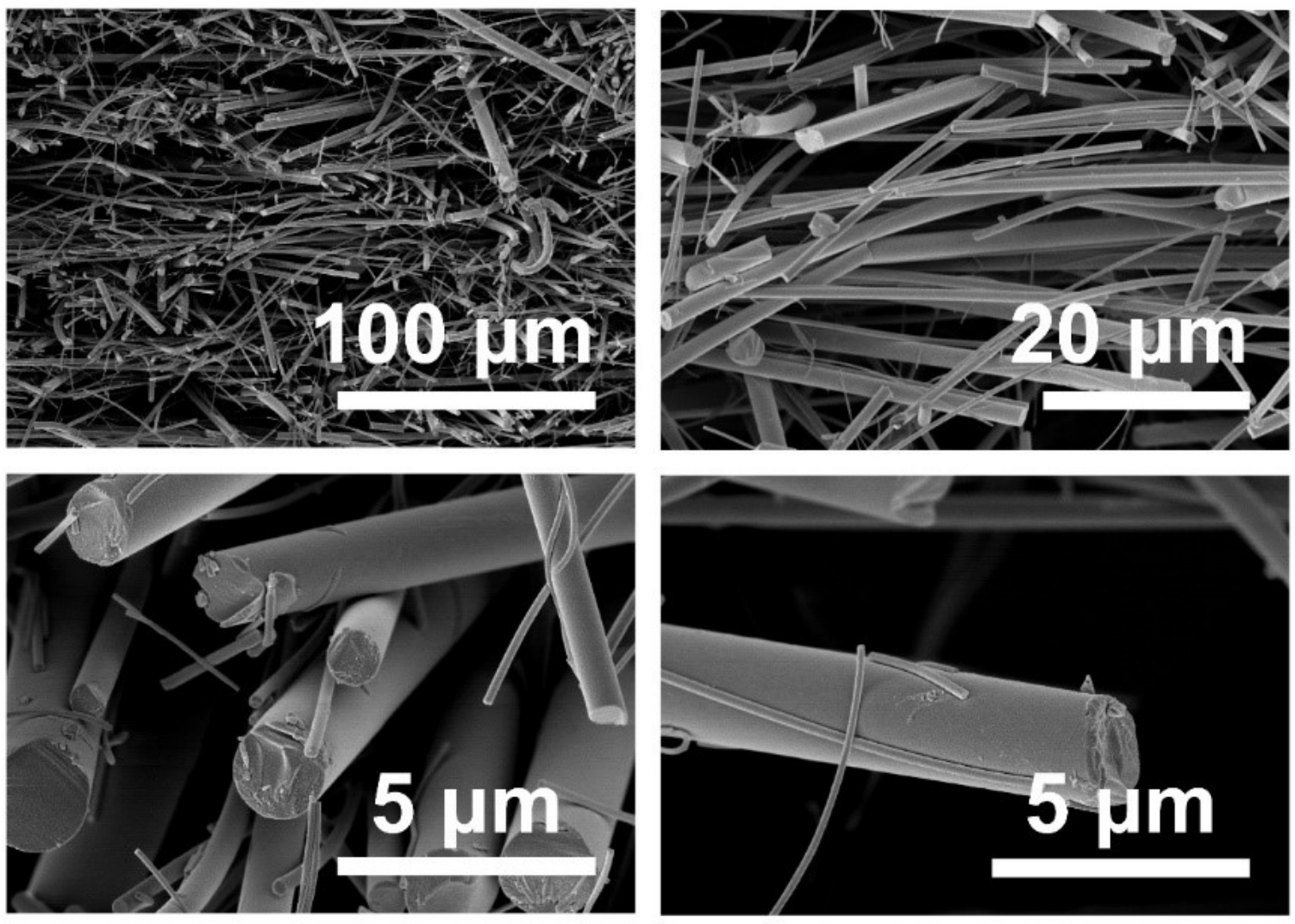


Figure S12. Cross-sectional SEM images of NtGF separator. (The separator was fractured in liquid nitrogen).

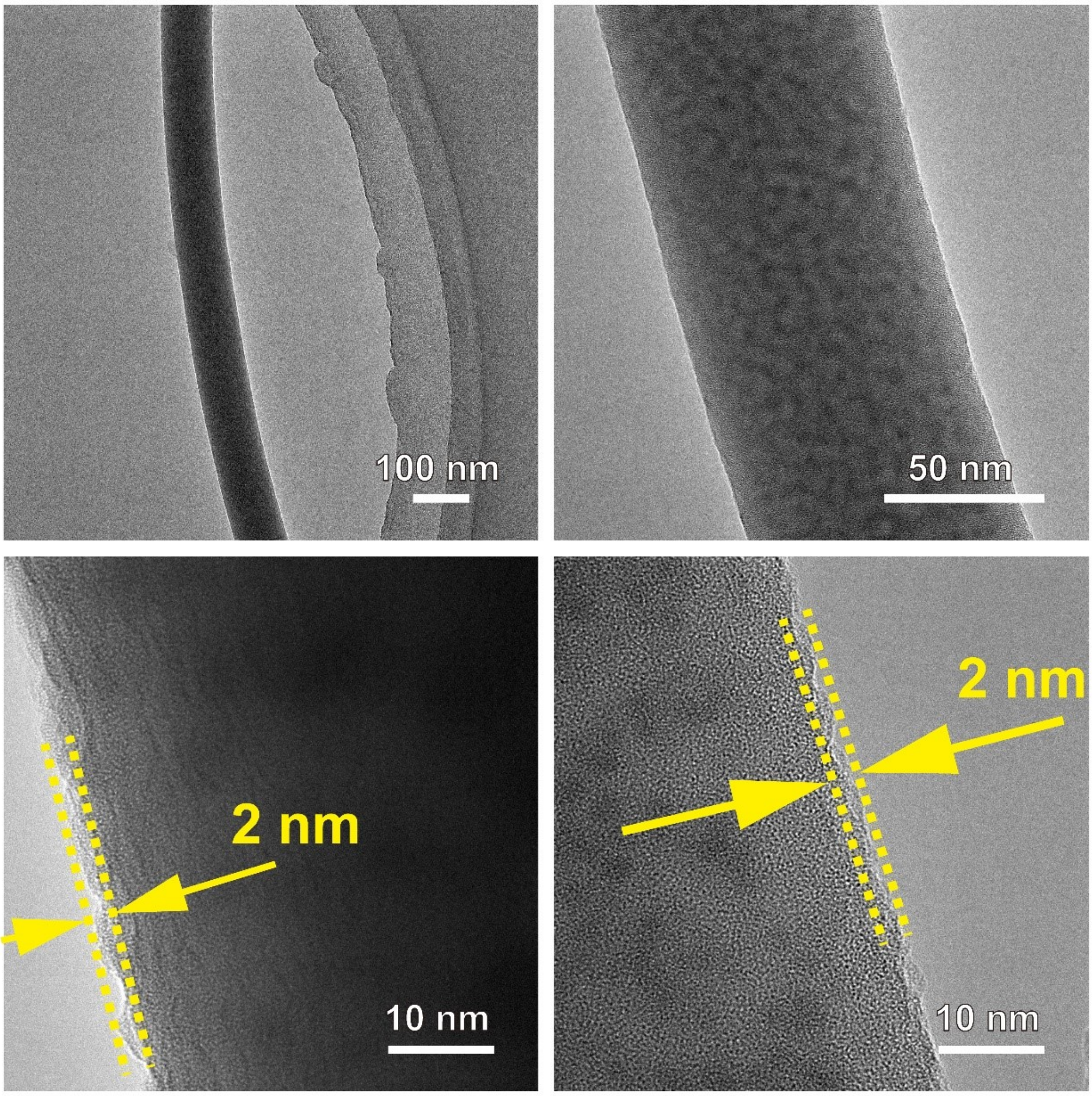


Figure S13. TEM images of NtGF separators.

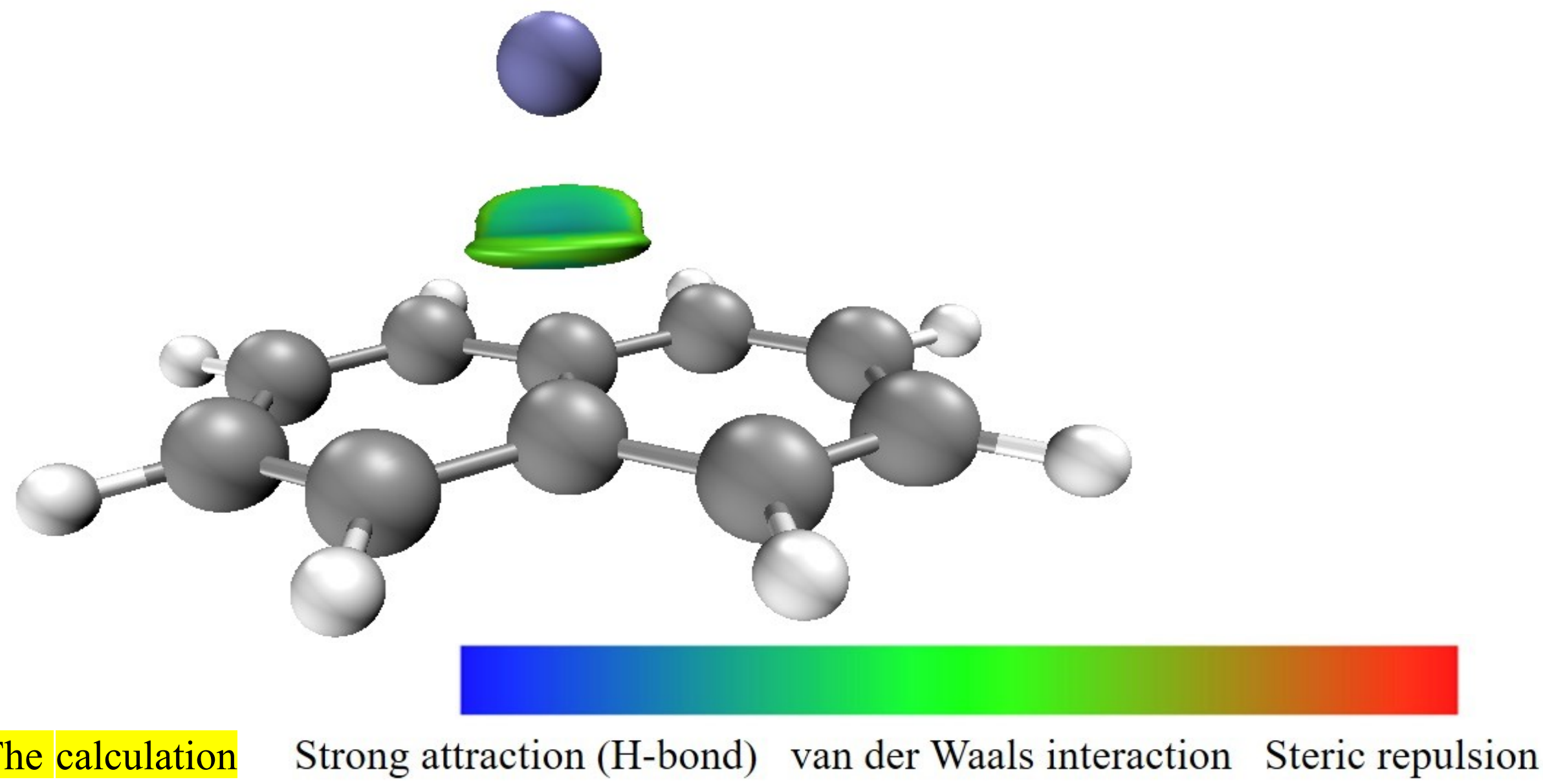


Figure S14. The calculation isosurface model for the interaction between $Zn^{2+}$ and Nt.

As shown in the calculated charge density difference (CDD) isosurface, the $Zn^{2+}$ resides directly above the centroid of the fused bicyclic ring system of naphthalene (Nt). A pancake-shaped isosurface extends across the entire π-plane, indicating that the cation ($Zn^{2+}$)-Nt interaction is not a localized point-to-point contact but rather a delocalized face-to-face interaction with the aromatic π-system, which is characteristic of $Zn^{2+}$-π interactions. Notably, the isosurface is predominantly green, with a teal/blue-green patch concentrated near the ring center. The prevailing green color suggests that the overall interaction is governed by van der Waals/dispersion forces, while the central teal/blue-green region implies a moderately enhanced attractive interaction directly above the ring centroid, indicative of a partial cation-π character. Collectively, the $Zn^{2+}$-Nt interaction can be described as a dispersion-dominated, face-to-face interaction with a non-negligible $Zn^{2+}$-π contribution localized at the ring center.

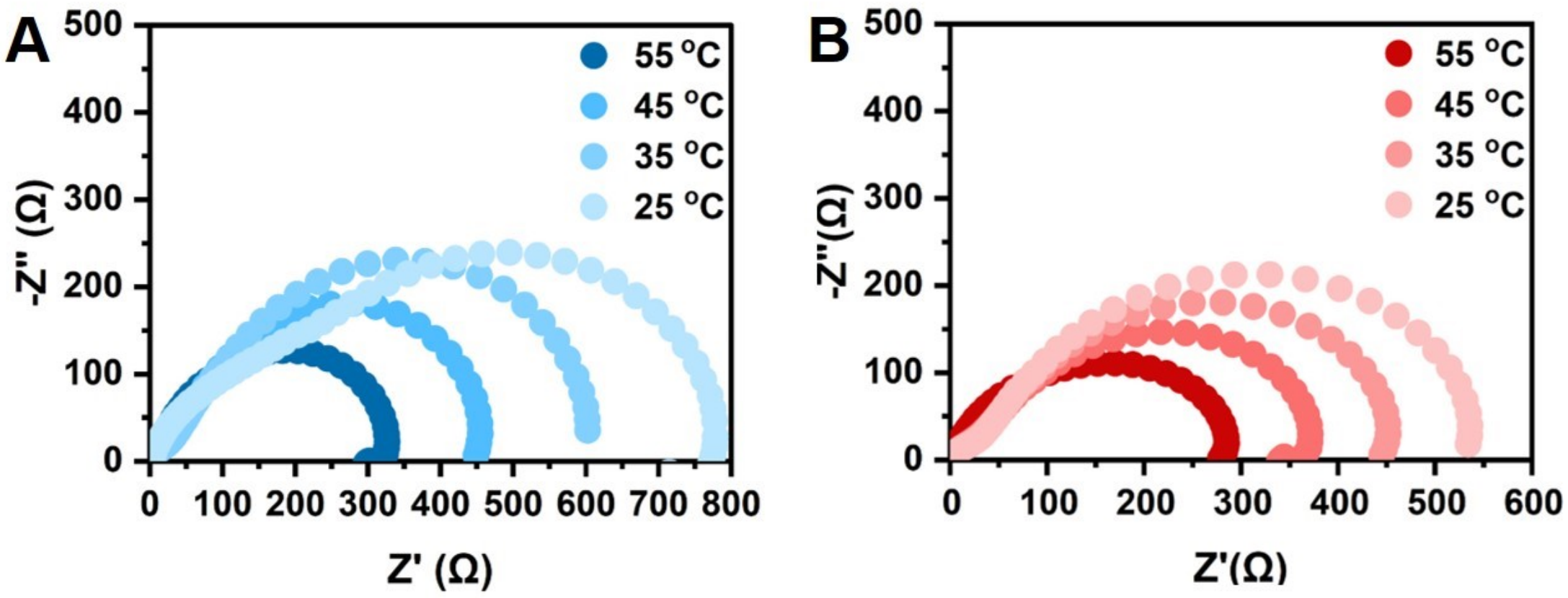


Figure S15. EIS spectra under different temperatures for Zn//Zn symmetrical cells using (A) GF separator and (B) NtGF separator, respectively.

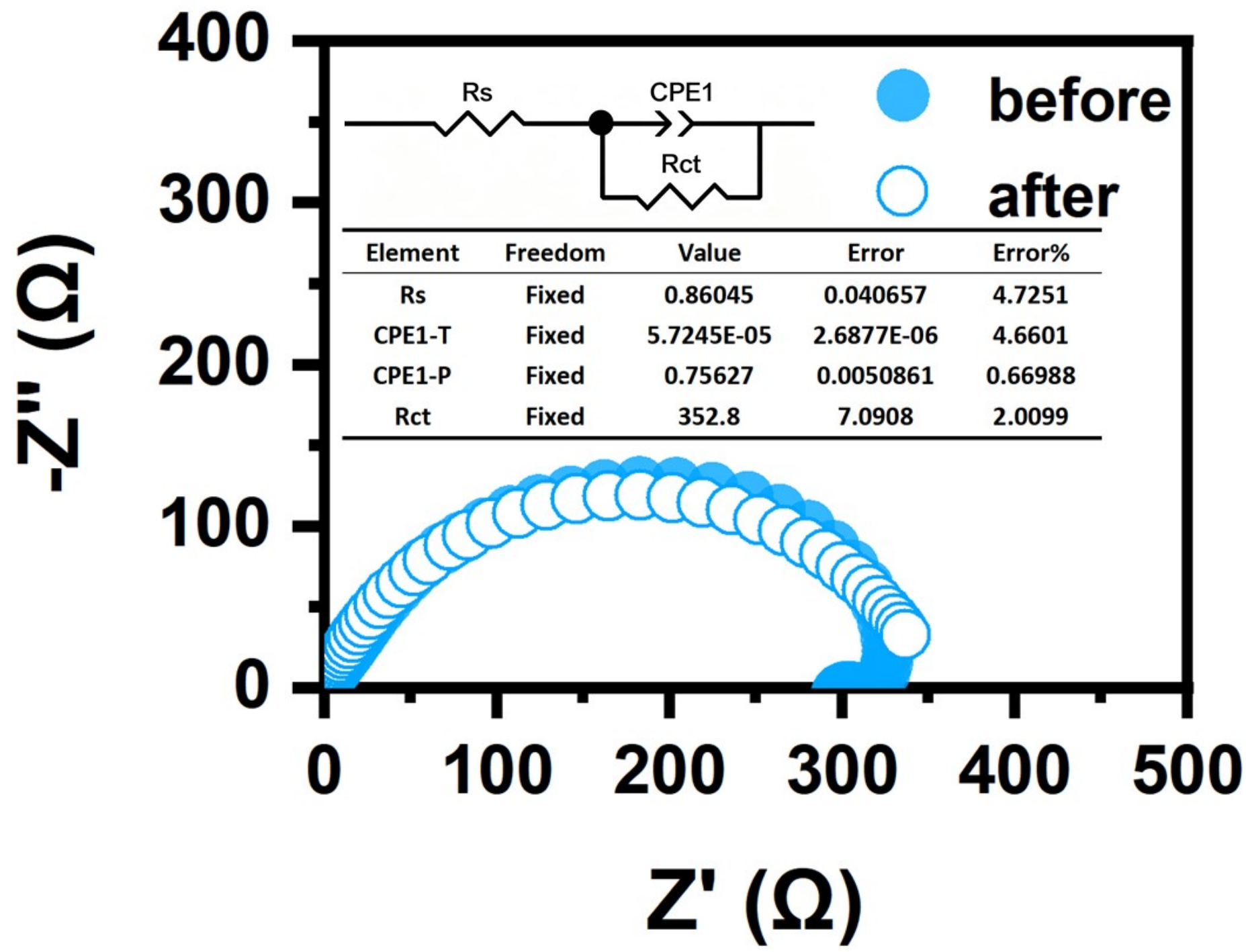


| Element | Freedom | Value | Error | Error% |
|---|---|---|---|---|
| Rs | Fixed | 0.86045 | 0.040657 | 4.7251 |
| CPE1-T | Fixed | 5.7245E-05 | 2.6877E-06 | 4.6601 |
| CPE1-P | Fixed | 0.75627 | 0.0050861 | 0.66988 |
| Rct | Fixed | 352.8 | 7.0908 | 2.0099 |

Figure S16. The EIS fitting results of the GF separator at 55 $^{o}$C.

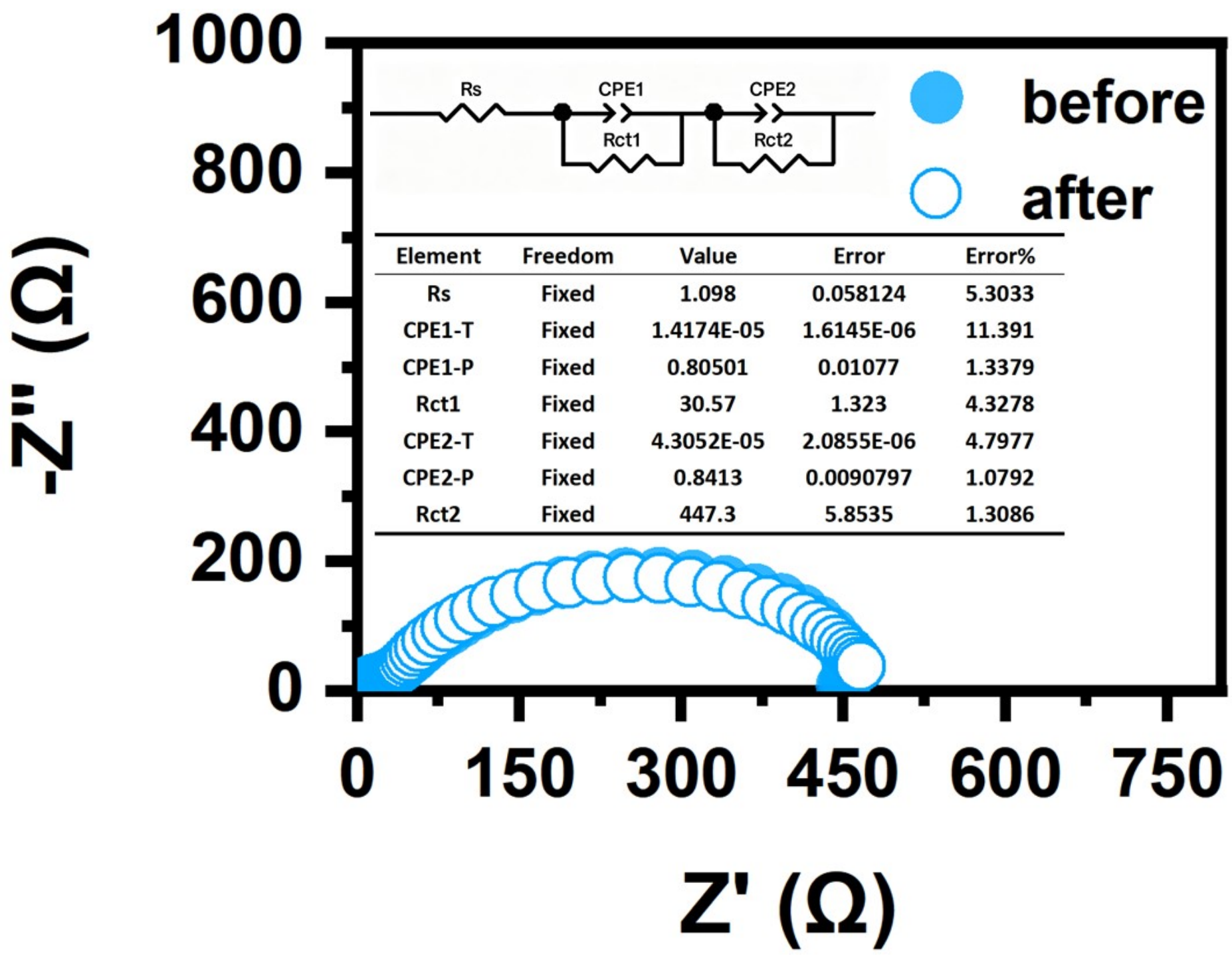


Figure S17. The EIS fitting results of the GF separator at 45 $^{o}$C.

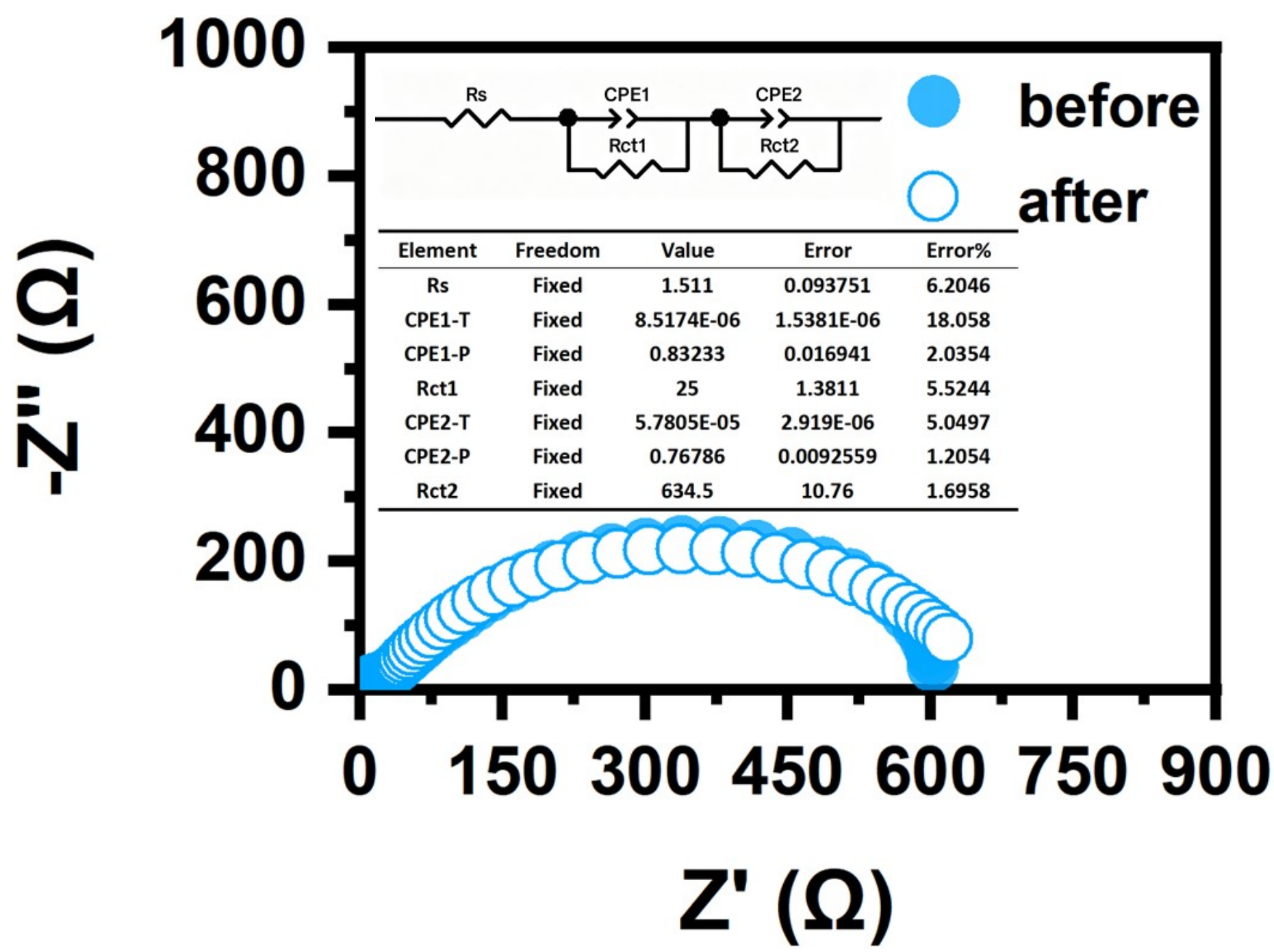


Figure S18. The EIS fitting results of the GF separator at 35 $^{o}$C.

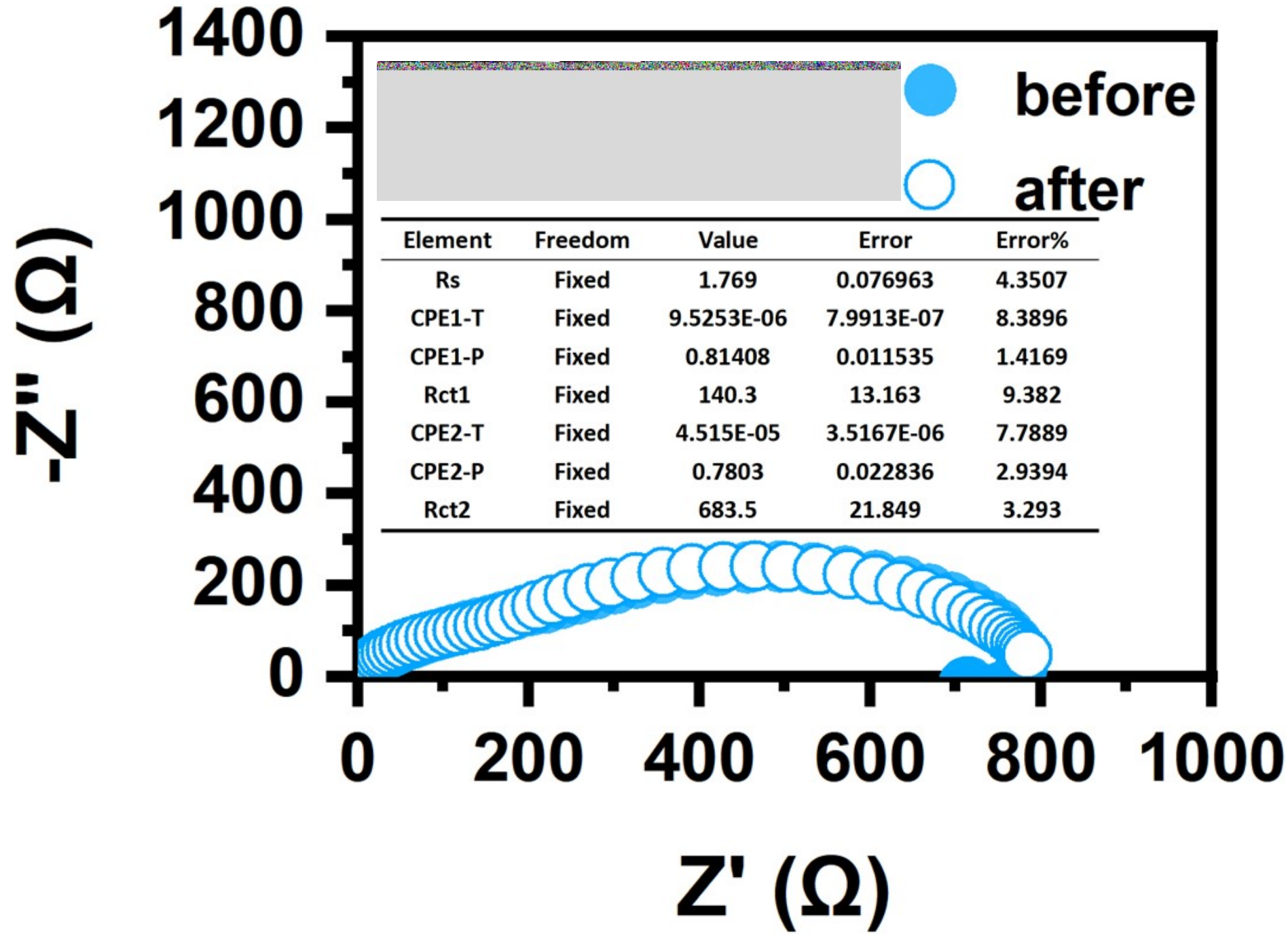


Figure S19. The EIS fitting results of the GF separator at 25 $^{o}$C.

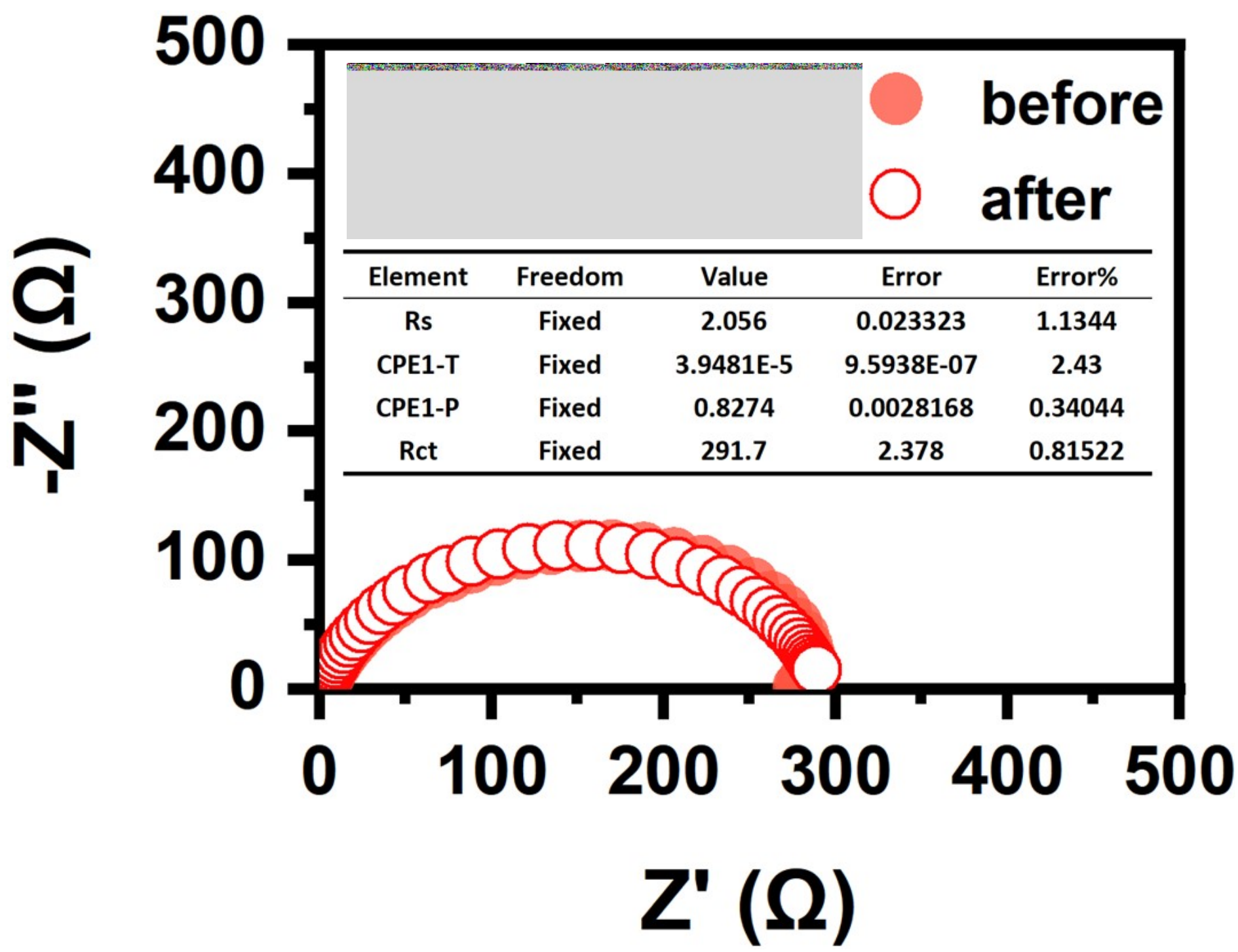


Figure S20. The EIS fitting results of the NtGF separator at 55 $^{o}$C.

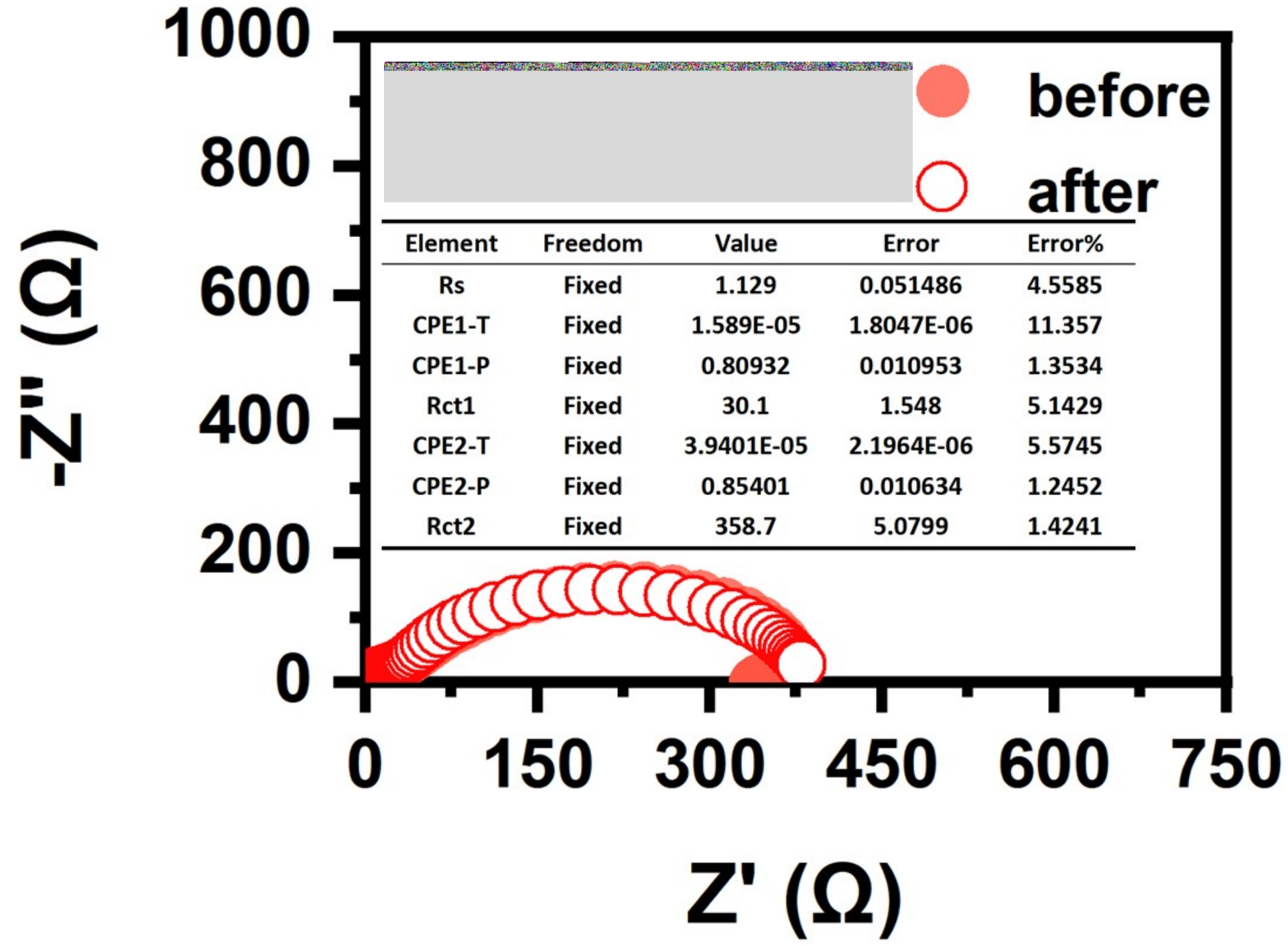


Figure S21. The EIS fitting results of the NtGF separator at 45 ºC.

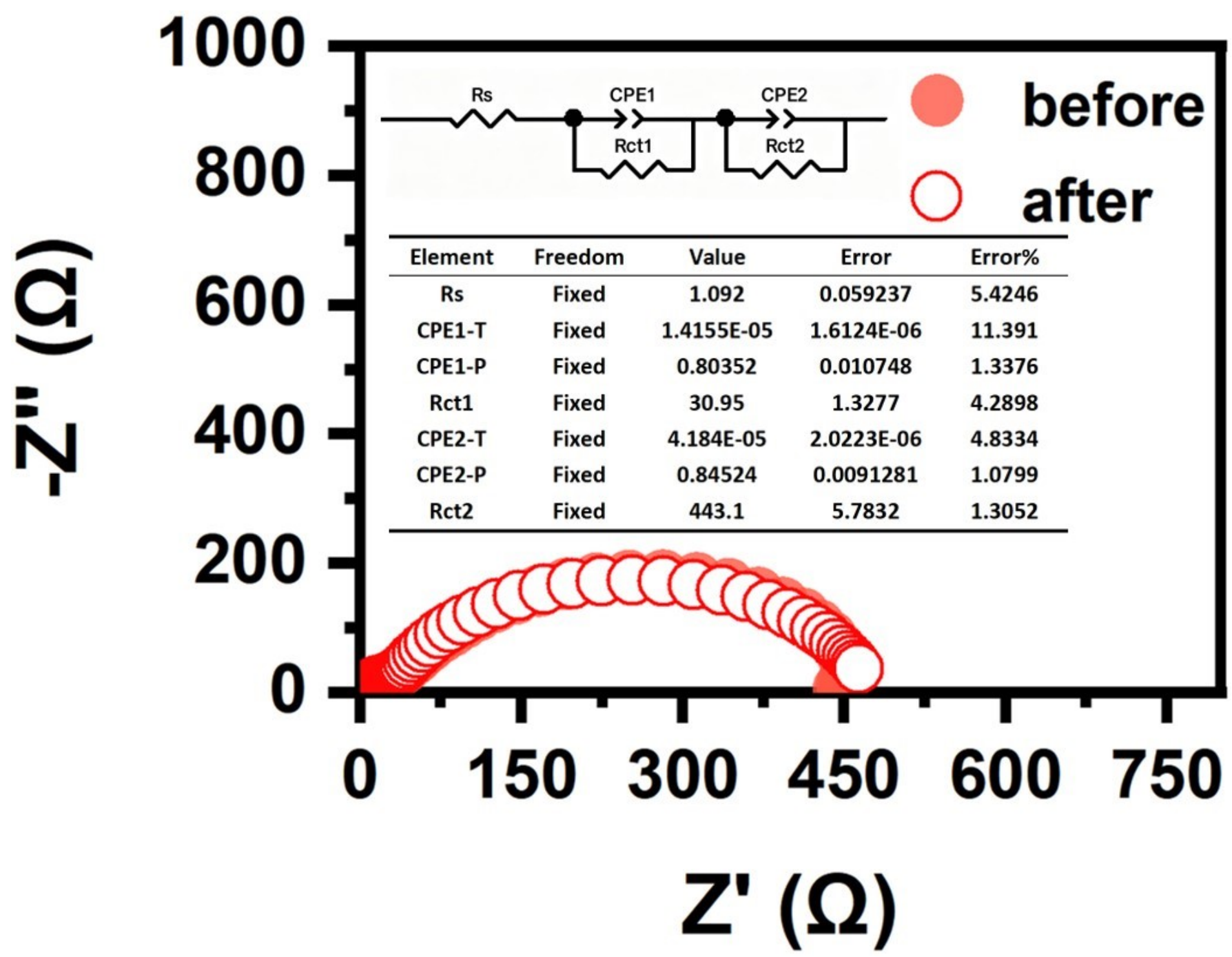


Figure S22. The EIS fitting results of the NtGF separator at 35 ºC.

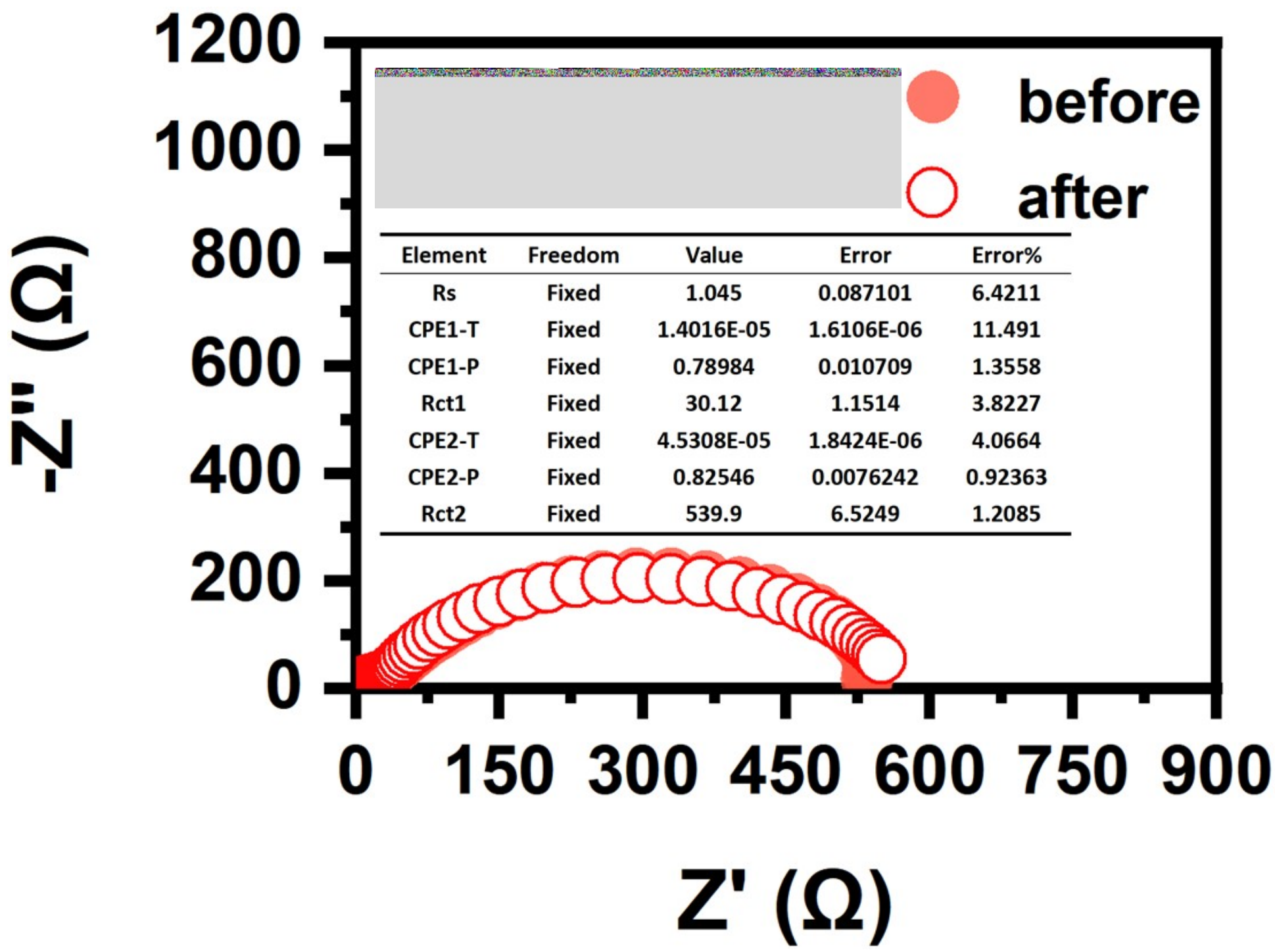


Figure S23. The EIS fitting results of the NtGF separator at 25 $^{o}$C.

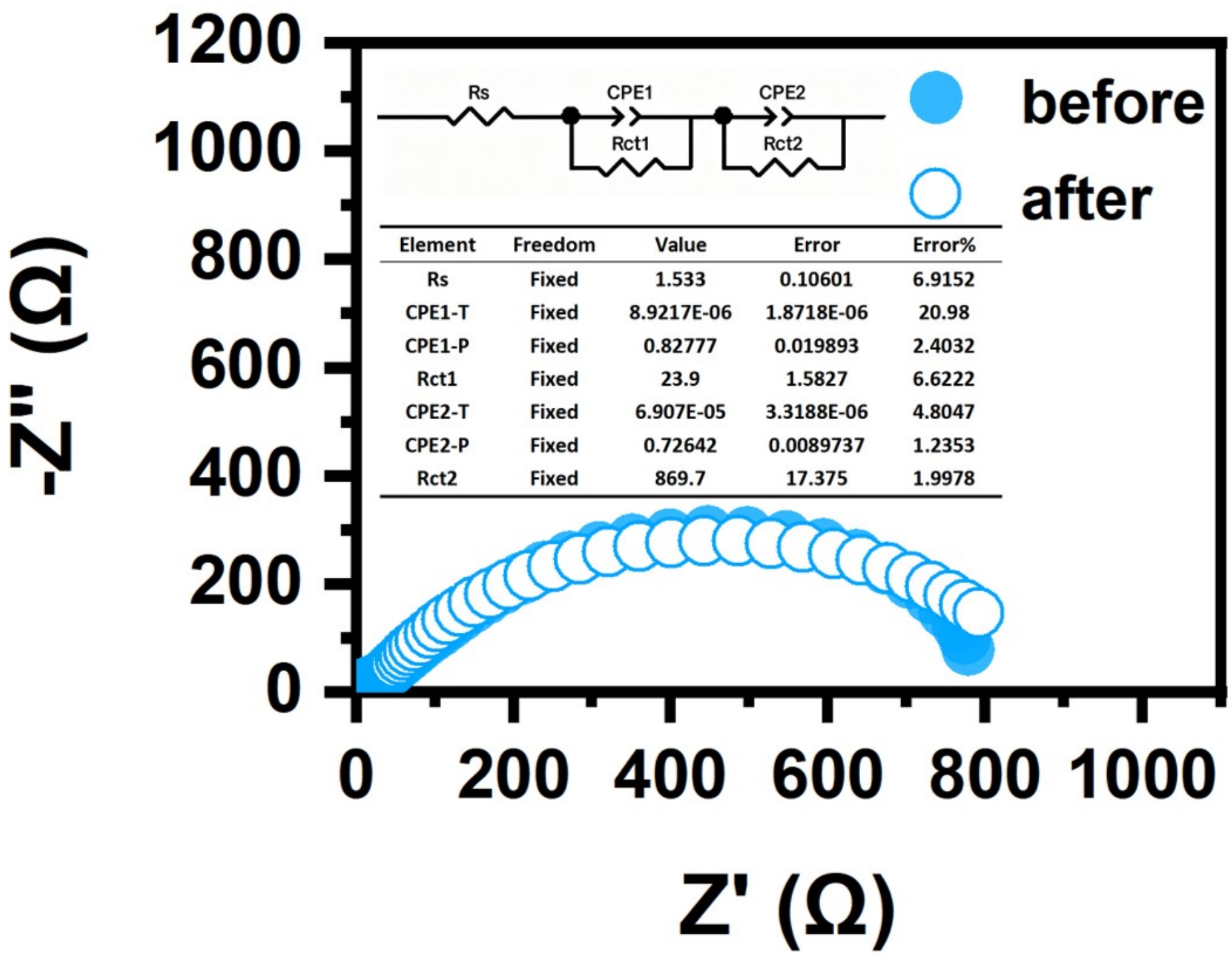


Figure S24. The EIS fitting results of the GF separator before polarization.

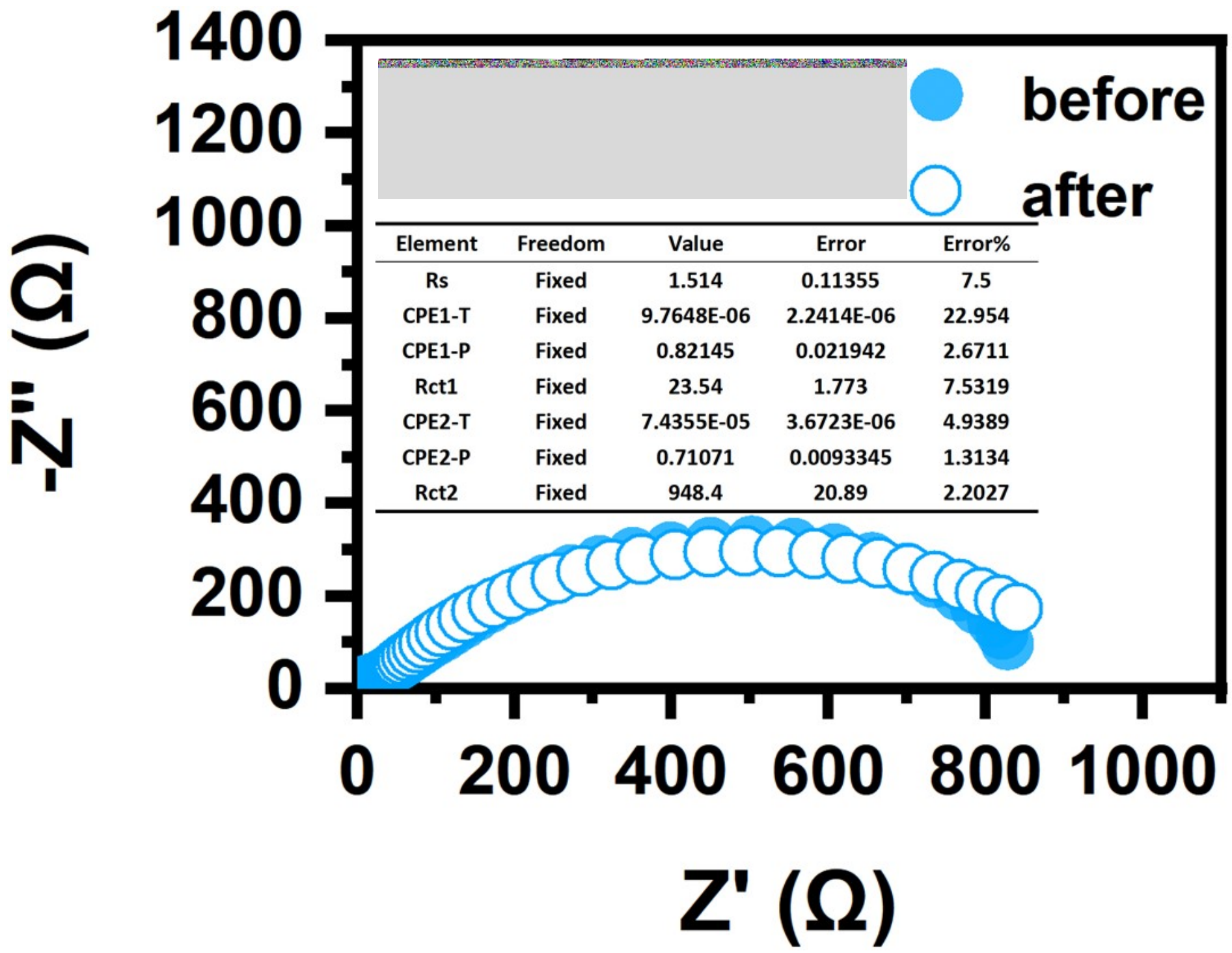


Figure S25. The EIS fitting results of the GF separator after polarization.

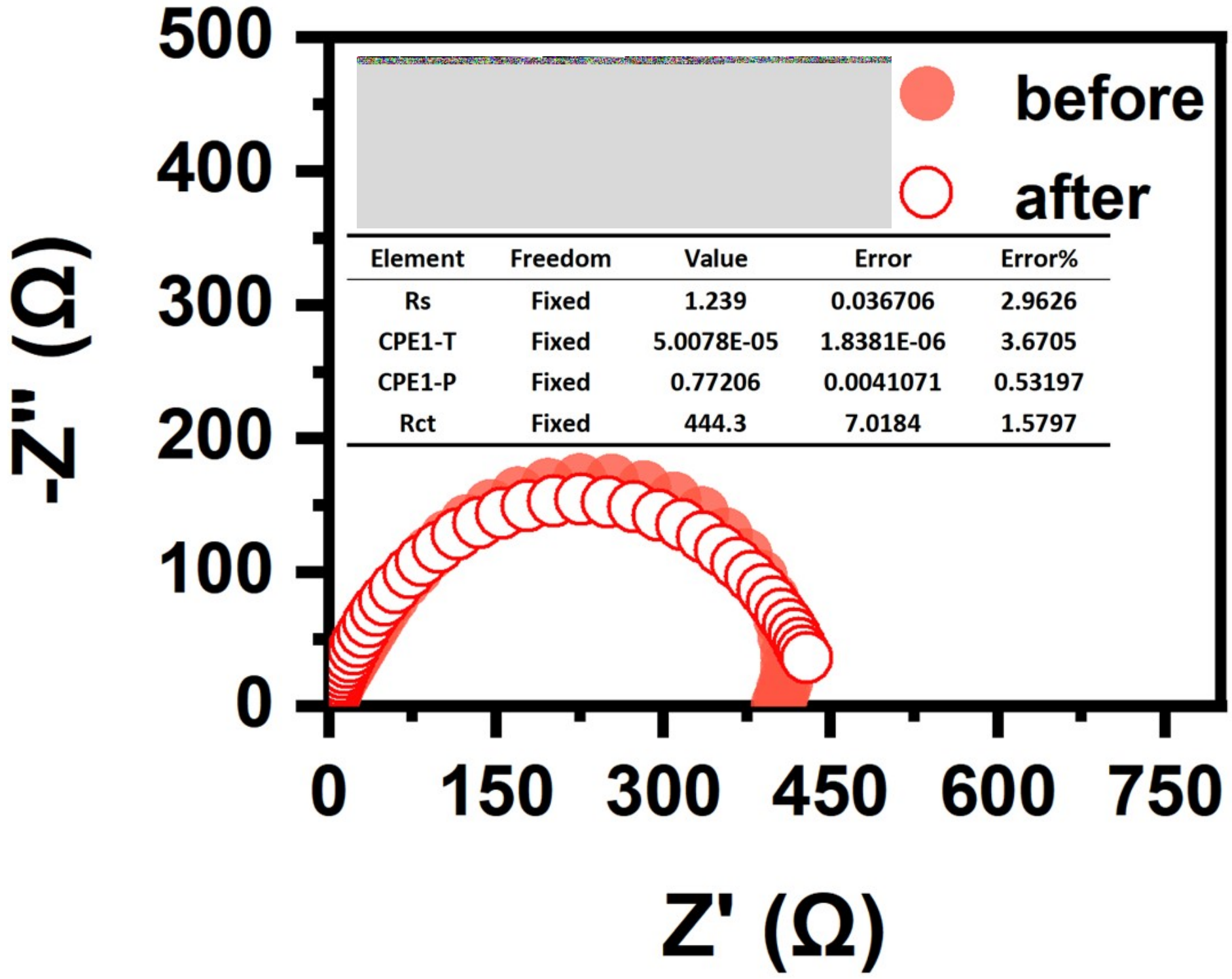


Figure S26. The EIS fitting results of the NtGF separator before polarization.

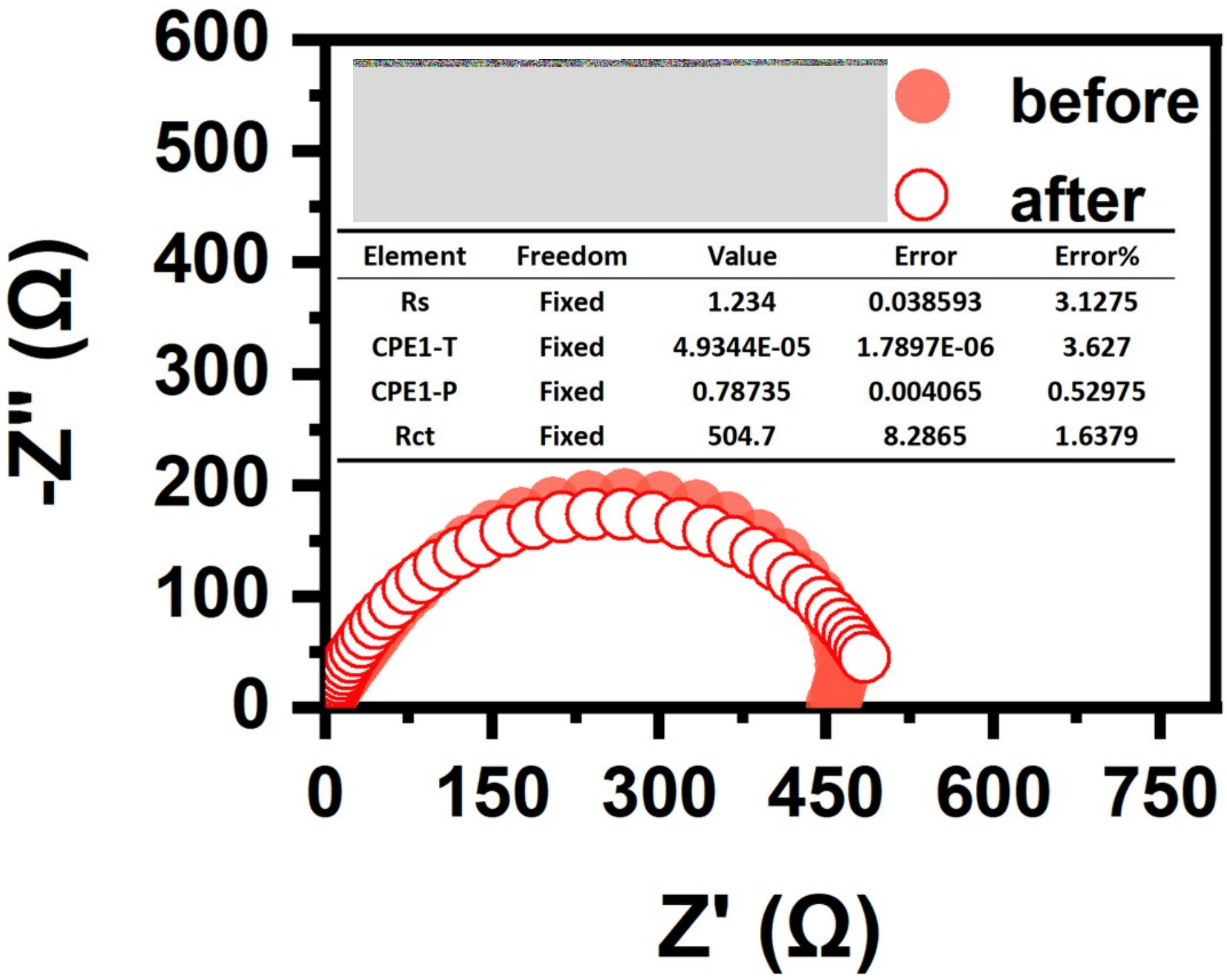


Figure S27. The EIS fitting results of the NtGF separator after polarization.

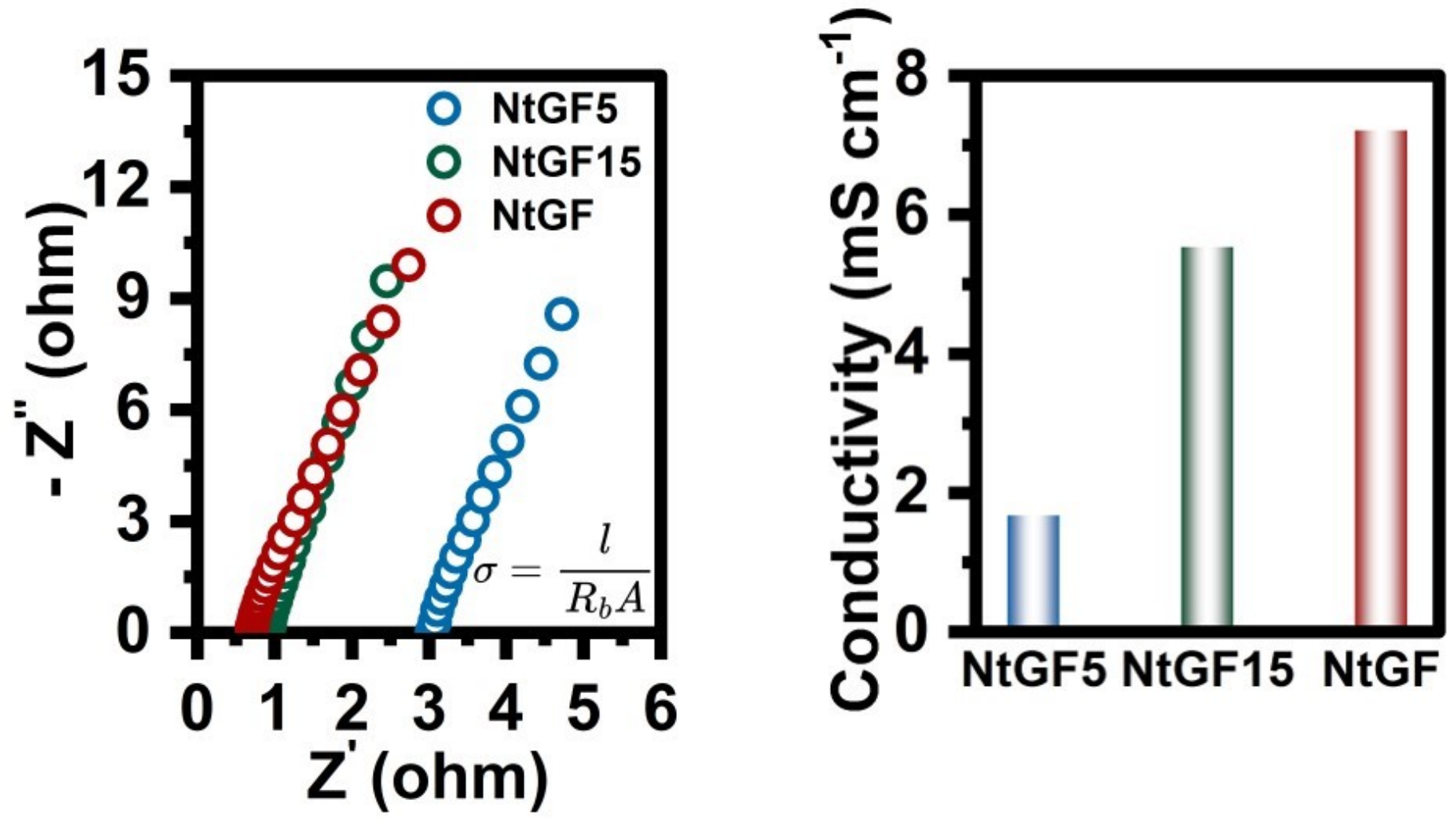


Figure S28. Ionic conductivities from different Nt/DME solutions at various Nt concentrations. The NtGF separators fabricated from Nt/DME solutions at concentrations of 0.005, 0.01, and 0.015 mol/L are designated as NtGF5, NtGF, and NtGF15, respectively.

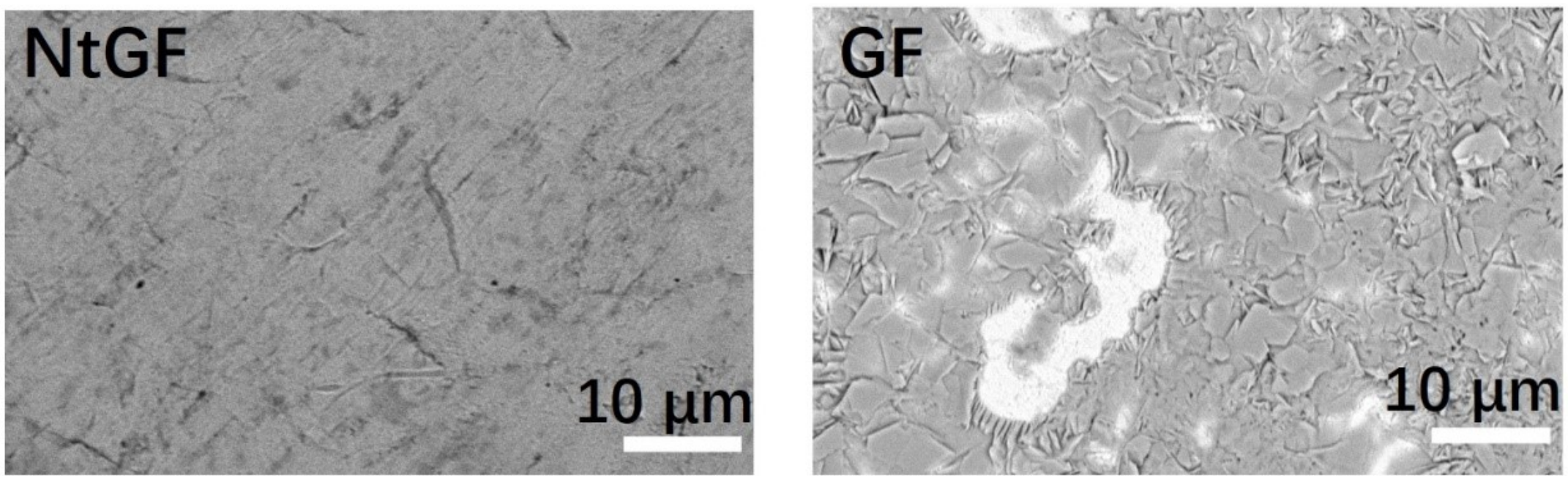


Figure S29. The SEM images of the Zn electrodes from Zn/Zn symmetrical cells using a current density of 2 mA $cm^{-2}$ and areal capacity of 2 mAh $cm^{-2}$.

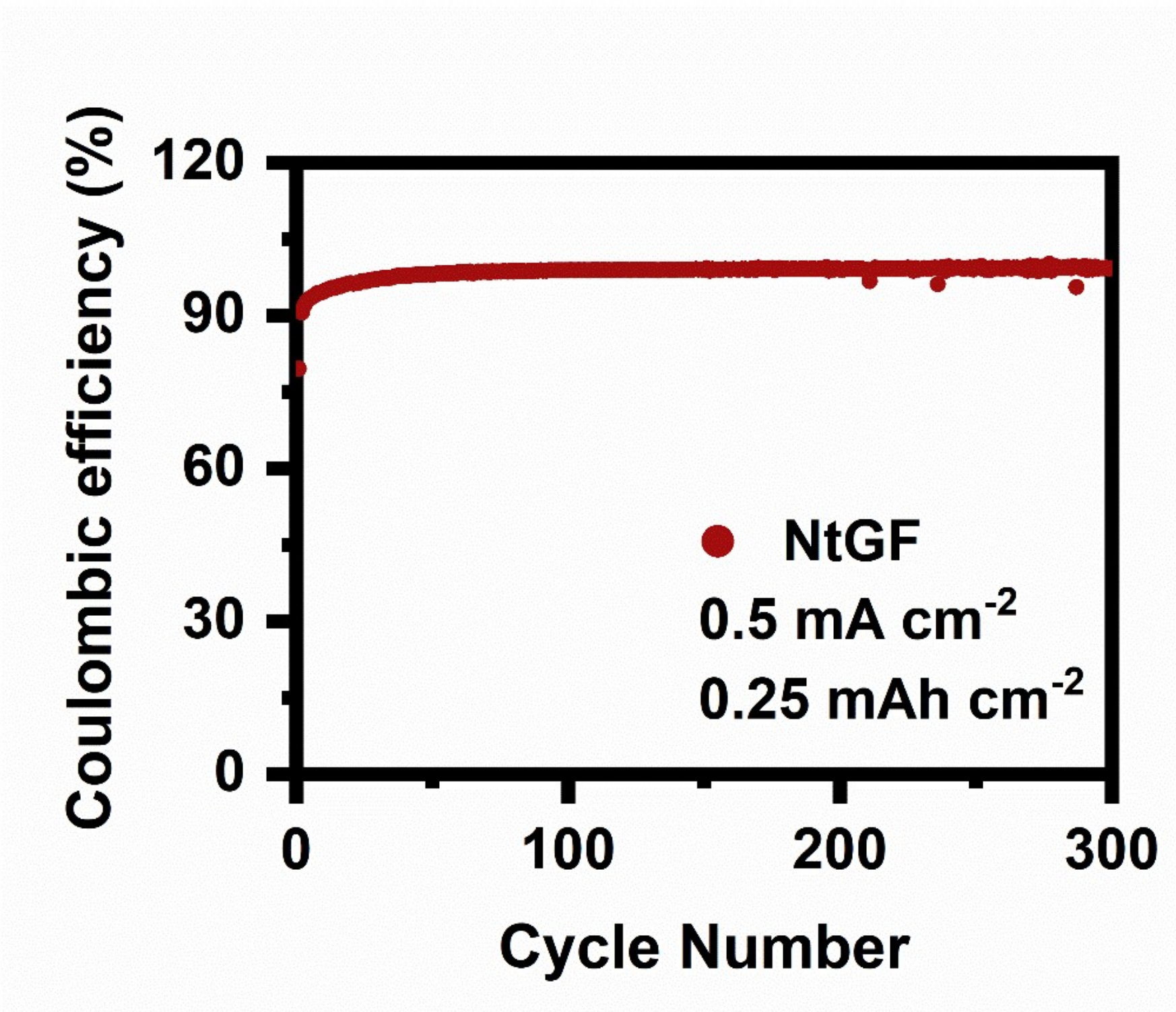


Figure S30. The coulombic efficiency (CE) of the Zn//Cu cells with a current density of 0.5 mA $cm^{-2}$ and areal capacity of 0.25 mAh $cm^{-2}$.

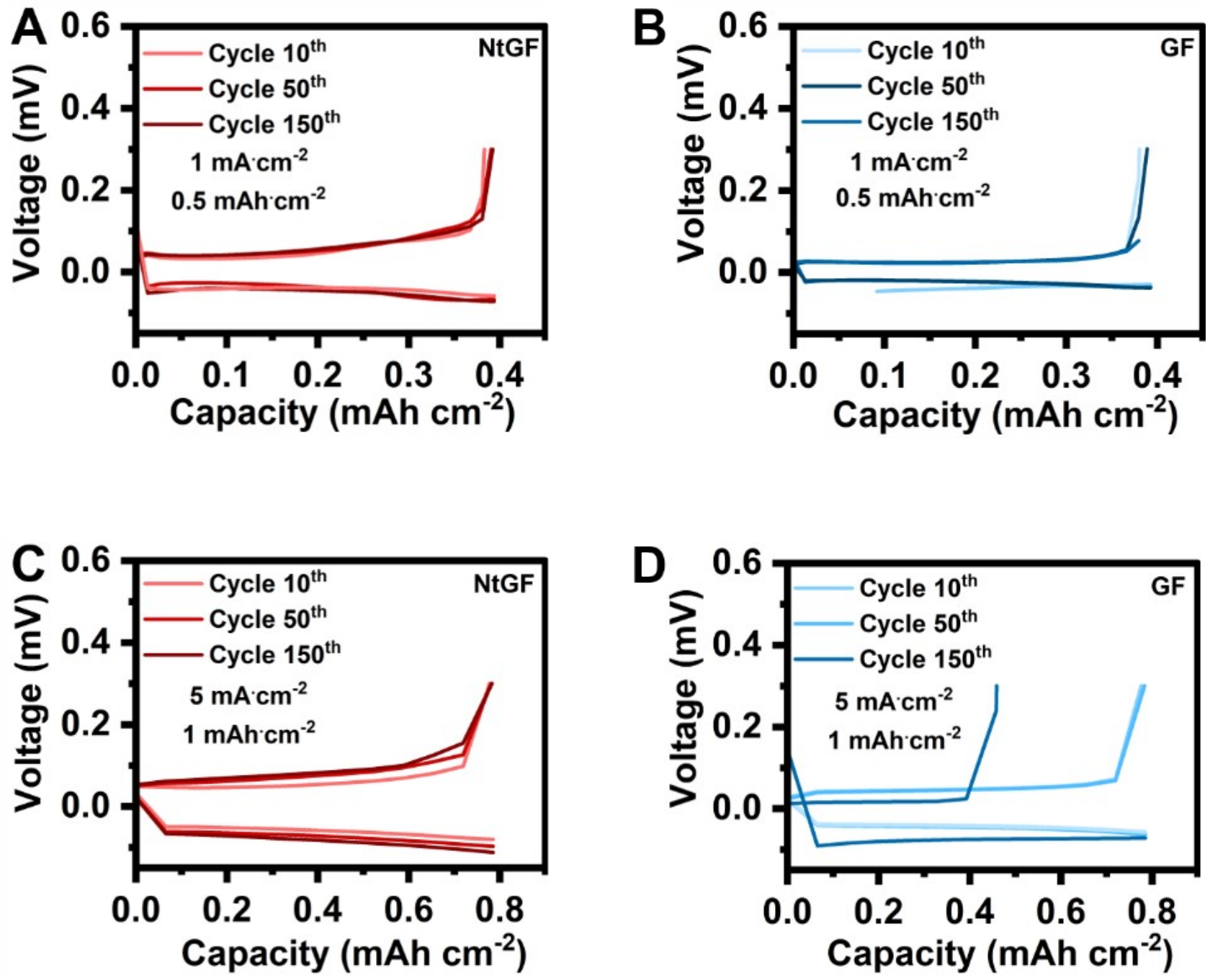


Figure S31. The corresponding voltage-capacity profiles of Zn//Cu cells at various cycling numbers derived from Figure 2E and F. (A and B) The voltage-capacity profiles at 1 mA $cm^{-2}$ and 0.5 mAh $cm^{-2}$. (C and D) The voltage-capacity profiles at 5 mA $cm^{-2}$ and 1 mAh $cm^{-2}$.

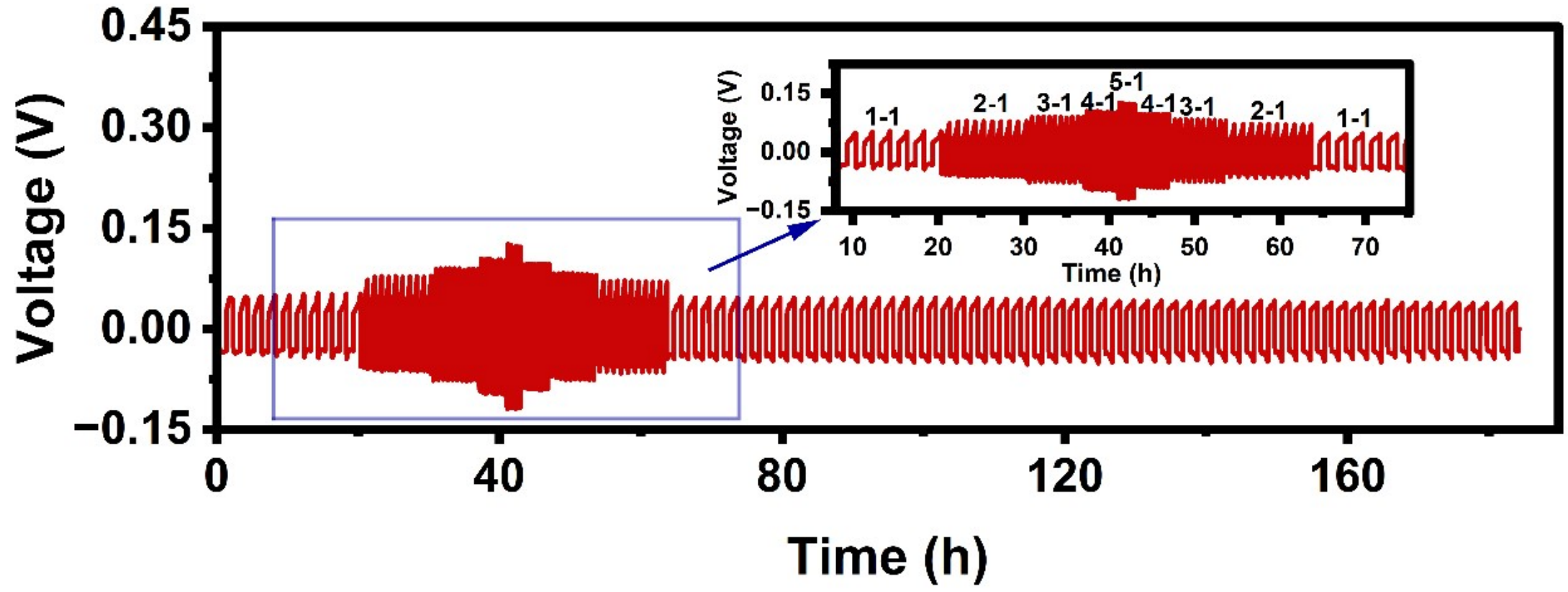


Figure S32. Rate performance of Zn//Zn symmetrical cells using NtGF separator.

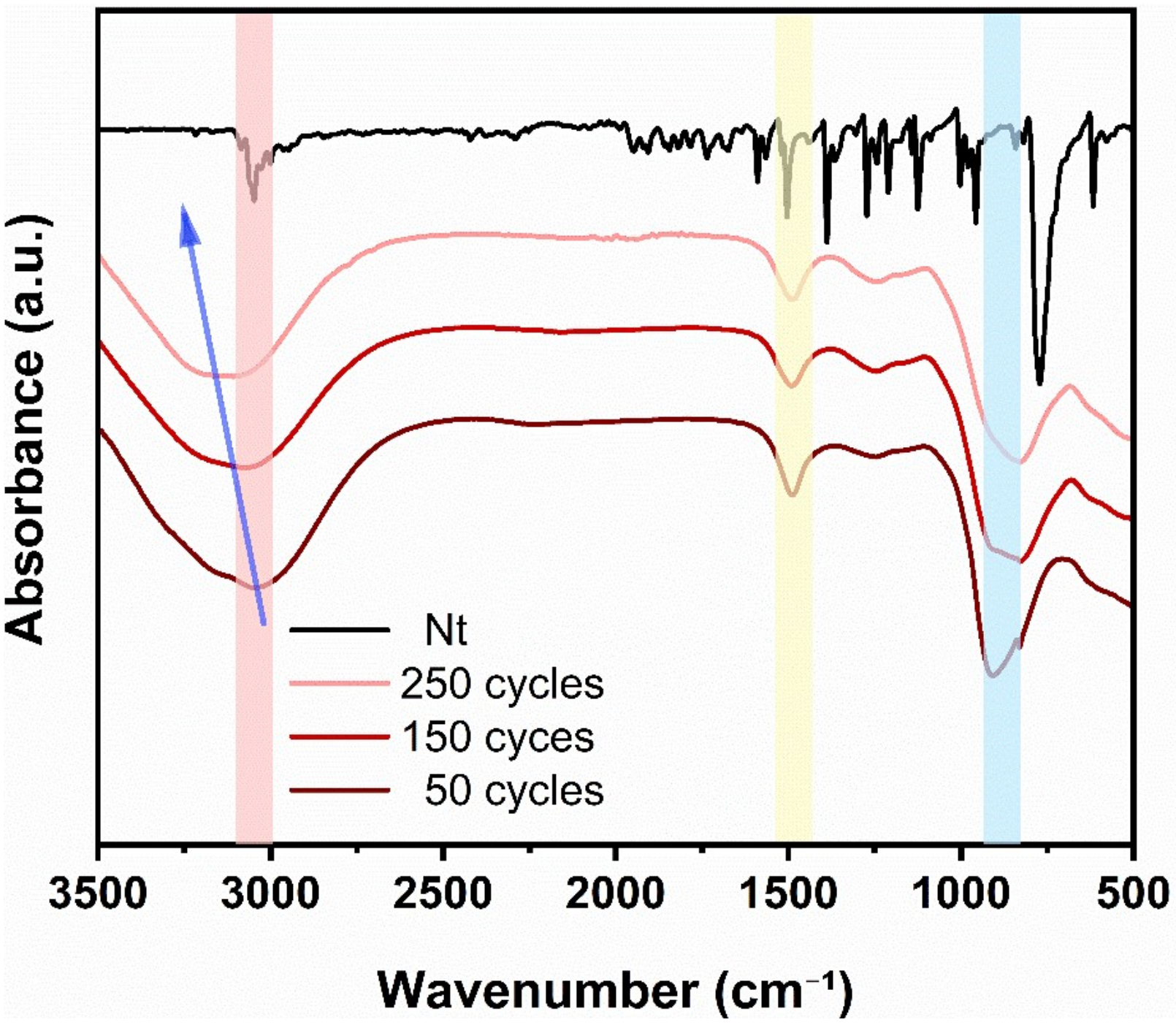


Figure S33. The FTIR spectra of the Nt and NtGF separators under different plating/stripping cycles.

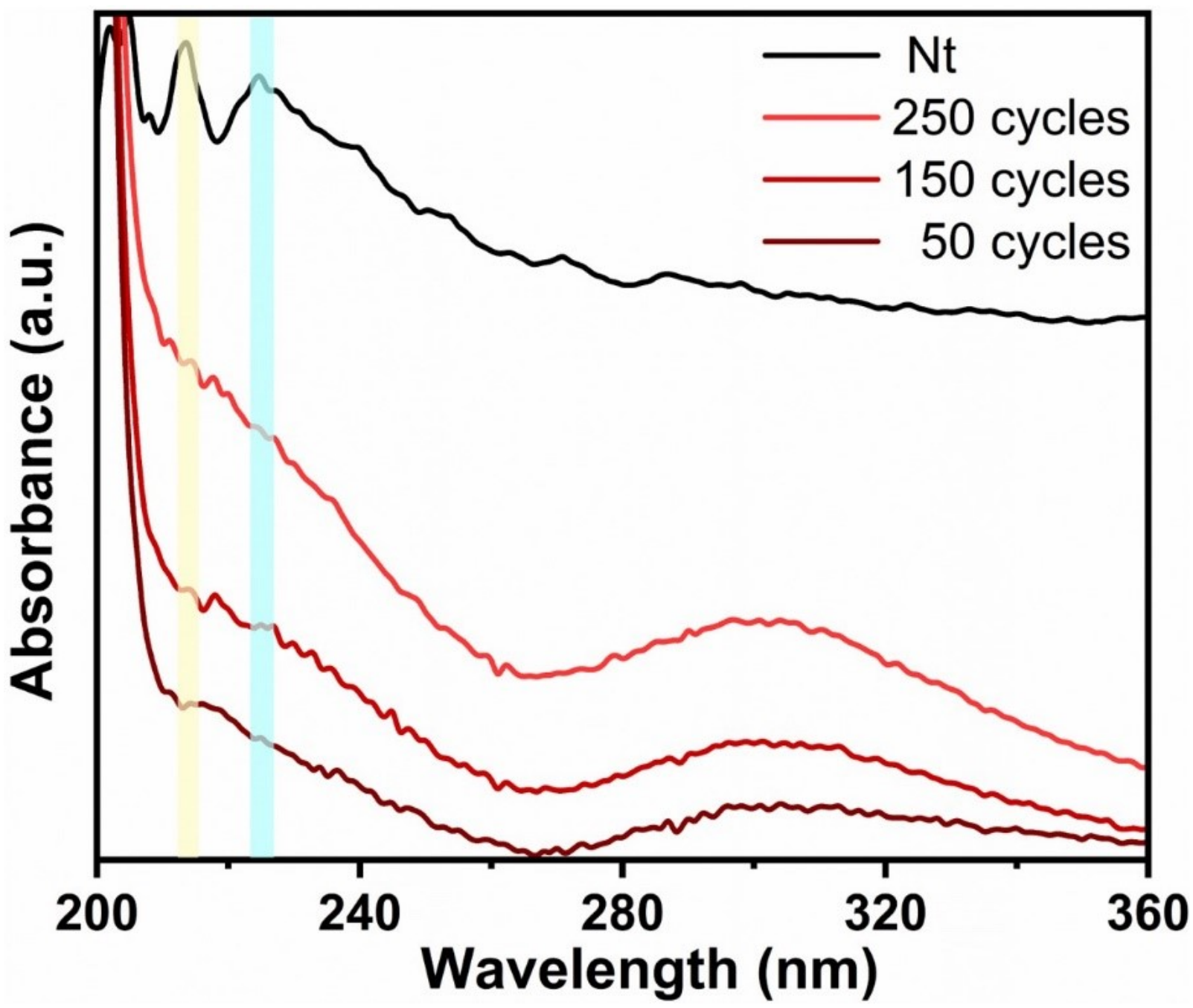


Figure S34. UV-vis absorption spectra of the Nt and NtGF separators-soaked $ZnSO_4$ electrolyte under different Zn deposition cycles.

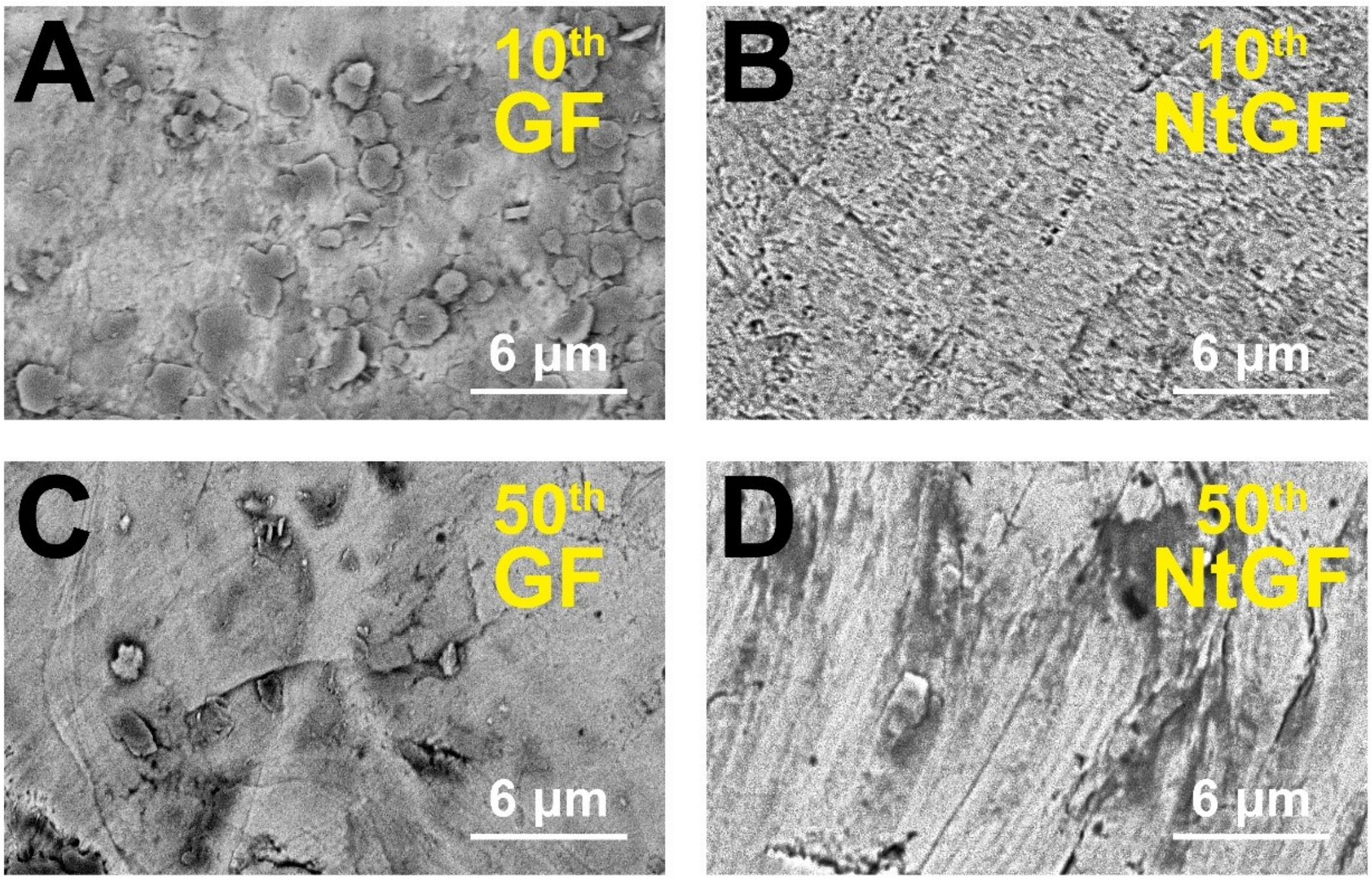


Figure S35. The Zn deposition details under enlarged magnifications for Zn plating when used GF (A) and NtGF (B) as separators.

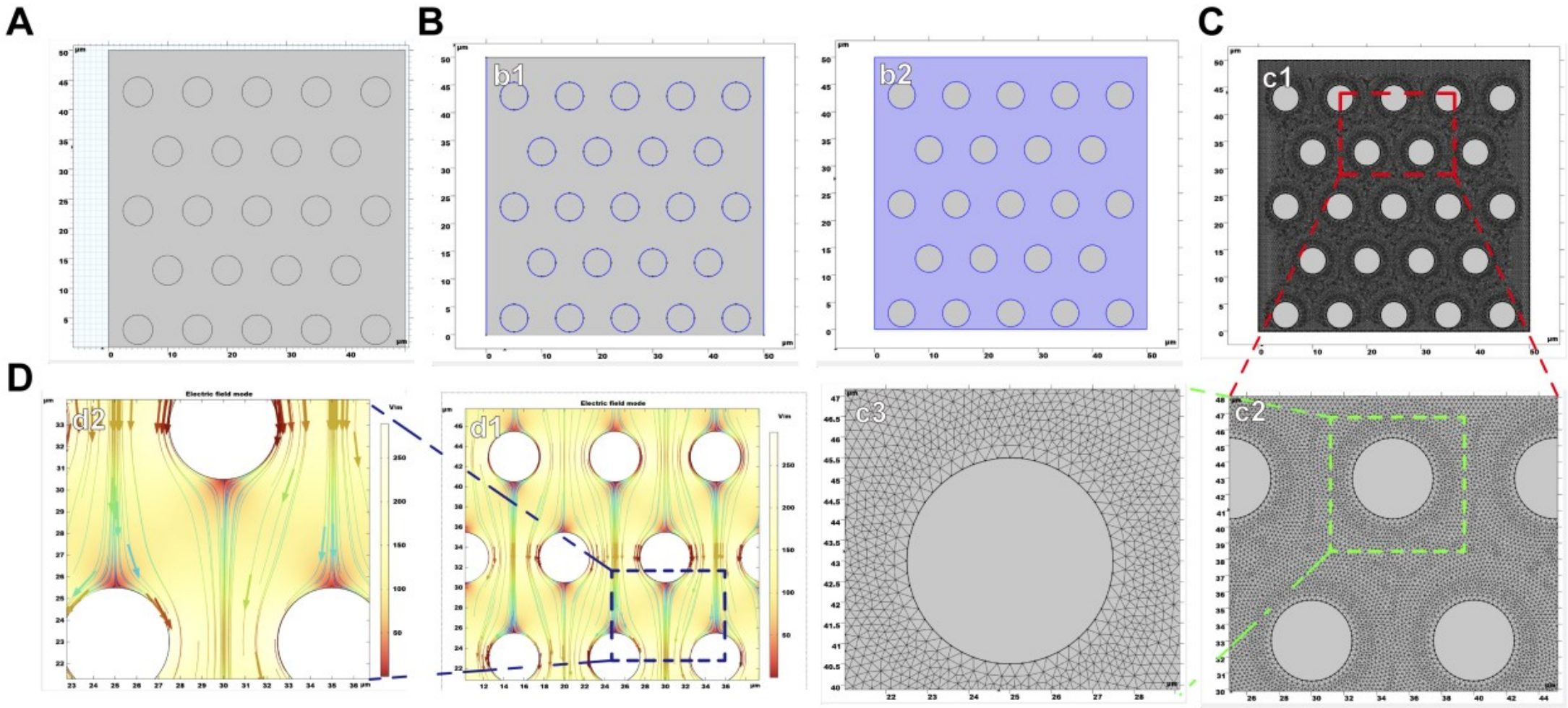


Figure S36. Workflow of the electric field line simulation in the separator based on the governing equations of the electric current physics. (a) Schematic of geometric model (RVE) construction (pores: glass fiber skeleton; background: separator matrix/electrolyte-infiltrated region). (b1) Boundary condition: electric insulation ($n \cdot \boldsymbol{J} = 0$) assigned to the lateral boundaries and the glass-fiber scaffold surfaces, with driving potentials applied at the top and bottom boundaries. (b2) Initialization of the electric potential throughout the separator domain to 0 V. (c1) Global high-resolution meshing strategy applied to the entire computational domain. (c2, c3) Magnified views of the local mesh distribution in the target region, illustrating the refined element topology. (d1) Global electric field line distribution results, obtained by solving $E = -\nabla V$ and $J = \sigma E$. (d2) High-magnification view of electric field line distribution in critical regions, revealing local orientational details of the field trajectories.

In this COMSOL simulation, the representative volume element (RVE) approach is adopted to perform a localized sampling analysis on the modified region of the battery separator. In the geometric model, the circular holes represent the glass fibers, which serve as inert insulating skeletons, while the remaining region corresponds to the separator matrix. Since the glass fibers do not participate in electrochemical reactions or ionic conduction, they are simplified as an insulating filler phase in the geometric configuration. Considering their extremely low electrical conductivity ($\sigma \approx 0$), the glass-fiber domains are excluded from the computational domain in the electric current

physics simulation to enhance computational efficiency and avoid unnecessary meshing and solving within the insulator interior. This treatment is physically equivalent to imposing a zero-ion flux boundary condition ($n \cdot \boldsymbol{J} = 0$) at the fiber/matrix interfaces, and thus does not affect the electric field distribution in the surrounding electrolyte region. The eight panels presented above systematically illustrate the complete simulation workflow, encompassing geometric model construction, boundary condition specification, meshing strategy, and post-processing of the electric field line results (**Note 1** in supporting information).

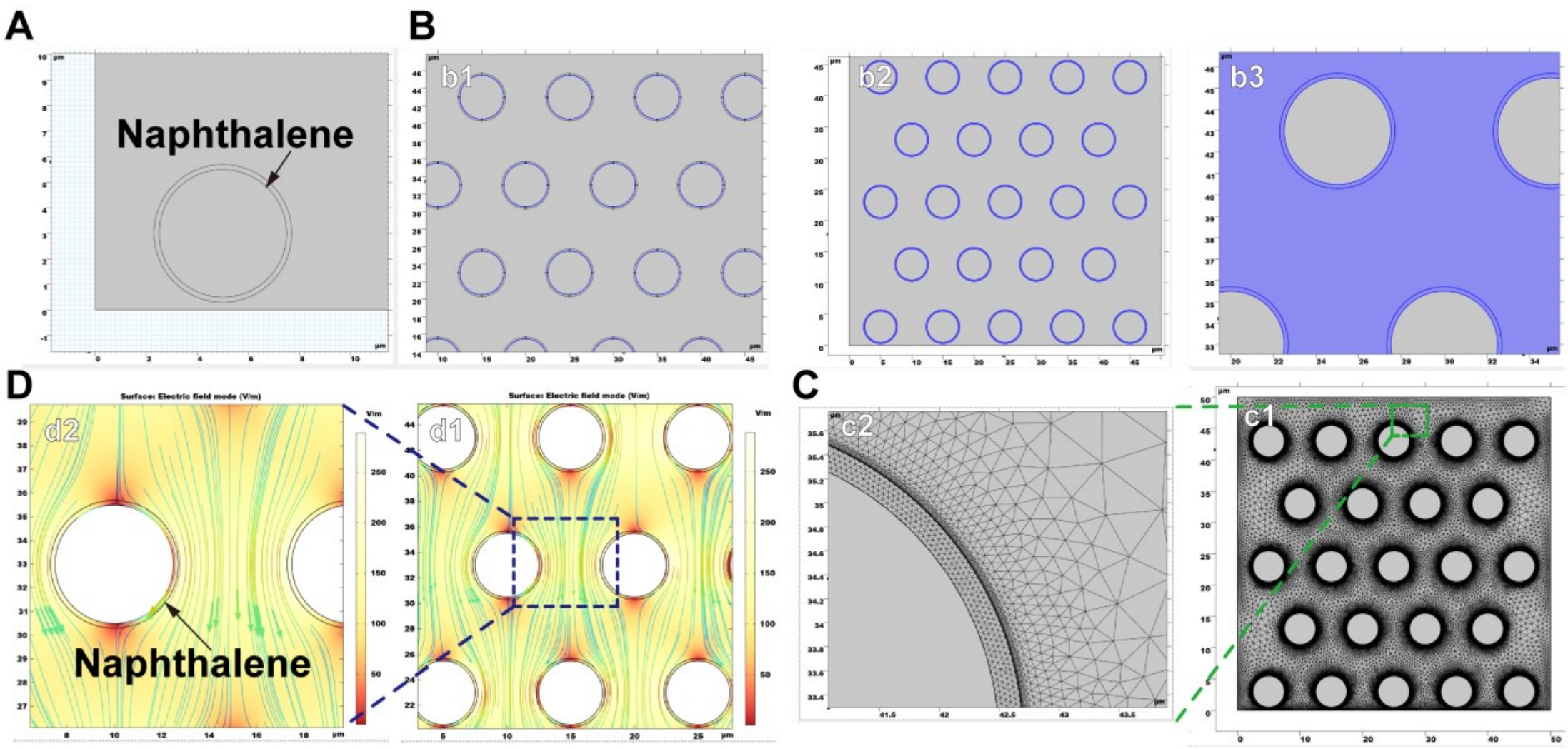


Figure S37. Workflow of the electric field line simulation in the NtGF separator based on the governing equations of the electric current physics. (a) Geometric model construction: an annular Nt modification layer is introduced to concentrically encircle each glass-fiber scaffold, with periodic arraying to establish the full geometric configuration. (b1) Boundary condition assignment: electric insulation ($n\cdot\boldsymbol{J} = 0$) is imposed at the interfaces between the glass-fiber scaffolds and the Nt rings, while driving potentials are applied to the top and bottom boundaries (with the inner boundaries of the Nt rings highlighted in blue). (b2) Material property specification: the relative permittivity ($\varepsilon_r$) and electrical conductivity ($\sigma$) of the Nt rings are independently parameterized. (b3) The separator matrix domain is assigned the corresponding material properties, with its electric response described by the current conservation equation ($\boldsymbol{J}_c = \sigma\boldsymbol{E}$). (c1) Boundary-layer refinement: high-resolution boundary layers are strategically generated at the Nt ring/matrix interfaces to clearly visualize the distribution and orientation of electric field lines in the vicinity of these interfaces. (c2) Magnified view of the boundary-layer mesh configuration around the Nt ring, corresponding to the local region demarcated by the dashed box in (c1). (d1) Local electric field line distribution within the selected region, illustrating the homogenizing effect imparted by the Nt rings. (d2) High-magnification view of the electric field line distribution, showing the detailed orientational features of the local homogenized field pattern.

This COMSOL simulation continues to adopt the representative volume element (RVE) approach to simulate the electric field distribution in the local region of the battery separator modified with Nt. In the model, the rings represent the Nt attached

onto the surface of the glass fiber skeleton, while the remaining regions represent the separator matrix. The Nt serves as a functional modification layer that does not directly participate in electrochemical reactions. However, through its dielectric/conductive properties, it redistributes and homogenizes the local electric field, thereby spreading out and homogenizing the electric field lines originally concentrated around the skeleton and ultimately improving the overall electric field uniformity of the separator. The above simulation workflow fully presents the geometric construction, boundary discretization, meshing condition settings, and post-processing results of the electric field lines for the modified model (**Note 1** in supporting information).

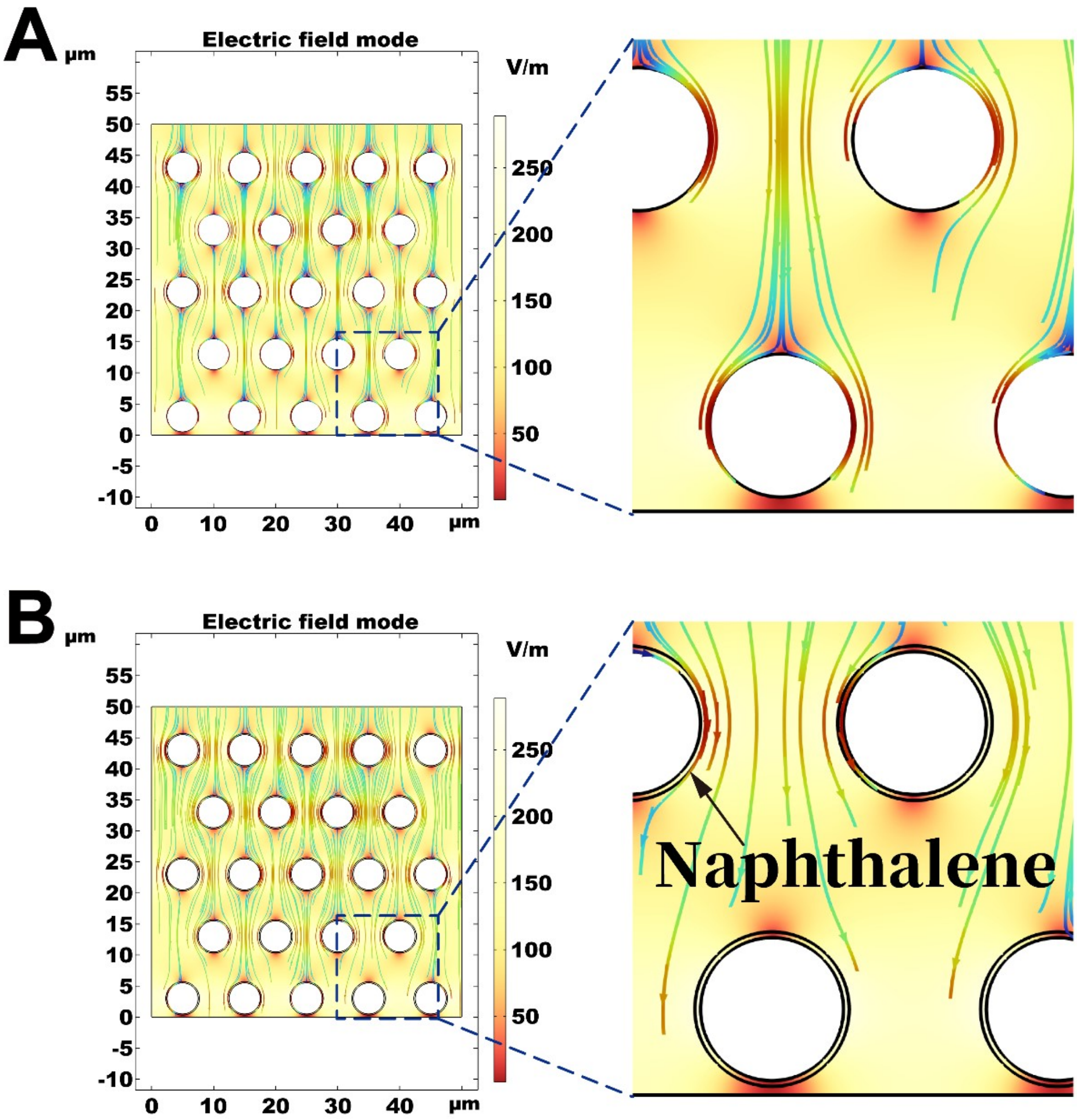


Figure S38. The partial enlarged details for $Zn^{2+}$ electrical field when used GF (A) and NtGF (B) as separators.

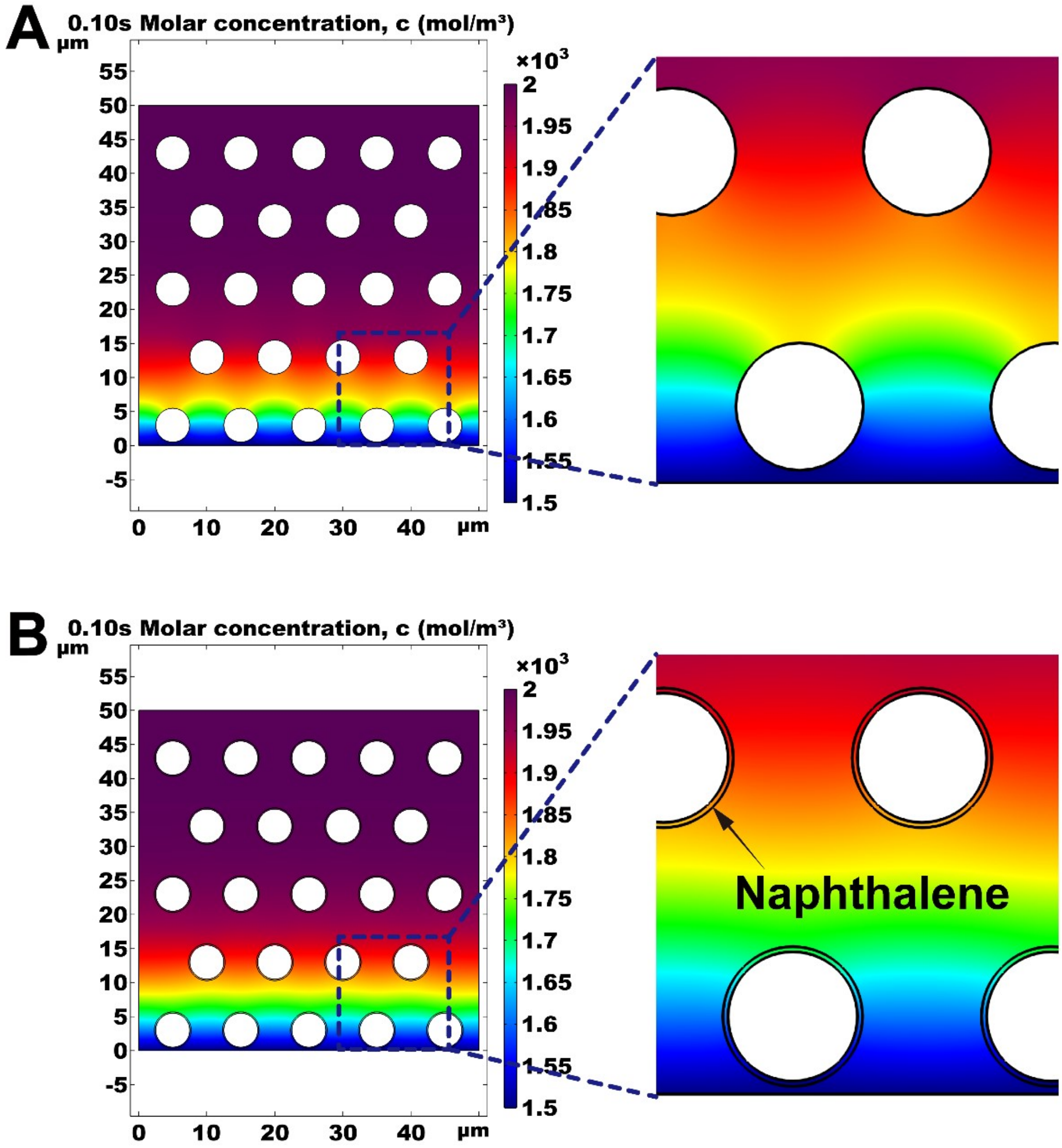


Figure S39. The partial enlarged details for $Zn^{2+}$ concentration field when used GF (A) and NtGF (B) as separators.

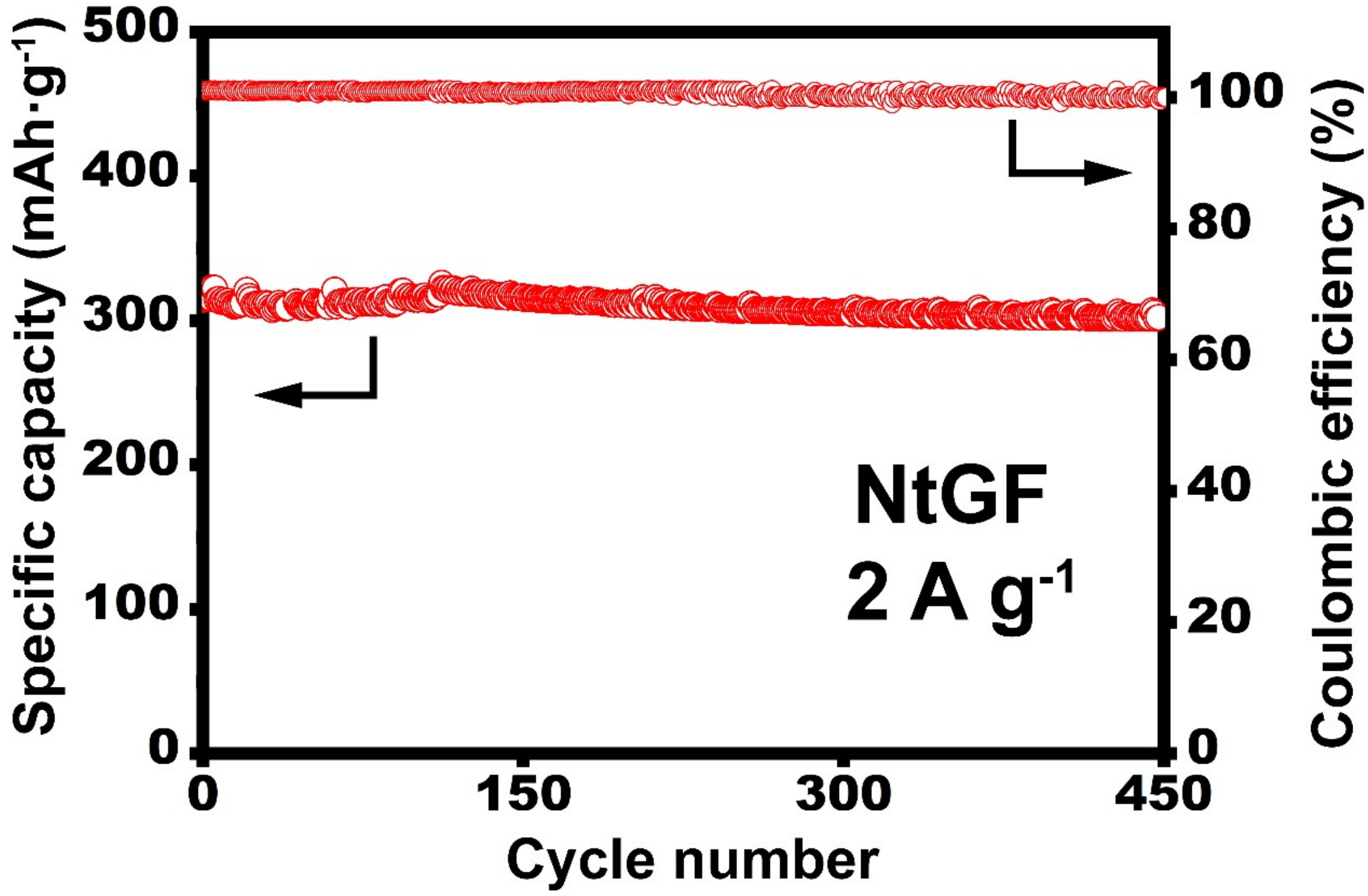


Figure S40. The long cycle stability of the Zn/NtGF/$NH_4V_4O_{10}$ battery under the current density of 2 A $g^{-1}$.

Table S1 The comparison of the electrochemical performances of NtGF separator with previously reported Zn metal anodes with separator modification.

| Separator | Cycle life (h) | Current density (mA $cm^{-2}$) | Capacity (mAh $cm^{-2}$) | Ref. |
|---|---|---|---|---|
| NtGF | 1483 | 1 | 1 | This work |
| g-$C_3N_4$/GF | 250 | 1 | 3 | [11] |
| $Ti_{0.87}O_2$ nanosheets/BC/GF | 1400 | 1 | 1 | [12] |
| cellulose nanofibers/lithium magnesium silicate | 1000 | 1 | 0.5 | [13] |
| ACOF@CNF | 350 | 1 | 1 | [14] |
| bacterial cellulose/vermiculite | 1500 | 1.169 | 1.169 | [15] |
| Zn-Nafion | 553 | 0.5 | 5 | [16] |
| PAN separator | 800 | 0.283 | - | [17] |
| NC/GF | 1100 | 1 | 1 | [18] |
| CP-GF | 1585 | 1 | 1 | [19] |
| CNF-$SO_3$Na | 500 | 1 | 0.5 | [20] |
| CT | 1200 | 1 | 0.5 | [21] |
| CSCF | 1600 | 0.5 | 0.5 | [22] |

Table S2 Comparison of long-term cycling stability of Zn/NtGF/$NH_4V_4O_{10}$ full battery between previously reported results and this work.

| Cathode | Current density (mA $cm^{-2}$) | Retained Capacity | Cycle numbers/Capacity retention (%) | Ref. |
|---|---|---|---|---|
| $NH_4V_4O_{10}$ | 5 | 266.5 | 1000/91.3 | This work |
| $NH_4V_4O_{10}$ | 5 | 253.4 | 1000/84.3 | [17] |
| $V_2O_5$ | 1 | 201.4 | 1000/80.5 | [23] |
| $V_2O_5$ | 1 | 184.6 | 600/66.2 | [12] |
| $V_2O_5$ | 2 | 296.3 | 1000/74.1 | [16] |
| VOX | 2 | ~150 | 1000/76.8 | [24] |
| MnVO | 0.3 | 288 | 500/89.4 % | [15] |
| $NH_4V_4O_{10}$ | 4 | ~130 | 850/89% | [19] |
| $NaV_3O_8\cdot 1.5H_2O$ | 10 | ~107.6 | 1000/89.7% | [25] |
| $VO_2$(B)@CFS | 2 | 153.9 | 1000/67.4% | [26] |

Table S3. Electric field simulation parameters in Figure 3.

| **Parameter** | **Figure 3D** | **Figure 3E** |
| --- | --- | --- |
| Model size (μm) | 50×50 | 50×50 |
| Separator relative permittivity ε | 80 | 80 |
| Separator electrical conductivity σ (S $m^{-1}$) | 5 | 5 |
| Nt ring relative permittivity ε | - | 3 |
| Nt ring electrical conductivity σ (S $m^{-1}$) | - | $4.35\times10^{-10}$ |
| Top boundary potential (V) | $7.4\times10^{-30}$ | $7.4\times10^{-30}$ |
| Substrate potential (V) | | |

Table S4. Concentration field simulation parameters in Figure 3.

| **Parameter** | **Figure 3F** | **Figure 3G** |
|---|---|---|
| Model size (μm) | 50×50 | 50×50 |
| Ionic charge | +2 | +2 |
| $D_{Zn^{2+}}$ | $6.5\times10^{-10}$ | $6.5\times10^{-10}$ |
| Initial concentration (mol $m^{-3}$) | 2000 | 2000 |
| Top concentration (mol $m^{-3}$) | 2000 | 2000 |
| Bottom concentration (mol $m^{-3}$) | 1500 | 1500 |

**Note 1:** The electric current distribution in the model is governed by the following set of equations:

$$\nabla \cdot J = Q_{j,v} \tag{S1}$$

where $\boldsymbol{J}$ is the total current density vector, and $Q_{j,v}$ represents the volumetric current source term (in A m$^{-3}$). This equation enforces the conservation of charge, stating that the divergence of the current density equals the net volumetric current generation or consumption rate within the domain.

$$J = \sigma E + J_e \tag{S2}$$

where $\sigma$ is the electrical conductivity of the medium (in S m$^{-1}$), $\boldsymbol{E}$ is the electric field vector, and $\boldsymbol{J_e}$ denotes an external or polarization current density (in A m$^{-2}$). The first term $\sigma\boldsymbol{E}$ describes the conduction current driven by the electric field, while $\boldsymbol{J_e}$ accounts for additional current contributions from external sources or non-ideal effects.

$$E = -\nabla V \tag{S3}$$

where V is the electric potential (in V). This equation defines the electric field as the negative gradient of the electric potential, reflecting the physical principle that the electric field points in the direction of decreasing potential.

**Note 2**: The spatiotemporal evolution of ionic concentration field is described by the species transport equation:

$$\frac{\partial c_i}{\partial t} + \nabla \cdot J_i + u \cdot \nabla c_i = R_i \tag{S4}$$

where $c_i$ denotes the molar concentration of ionic species $i$, $t$ is time, $\boldsymbol{J}_i$ is the diffusive-migratory molar flux, $u$ represents fluid convective velocity, and $R_i$ stands for the volumetric source/sink term accounting for species generation or consumption via electrochemical reactions. The ionic flux $\boldsymbol{J}_i$ follows the Nernst–Planck equation:

$$J_i = -D_i \nabla c_i - z_i u_{m,i} F c_i \nabla V \tag{S5}$$

In this expression, $D_i$ is the diffusion coefficient of species $i$; $z_i$ is the charge number of the ion; $u_{m,i}$ is the ionic mobility; $F$ is Faraday's constant; and $V$ refers to the local electric potential. The first term $-D_i \nabla c_i$ corresponds to molecular diffusion driven by concentration gradients. The second term describes ion migration induced by the electric potential gradient.

**Reference**


1 M. J. Frisch, G. W. Trucks, H. B. Schlegel, G. E. Scuseria, M. A. Robb, J. R. Cheeseman, G. Scalmani, V. Barone, G. A. Petersson, H. Nakatsuji, X. Li, M. Caricato, A. V. Marenich, J. Bloino, B. G. Janesko, R. Gomperts, B. Mennucci, H. P. Hratchian, J. V. Ortiz, A. F. Izmaylov, J. L. Sonnenberg, Williams, F. Ding, F. Lipparini, F. Egidi, J. Goings, B. Peng, A. Petrone, T. Henderson, D. Ranasinghe, et al., Gaussian 16 A.03. Wallingford, CT, 2016.

2 F. Weigend and R. Ahlrichs, *Phys. Chem. Chem. Phys.*, 2005, 7, 3297-3305.

3 A. D. Becke, *J. Chem. Phys.*, 1993, 98, 5648-5652.

4 C. Lee, W. Yang and R. G. Parr, *Phys. Rev. B*, 1988, 37, 785-789.

5. S. Grimme, J. Antony, S. Ehrlich and H. Krieg, *J. Chem. Phys.*, 2010, 132, 154104.

6 S. Grimme, S. Ehrlich and L. Goerigk, *J. Comput. Chem.*, 2011, 32, 1456-1465.

7 A. V. Marenich, C. J. Cramer and D. G. Truhlar, *J. Phys. Chem. B*, 2009, 113, 6378-6396.

8 T. Lu and F. Chen, *J. Comput. Chem.*, 2012, 33, 580-592.

9 T. Lu and Q. Chen, *J. Comput. Chem.*, 2022, 43, 539-555.

10 W. Humphrey, A. Dalke and K. Schulten, *J. Mol. Graph.*, 1996, 14, 33-38.

11 Y. Yang, T. Chen, B. Yu, M. Zhu, F. Meng, W. Shi, M. Zhang, Z. Qi, K. Zeng, and J. Xue, *Chem. Eng. J.*, 2022, 433, 134077.

12 H. Tan, C. Wang, X. Li, N. Sakai, Z. Zhang, H. Yao, Y. Han, Z. Lin, J. Zhou, J. Xu, T. Mei, X. Wang, R. Ma, T. Sasaki, G. Wang, and Y. Chen, *Small*, 2025, e10596.

13 J. Cao, D. Zhang, R. Chanajaree, D. Luo, X. Yang, X. Zhang, J. Qin, *Chem. Eng. J.*, 2024, 480, 147980.

14 C. Huang, M. Tian, G. Jin, Y. Zou, Y. Wang, J. Hu, Y. He, Y. Li and J. Sun, *Chem. Commun.*, 2026, 62 (25): 6813-6816.

15 T. Li, Y. Gong, H. Yang, Y. Liu, X. Dong, Y. Xu, H. Ma, C. Wei, S. Zhang, F. Huang, M. Yang, T. Lin, *Energy Storage Mater.*, 2025, 79, 104311.

16 B. Wu, Y. Wu, Z. Lu, J. Zhang, N. Han, Y. Wang, X. Li, M. Lin and L. Zeng, *J. Mater. Chem. A*, 2021, 9 (8): 4734-4743.

17 Y. Fang, X. Xie, B. Zhang, Y. Chai, B. Lu, M. Liu, J. Zhou, and S. Liang, *Adv. Funct. Mater.*, 2022, 32, 2109671.

18 X. Yang, W. Li, J. Lv, G. Sun, Z. Shi, Y. Su, X. Lian, Y. Shao, A. Zhi, X. Tian, X. Bai, Z. Liu and J. Sun, *Nano Res.*, 2022, 15, 9785-9791.

19 Z. Yang, W. Li, Q. Zhang, C. Xie, H. Ji, Y. Tang, Y. Li, H. Wang, *Mater. Today Energy*, 2022, 28, 101076.

20 X. Ge, W. Zhang, F. Song, B. Xie, J. Li, J. Wang, X. Wang, J. Zhao, and G. Cui, *Adv. Funct. Mater.*, 2022, 2200429.

21 P. Cao, H. Zhou, X. Zhou, Q. Du, J. Tang, and J. Yang, ACS Sustainable Chem. Eng., 2022, 10, 26, 8350-8359.

22 X. Hu, R. Li, L. Du, W. Song, Q. Lei, S. Zou, P. Wang, M. Zhu b, Z. Gong, Y. Wu, Y. Zhang and F. Shi, Electrochim. Acta, 2025, 542, 147531.

23 H. Qin, W. Chen, W. Kuang, N. Hu, X. Zhang, H. Weng, H. Tang, D. Huang, J. Xu, and H. He, *Small*, 2023, 19, 2300130.

24 F. Shen, H. Du, H. Qin, Z. Wei, W. Kuang, N. Hu, W. Lv, Z. Yi, D. Huang, Z. Chen, and H. He, *Small*, 2023, 2305119.

25 J. Ma, X. Shi, Z. Wang, L. Zhou, X. Liu, X. Lu, and Z. Jiang, *Adv. Mater.*, 2024, 36, 2406429.

26 Y. Ran; H. Zhao and Y. Lei, *J. Mater. Chem. A*, 2026, 14(15): 8452-8482.
